# Dietary pattern modifies the risk of MASLD through metabolomic signature

**DOI:** 10.1016/j.jhepr.2024.101133

**Published:** 2024-06-10

**Authors:** Hanzhang Wu, Jiahe Wei, Shuai Wang, Liangkai Chen, Jihui Zhang, Ningjian Wang, Xiao Tan

**Affiliations:** 1 Department of Big Data in Health Science, Zhejiang University School of Public Health, Hangzhou, China. Sir Run Run Shaw Hospital, Zhejiang University School of Medicine, Hangzhou, China; 2 The Key Laboratory of Intelligent Preventive Medicine of Zhejiang Province, Hangzhou, China; 3 Department of Nutrition and Food Hygiene, Hubei Key Laboratory of Food Nutrition and Safety, School of Public Health, Tongji Medical College, Huazhong University of Science and Technology, Wuhan, China; 4 Center for Sleep and Circadian Medicine, The Affiliated Brain Hospital of Guangzhou Medical University, Guangzhou, China; Department of Psychiatry, Faculty of Medicine, The Chinese University of Hong Kong, Hong Kong Special Administrative Region, China; 5 Institute and Department of Endocrinology and Metabolism, Shanghai Ninth People's Hospital, Shanghai Jiao Tong University School of Medicine, Shanghai, China; 6 Department of Medical Sciences, Uppsala University, Uppsala, Sweden

**Keywords:** MASLD, EAT-lancet diet, Metabolite signature, Dietary metabolic response, Polygenic risk score

## Abstract

**Background & Aims:**

The EAT-Lancet Commission in 2019 advocated a plant-centric diet for health and environmental benefits, but its relation to metabolic dysfunction-associated steatotic liver disease (MASLD) is unclear. We aimed to discover the metabolite profile linked to the EAT-Lancet diet and its association with MASLD risk, considering genetic predisposition.

**Methods:**

We analyzed data from 105,752 UK Biobank participants with detailed dietary and metabolomic information. We constructed an EAT-Lancet diet index and derived a corresponding metabolomic signature through elastic net regression. A weighted polygenic risk score for MASLD was computed from associated risk variants. The Cox proportional hazards model was employed to estimate hazard ratios (HRs) and 95% CIs for the risk of MASLD (defined as hospital admission or death).

**Results:**

During a median follow-up period of 11.6 years, 1,138 cases of MASLD were documented. Participants in the highest group for the EAT-Lancet diet index had a multivariable HR of 0.79 (95% CI 0.66–0.95) for MASLD compared to the lowest group. The diet's impact was unaffected by genetic predisposition to MASLD (*p* = 0.42). Moreover, a robust correlation was found between the metabolomic signature and the EAT-Lancet diet index (Pearson r = 0.29; *p* <0.0001). Participants in the highest group for the metabolomic signature had a multivariable HR of 0.46 (95% CI 0.37–0.58) for MASLD, in comparison to those in the lowest group.

**Conclusions:**

Higher intake of the EAT-Lancet diet and its associated metabolite signature are both linked to a reduced risk of MASLD, independently of traditional risk factors.

**Impact and implications:**

Our analysis leveraging the UK Biobank study showed higher adherence to the EAT-Lancet diet was associated with a reduced risk of metabolic dysfunction-associated steatotic liver disease (MASLD). We identified a unique metabolite signature comprising 81 metabolites associated with the EAT-Lancet diet, potentially underlying the diet's protective mechanism against MASLD. These findings suggest the EAT-Lancet diet may offer substantial protective benefits against MASLD.

## Highlights

• The EAT-Lancet diet promotes both human and environmental health.
• Elastic net regression identified a metabolic signature consisting of 81 metabolites.
• High EAT-Lancet diet index was associated with a lower risk of MASLD regardless of genetic susceptibility.
• Metabolic signature associated with the EAT-Lancet diet was inversely related to MASLD.

## Graphical abstract

**Figure:**
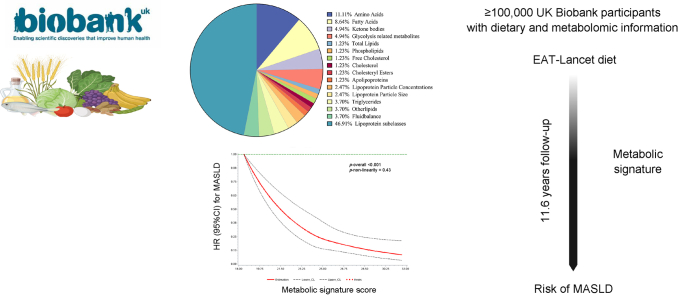

## Introduction

Over the past 40 years, metabolic dysfunction-associated steatotic liver disease (MASLD) has become the most widespread chronic liver disorder worldwide,^1^ not only standing as the predominant liver disease globally but also as the principal contributor to liver-related morbidity and mortality.^2^^,^^3^ The global prevalence of MASLD is estimated to be 32.4%.^4^ Despite the lack of approved therapies or specific pharmacological treatments for MASLD, adopting healthy lifestyles, such as dietary modifications and regular physical activity, is highly recommended as a preventive strategy against its onset.[5], [6], [7]

Several studies have explored how different diets affect MASLD.[8], [9], [10], [11] The EAT-Lancet diet was proposed in 2019 by the EAT-Lancet Commission, which is designed as a universal dietary guideline aimed at benefiting human health and environmental sustainability.^12^ It emphasizes whole grains, vegetables, fruits, legumes, and nuts, while limiting meat, sugar, and saturated fats. Unlike previous plant-based diets that completely exclude animal source foods,^10^ the EAT-Lancet diet includes healthy animal foods like dairy and fish, offering a balanced approach. This flexibility may make it a more feasible and appealing option for individuals who prefer to include animal products in their diet. However, there is currently insufficient evidence regarding the specific impact of the EAT-Lancet diet on MASLD.

Moreover, diet can influence disease risk by modulating metabolic pathways and homeostasis.^13^ In recent years, high-throughput metabolomic profiling has allowed for the quantitative analysis of biomarkers across multiple biological pathways, assessing genetic, environmental, and pathological changes during disease development in targeted tissues or biological fluids. Several prior studies have conducted metabolomic analyses of plasma to gain a comprehensive understanding of the alterations across various metabolic pathways in patients with MASLD.[14], [15], [16] These studies have uncovered metabolic signatures spanning the spectrum of MASLD and reveal that elevated levels of circulating triglycerides and an increase in large HDL particles, as well as a deficiency in polyunsaturated fatty acid content, may be an important risk factor for incident MASLD. This advance in metabolomic profiling has also enabled the objective assessment of overall adherence and metabolic responses to complex dietary patterns. However, it remains unclear whether the EAT-Lancet diet offers a protective effect against MASLD and the detailed mechanisms by which it may confer such protection through the modulation of metabolic pathways are still not fully understood.

Furthermore, the influence of genetic predisposition on the development of MASLD is widely acknowledged.^17^ In recent years, numerous studies have indicated that genetic susceptibility interacts with lifestyle factors in the development of MASLD.^18^^,^^19^ However, it remains unclear whether there is an interaction between the EAT-Lancet diet and genetic susceptibility regarding the risks of MASLD.

In the present study, we aimed to identify metabolic signatures indicative of adherence to the EAT-Lancet diet, and assessed whether the EAT-Lancet diet and its metabolic signatures were associated with subsequent MASLD. Additionally, we explored whether genetic predisposition could influence these associations.

## Patients and methods

### Study population

The UK Biobank is an ongoing population-based cohort study established between 2006 and 2010.^20^ It recruited over 500,000 general participants, aged 39 to 72 years, from 22 assessment centers across England, Scotland, and Wales. Detailed information on the study population of the UK Biobank study is presented in the Supplementary Methods.

In this particular study, we included 113,800 participants who had metabolite data, and completed the web-based 24-h dietary assessments (the Oxford WebQ) at least once. For the analysis of metabolite signature with incident MASLD, we excluded participants who had missing data on any covariates (n = 408) or those with implausible energy intake (<800 or >4,200 kcal/day in men and <600 or >3,500 kcal/day in women, n = 2,233). Additionally, participants with MASLD, alcohol-related liver diseases, viral hepatitis, autoimmune hepatitis, cirrhosis, liver cancer, other liver diseases, and alcohol/drug use disorder at baseline (n = 1,067) were also excluded. Considering that single-nucleotide polymorphisms (SNPs) are significantly associated with MASLD in participants of European descent, we ultimately excluded those who were not of European descent or had missing data on genetic information (n = 4,340). Consequently, our final study comprised 105,752 participants (Fig. S1).

### Dietary assessment and EAT-Lancet diet index

In the UK Biobank, dietary information was gathered using the Oxford WebQ, a web-based tool for 24-hour recalls, designed specifically for large-scale population studies. Participants were instructed to provide the consumption of over 200 food types and more than 30 drink varieties for one specific day at a time.^21^ To ensure a comprehensive dietary assessment covering various seasons, the same questionnaire was sent to all participants on four distinct occasions between February 2011 and April 2012. The Oxford WebQ has been validated through comparison with an interviewer-administered 24-hour recall and biomarkers.^22^^,^^23^ Our previous study demonstrated the method for calculating the average daily intake of various food items (g/day).^24^ Details on how the EAT-Lancet diet index was calculated are provided in the Supplementary Methods.

### Metabolomics measurement

All metabolite values underwent a rank-based inverse normal transformation, which consists of a two-step process. First, the observations were transformed to the probability scale using the empirical cumulative distribution function. Then, the transformed observations were converted to Z-scores on the real number line using the probit function. Participants were randomly assigned to either the training set or the testing set (5 to 5 ratio) to identify metabolite signatures that are correlated with the EAT-Lancet diet index. In the training set, the elastic net model within a tenfold cross-validation framework was used to regress the EAT-Lancet diet index on the 170 named metabolites.^25^ The trained model was applied to the testing sets to calculate the metabolic signature. This signature was obtained by taking the weighted sum of the selected metabolites, where the weights were determined by the coefficients from the elastic net regression. The score in the training set was derived using a leave-one-out cross-validation approach to avoid overfitting. In order to evaluate the robustness of the chosen metabolites in the metabolite signature score, we computed correlation coefficients between the overall diet index and the corresponding metabolite signature scores in the training set, test set and the combined data. Multivariable linear regression was employed to examine the associations between selected metabolites and diet index, as well as dietary components. Prior to the regression analyses, EAT-Lancet diet index and intake of each dietary component were standardized to ensure comparability. Moreover, we also used the repeated measurements data from Phase 2 NMR of metabolic biomarkers to verify our result. Details on the information of metabolomics were provided in the Supplementary Methods.

### Assessment of MASLD

The assessment of MASLD was based on hospital inpatient records and death registry data. The diagnoses were recorded according to ICD-9 and ICD-10^26^ (Table S3). For this study, the updates for hospital inpatient admission linkages were as follows: 31 October 2022 in England, 31 August 2022 in Scotland, and 31 May 2022 in Wales. The person-years of follow-up for each participant in the UK Biobank were calculated starting from the date of completing the Oxford WebQ dietary assessment until the date of incident MASLD, death, loss to follow-up, or the end of the follow-up period, whichever occurred first.

### Polygenic risk score for MASLD

The genetic data of the UK Biobank study underwent a detailed genotyping process, imputation, and quality control, as described in previous publications.^27^ For this study, a subset of five SNPs significantly associated with MASLD in participants of European descent (rs738409, rs58542926, rs641738, rs1260326, and rs72613567)^28^ (Table S4). Based on the selected SNPs, the polygenic risk score (PRS) for MASLD was calculated.

### Ascertainment of covariates

Information on age, sex, educational level, and smoking status was assessed through a self-reported questionnaire at the baseline investigation. The Townsend deprivation index was derived from the postcode of residence using aggregated data on unemployment, car and homeownership, and household overcrowding.^29^ Physical activity was estimated in metabolic equivalent minutes per week. BMI was calculated by dividing weight in kilograms by height in meters squared. Alcohol consumption and total energy intake were estimated using data from 24-hour dietary recalls.

### Statistical analysis

Baseline characteristics of the study participants were expressed as mean (SD) and categorical variables are expressed as percentages. Cox proportional hazards regression models were employed to examine the hazard ratios (HRs) and their corresponding 95% CIs in relation to the associations between the EAT-Lancet diet index, metabolite signature score, selected metabolites, and the risk of MASLD. The proportional hazards assumption was tested by Schoenfeld residuals, and no violation of this assumption was found in our analyses. We also employed restricted cubic spline analyses with three knots (25th, 50th, and 75th) to examine the dose-response association. The *p* value for interactions were calculated by including multiplicative interaction terms of the EAT-Lancet diet index and PRS categories in the fully adjusted model.

We conducted several sensitivity analyses to ensure the robustness of our primary findings. First, to assess the impact of individual components on the association between the overall EAT-Lancet diet score and MASLD, we recalculated the EAT-Lancet diet scores by excluding each component and examined the association between the recalculated score and the risk of developing MASLD. Second, we further adjusted for liver function to account for the confounding effect of baseline liver function. Third, we further adjusted for waist circumference to minimize the confounding effect of central obesity. Fourth, we excluded participants with less than two dietary assessments to ensure the reliability of the dietary data used in our analysis. Fifth, we performed subgroup analyses to assess the potential effect modifications by age, sex, BMI, education level, physical activity, alcohol intake, smoking status, hypertension, and diabetes. Sixth, we expanded the definition of MASLD to encompass a more inclusive set of criteria, including K76.8 (other specified diseases of the liver), K76.9 (unspecified liver disease), and other unspecified liver conditions such as K74.0 (hepatic fibrosis), K74.1 (hepatic sclerosis), K74.2 (hepatic fibrosis with hepatic sclerosis), and K74.6 (other and unspecified cirrhosis of the liver). Finally, we excluded MASLD cases that occurred within the first 3 years of follow-up to assess whether the results might have been influenced by reverse causation bias.

A two-sided *p* <0.05 was considered statistically significant and multiple testing was corrected using the Bonferroni method. All statistical analyses were conducted using SAS version 9.4 (SAS Institute Inc., Gary, NC, USA) and R software version 4.3.1.

## Results

A total of 105,752 participants were enrolled in this study, and they were observed for a median duration of 11.6 years, totaling 1,213,582 person-years of follow-up. During this period, 1,138 cases of MASLD were recorded. Table 1 presents the characteristics of the participants based on categories of the EAT-Lancet diet index.

**Table 1:** Baseline characteristics of the study participants according to quartiles of the EAT-Lancet diet index.^a^

Characteristics	Categories of the EAT-Lancet diet index				*p* value^b^
	Quartile 1	Quartile 2	Quartile 3	Quartile 4	
EAT-Lancet diet index (min, max)	9-21	22-24	25-27	28-41	
No. of participants	23,887	28,483	27,171	26,211	
Age (years)	55.5 (8.00)	56.3 (7.88)	56.6 (7.84)	56.8 (7.70)	<0.0001
Sex (male, %)	53.6	47.2	42.7	38.3	<0.0001
Body mass index (kg/m^2^)	27.8 (4.77)	27.3 (4.65)	26.8 (4.52)	26.0 (4.34)	<0.0001
Townson depretive index	-1.64 (2.85)	-1.79 (2.75)	-1.86 (2.73)	-1.76 (2.77)	<0.0001
Education level (college or higher, %)	35.0	39.1	43.5	48.8	<0.0001
PA (MET × hour/week)	38.8 (40.9)	40.2 (39.9)	42.6 (40.4)	44.8 (40.7)	<0.0001
Total energy intake (kCal/day)	2,015.7 (625.0)	2,020.9 (602.9)	2,049 (593.1)	2,108.8 (591.5)	<0.0001
Alcohol intake (g/day)	20.6 (28.2)	17.4 (24.7)	16.1 (23.1)	14.4 (21.2)	<0.0001
Smoking status (%)					<0.0001
Current smoker	11.1	7.70	6.26	5.03	
Ex-smoker	35.9	35.9	36.0	36.4	
Non-smoker	53.1	56.4	57.8	58.6	
Individual history of disease (%)					
Hypertension	25.2	24.1	22.7	21.4	<0.0001
Diabetes	3.47	3.28	3.17	2.63	<0.0001
Cardiovascular disease	1.50	1.60	1.51	1.57	0.75
Cancer	10.2	10.6	10.5	11.2	<0.01
Glucose	5.12 (1.16)	5.12 (1.16)	5.10 (1.08)	5.06 (1.02)	<0.0001
HbA1c	35.7 (6.17)	35.7 (6.17)	35.5 (5.99)	35.3 (5.69)	<0.0001
Cholesterol	5.70 (1.13)	5.70 (1.13)	5.73 (1.13)	5.72 (1.11)	0.01
ALT	24.4 (14.2)	24.4 (14.2)	22.6 (13.2)	21.7 (12.1)	<0.0001

ALT, alanine aminotransferase; MET, metabolic equivalent; MASLD, metabolic dysfunction-associated steatotic liver disease; PA, physical activity.

a Continuous variables are expressed as mean (SD) and categorical variables are expressed as percentages.

b Chi-squared was used for categorical variables and one-way analysis of variance for continuous variables. A two-tailed *p* <0.05 was considered statistically significant.

**Fig. 1:**
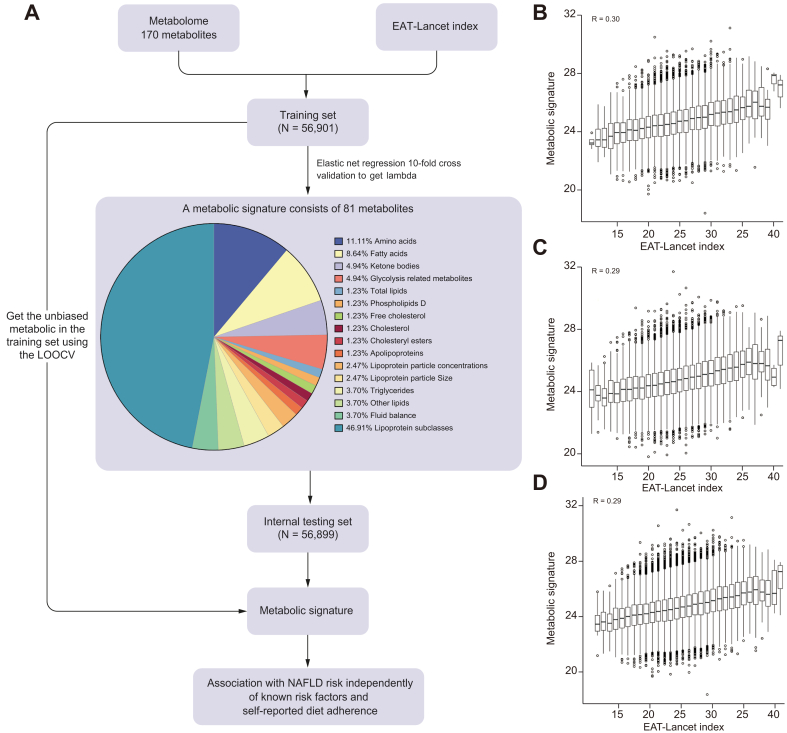
The metabolic signature for adherence to the EAT-Lancet diet: flow chart for analytic approach and validation. (A) The training and testing procedures of a metabolic signature for the EAT-Lancet diet. Correlation between the EAT-Lancet diet and (B) the corresponding metabolite signature score in the training set (C) the corresponding metabolite signature score in the testing set (D) the corresponding metabolite signature score in the combined data. Pearson correlation is employed in correlation analysis. A two-tailed *p* <0.05 was considered statistically significant. LOOCV, leave-one-out cross-validation; MASLD, metabolic dysfunction-associated steatotic liver disease.

Out of the 170 metabolic biomarkers initially included in the analysis, the elastic net regression identified a metabolic signature consisting of 81 metabolites (47.6%). These metabolites exhibited a significant association with the EAT-Lancet diet at baseline and were robust against the effects of collinearity between metabolites. Among the metabolites in the signature, the majority were lipoprotein subclasses (n = 38), while amino acids (n = 9) and fatty acids (n = 7) were also included (Fig. 1A). The metabolite signature score, based on the selected metabolites, showed a significant correlation with the EAT-Lancet diet at baseline (Fig. 1B-D; all *p <*0.0001).

**Fig. 2:**
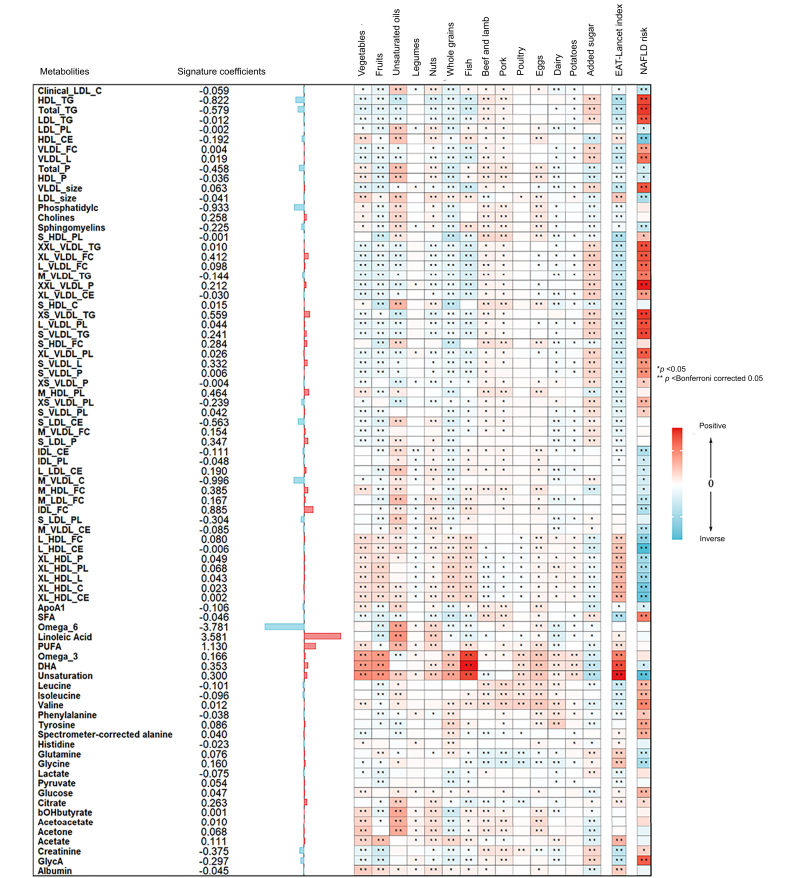
Associations of the metabolic signature with the EAT-Lancet diet components, total EAT-Lancet diet index, and subsequent MASLD disease risk. Presented from left to right are the metabolites’ coefficients (weights) in the signature, associations with each food component, total EAT-Lancet diet index, and subsequent MASLD risk. Coefficients for associations with food items and total EAT-Lancet diet index indicate the SD changes in metabolites per SD increment in dietary intake. Coefficients for MASLD risk indicate hazard ratio of MASLD disease risk per SD increment in metabolites. The weights in the signature were evaluated using elastic net regression. Linear regressions were used to analyze the connection between metabolites, food components, and the total EAT-Lancet diet index. Cox regressions were utilized to analyze the association between metabolites and the risk of MASLD. All models were adjusted similarly to Model 2 in Table 2. Colors indicate the direction (red for positive and blue for inverse) and strength (darker color for stronger magnitude) of the associations; asterisks denote significance (∗*p* <0.05 and ∗∗Bonferroni corrected *p* <0.05; for associations with total MEDAS score and MASLD risk, we Bonferroni corrected for 81 metabolites; for associations with each food item, we Bonferroni corrected for 81 metabolites × 14 food items). MASLD, metabolic dysfunction-associated steatotic liver disease.

**Fig. 3:**
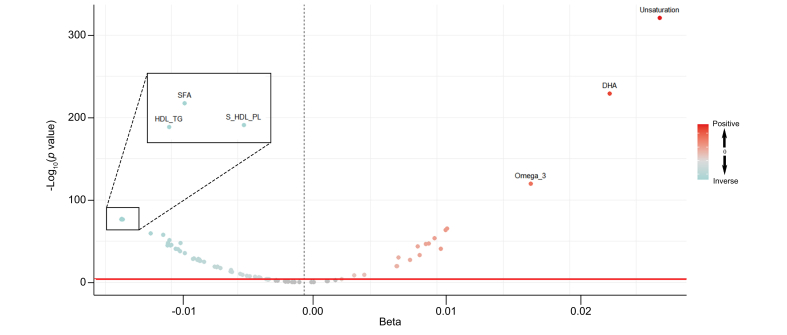
Volcano plot of association between the EAT-Lancet diet index and 81 metabolites in full sample analysis. Linear regressions were adjusted in the same way as for Model 2 in Table 2. Green and red dots represent significant relationship (*p* value <0.05/81), grey dots represent non-significant relationship. MASLD, metabolic dysfunction-associated steatotic liver disease.

Metabolites that positively correlated with the EAT-Lancet diet index, such as omega-3 fatty acids, docosahexaenoic acid, cholesteryl esters in very large HDL, and cholesterol in very large HDL, were also positively associated with the intake of emphasized foods such as fish, vegetables, and fruits (Fig. 2). Conversely, metabolites that inversely correlated with the EAT-Lancet diet index, such as saturated fatty acids (SFAs), triglycerides in HDL, and phospholipids in small HDL, were found to be positively associated with the consumption of beef, pork, and added sugar. Among the 81 metabolite markers, the degree of unsaturation in fatty acids emerged as the factor most significantly associated with the EAT-Lancet diet index (Fig. 3).

Table 2 demonstrates the prospective analyses of the EAT-Lancet diet index, metabolic signature, and the risk of MASLD. In the multivariable adjusted models, the HRs (95% CIs) for MASLD incidence across quartiles 2-4 of the EAT-Lancet diet index were 1.04 (0.89–1.20), 0.84 (0.71–0.99), and 0.79 (0.66–0.95), respectively. Likewise, the metabolite signature score showed an inverse association with MASLD incidence even after adjustment for multiple variables. The HRs (95% CI) for MASLD across quartiles 2-4 of the metabolite signature score were 0.74 (0.64–0.86), 0.61 (0.52–0.73), and 0.46 (0.37–0.58) respectively. The dose-response analysis shows that the EAT-Lancet diet index and metabolite signature score had a linear association with MASLD risk (*p* for non-linear >0.05 for EAT-Lancet diet index and metabolite signature score) (Fig. S3). Further adjustment for the EAT-Lancet diet index and metabolic signature simultaneously only slightly attenuated the association of the metabolite signature score with MASLD risk. However, the inverse associations of the EAT-Lancet diet index with MASLD risk became noticeably weaker and no longer statistically significant after adjusting for the metabolite signature score.

**Table 2:** Association of the EAT-Lancet diet index and metabolic signature with MASLD risk.^a^

	Categories of the EAT-Lancet diet index				*p* for trend^c^
	Quartile 1	Quartile 2	Quartile 3	Quartile 4	
**EAT-Lancet diet index**					
No. of MASLD	327	359	252	200	
Person-years	273,052	326,585	312,203	301,743	
Incidence per 1,000 PYs	1.20	1.10	0.81	0.66	
Model 1	1.00 (reference)	0.99 (0.86–1.16)^b^	0.79 (0.67–0.93)	0.74 (0.62–0.88)	<0.0001
Model 2	1.00 (reference)	1.04 (0.89–1.20)	0.84 (0.71–0.99)	0.79 (0.66–0.95)	<0.001
Model 2 + mutual adjustment	1.00 (reference)	1.06 (0.91–1.23)	0.88 (0.75–1.05)	0.87 (0.73–1.05)	0.05
**Metabolic signature**					
No. of MASLD	524	306	198	110	
Person-years	306,218	304,873	303,047	299,444	
Incidence per 1,000 PYs	1.71	1.00	0.65	0.37	
Model 1	1.00 (reference)	0.73 (0.63–0.84)	0.58 (0.49–0.69)	0.43 (0.35–0.54)	<0.0001
Model 2	1.00 (reference)	0.74 (0.64–0.86)	0.61 (0.52–0.73)	0.46 (0.37–0.58)	<0.0001
Model 2 + mutual adjustment	1.00 (reference)	0.75 (0.65–0.87)	0.63 (0.52–0.75)	0.48 (0.38–0.60)	<0.0001

Model 1 was adjusted for age, sex, and BMI.

Model 2 was additionally adjusted for total energy intake, smoking status, alcohol intake, educational level, Townsend deprivation index, physical activity, hypertension, diabetes, cancer, cardiovascular disease, fasting duration, spectrometer, MASLD-PRS, first 10 principal components of ancestry, and genotype measurement batch.

Model 2 + mutual adjustment additionally included both EAT-Lancet diet index and the metabolic signature of EAT-Lancet diet simultaneously in model 2 to examine association independence.

MASLD-PRS, metabolic dysfunction-associated steatotic liver disease-polygenic risk score; PYs, person-years.

a Obtained by using multivariable Cox regression model.

b Hazard ratios (95% confidence interval) (all such values).

c *p* for trend was calculated across quartiles using multivariable Cox regression models. A two-tailed *p* <0.05 was considered statistically significant.

**Fig. 4:**
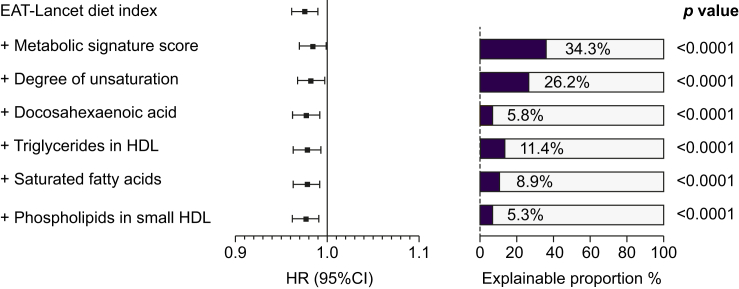
Associations of the EAT-Lancet diet index with the risk of MASLD, adjusting for intermediate metabolites. All mediation analysis models were adjusted in the same way as for Model 2 in Table 2. A two-tailed *p* <0.05 was considered statistically significant. HR, hazard ratio; MASLD, metabolic dysfunction-associated steatotic liver disease.

Furthermore, a mediation analysis was conducted to estimate the proportion of MASLD association that could be explained by the metabolite signature score and six significant metabolic biomarkers in the volcano plot. The association between the EAT-Lancet diet index and MASLD was 34.3% (95% CI 16.6%-57.9%) mediated by the metabolic signature. Moreover, the proportion of this association between the EAT-Lancet diet index and MASLD that could be explained by these individual metabolic biomarkers ranged between 5.3% and 26.2% (Fig. 4).

Participants with high PRS had a significantly increased risk of incident MASLD compared to those with low PRS (multivariate-adjusted HR of 1.90, 95% CI 1.57–2.30) (Table S5). Stratified analyses by PRS showed that the associations between a higher EAT-Lancet diet index and lower MASLD risk were not modified by genetic susceptibility to MASLD (*p* interaction = 0.42). A significant inverse association was found in medium PRS subgroup (Table S6). When assessing the joint association of the EAT-Lancet diet index and PRS with MASLD risk, groups with higher EAT-Lancet diet index had lower MASLD risk, and participants with low PRS and the highest EAT-Lancet diet index had the lowest MASLD risk (Fig. S4).

The analysis of repeated metabolic biomarker assessments over 1–2 years (Phase 2; n = 10,275) revealed that the metabolite signature score, derived from selected metabolites, was significantly correlated with the EAT-Lancet diet at baseline (Pearson r = 0.24; *p <*0.0001) (Fig. S5). Moreover, the metabolic signatures at Phase 2 were inversely associated with incident MASLD at a similar magnitude. The HRs (95% CI) for MASLD across quartiles 2-4 of the metabolite signature score were 0.51 (0.30–0.85), 0.36 (0.19–0.66), and 0.52 (0.28–0.94), respectively (Table S7).

In the sensitivity analysis, we found that the association between the overall EAT-Lancet diet score and the risk of developing MASLD was only minimally influenced by the individual components (Table S8). Furthermore, when we conducted additional adjustments for liver function (Table S9, sensitivity analysis 1) and waist circumference (Table S9, sensitivity analysis 2), the associations between the EAT-Lancet diet and MASLD remained largely unchanged. Additionally, when we excluded participants with less than two dietary assessments (Table S9, sensitivity analysis 3), those with less than 2 years of follow-up (Table S9, sensitivity analysis 4), and when we expanded the definition of MASLD (Table S9, sensitivity analysis 5), the associations between the EAT-Lancet diet and MASLD were not significantly altered. In subgroup analyses, there was no evidence of any effect modification by age, sex, BMI, education level, physical activity, alcohol intake, smoking status, hypertension, nor diabetes (Fig. S6).

## Discussion

In this large prospective study, we found that a higher adherence to the EAT-Lancet diet was associated with a lower risk of MASLD, regardless of genetic susceptibility. Individuals in the uppermost quartile of the EAT-Lancet diet adherence showed a 21% reduced risk of developing MASLD compared to those in the lowest quartile. Additionally, we identified a metabolic signature tightly linked to the EAT-Lancet diet index that inversely affected MASLD risk. This association was independent of the corresponding diet index and covariates that associated with MASLD risk. Notably, six specific metabolites were identified to be strongly associated with the EAT-Lancet diet index, with five of these serving as potential intermediary biomarkers in the association between the diet index and MASLD risk.

Prior research has highlighted the EAT-Lancet diet's positive role in mitigating the risk of type 2 diabetes, atrial fibrillation, stroke, and all-cause mortality.[30], [31], [32], [33] Building on these insights, our study explores the EAT-Lancet diet's extension of protective benefits towards MASLD. This aligns with earlier findings on the advantages of plant-based diets in lowering MASLD risk. A notable investigation involving 159,222 UK Biobank participants demonstrated that individuals in the highest tertile of the overall plant-based diet index (PDI) and healthful PDI experienced a 22% and 26% decreased risk of MASLD, respectively.^10^ Similarly, a study with 963 elderly participants showed that a diet rich in plant-based foods, fiber, and low in fats was associated with a regression of MASLD over time.^34^ Supporting our outcomes, a cohort study utilizing principal component analysis to identify prudent dietary patterns (emphasizing raw and cooked vegetables, fruits, and fish) demonstrated that high adherence was associated with a 15% (95% CI 4%-25%) lower risk of developing cirrhosis.^35^ Moreover, we noted that individuals with the highest adherence to the EAT-Lancet index (Quartile 4) were healthier at baseline, potentially biasing our results as these health traits could lower disease risk independently. However, through stratified analysis and interaction tests between these health variables and the EAT-Lancet index, no significant interactions were found, confirming our findings' consistency and robustness across varied baseline health conditions.

In our study, we additionally identified a distinctive metabolic signature that serves as a reflection of adherence to the EAT-Lancet diet index. This discovery provides valuable insights into the connection between dietary patterns, potential intermediary metabolic biomarkers, and the subsequent risk of MASLD. Notably, we found that triglycerides in HDL, which serve as a biomarker for metabolic and cardiovascular risk that may signify HDL dysfunction,^36^ exhibited an inverse relationship with the EAT-Lancet diet index and a positive association with meat and added sugar intake. These triglycerides showed a positive association with MASLD incidence after adjusting for various factors. A previous study indicated a significant association between elevated HDL triglyceride levels and increased liver fat content, especially in men with type 2 diabetes and metabolic syndrome.^37^ Additionally, adhering to the EAT-Lancet diet was associated with lower levels of SFAs and higher levels of polyunsaturated fatty acids (PUFAs) and unsaturation of fatty acids. In a randomized-controlled trial involving 39 young, normal-weight participants, individuals were given a diet excessively high in SFAs from palm oil or n-6 PUFAs from sunflower oil for 7 weeks. Despite both groups experiencing similar weight gains, only those consuming saturated fats encountered a notable 40% increase in liver fat, while the polyunsaturated fat intake did not affect liver fat levels.^38^ A parallel randomized trial in people with overweight also demonstrated that overfeeding with SFAs led to a significant accumulation of liver fat, while an excess intake of PUFAs did not alter liver fat levels, despite similar weight gain.^39^ Unlike strict vegetarian diets, the EAT-Lancet diet advocates for a predominantly plant-based food intake while permitting moderate consumption of certain animal products, such as fish, which was significantly associated with high levels of omega-3 fatty acids and docosahexaenoic acid. Earlier research indicated that higher intake of freshwater fish may benefit MASLD by modulating gut microbiota and their metabolites.^40^ Moreover, our analysis suggests a connection between several amino acids and the EAT-Lancet diet index, with implications for MASLD risk that align with findings from a Mendelian randomization study.^15^ Specifically, branched-chain amino acids, such as isoleucine, leucine, and valine, were positively correlated with MASLD risk, while glycine and glutamine were associated with a reduced risk. Short-term dietary reduction of branched-chain amino acids has been shown to acutely decrease meal-induced insulin secretion and enhance postprandial insulin sensitivity in individuals with diabetes.^41^ Conversely, animal studies indicate that glutamine supplements might decelerate the progression of MASLD or non-alcoholic steatohepatitis.^42^^,^^43^

The metabolite signature we discovered, linked to adherence to the EAT-Lancet diet, provides a powerful tool for clinicians to non-invasively assess diet quality and MASLD risk.^44^ Furthermore, the association of this metabolic signature with specific dietary components, such as meat and added sugar intake, provides a basis for targeted dietary interventions. By measuring specific biomarkers, such as triglycerides in HDL and fatty acid profiles, healthcare professionals can offer personalized dietary advice aimed at preventing MASLD.

We also conducted analyses to assess whether the association between the EAT-Lancet diet and risk of MASLD could be modified by genetic predisposition to MASLD. In the subgroup analysis, we found this association to be present exclusively in participants with a medium genetic risk for MASLD. The lack of a significant link in other genetic risk groups might be due to the relatively limited number of MASLD incidences within these subsets, which was reflected by the broader 95% CIs in comparison to the medium genetic risk group. Additionally, the lack of a significant interaction may stem from the minimal percentage of genetic risk explained by the SNPs included. Given the lack of a significant interaction, we speculate that the EAT-Lancet diet's impact on MASLD incidence may be consistent across different levels of genetic risk. However, future studies with larger sample sizes and a more comprehensive genetic risk assessment are necessary to validate these findings and fully understand the interplay between diet, genetic predisposition, and risk of MASLD.

Additionally, our study employs elastic net regression modeling for the first time to construct a metabolic signature that reflects adherence to the EAT-Lancet diet. However, this study should be considered within the context of its inherent limitations. Firstly, some participants completed only one 24-hour dietary recall, which carried a risk of non-differential misclassification due to its limited capacity to capture the full spectrum of dietary variations. However, associations remained largely unchanged when we excluded individuals with only one dietary assessment. For those with two or more dietary assessments, we averaged their dietary intakes, reducing the risk of measurement error and better reflecting actual dietary habits. Secondly, although our analysis incorporated 170 metabolic biomarkers, the metabolomics platform utilized was not capable of detecting all established biomarkers related to food intake. Thirdly, it is important to note that the main analysis of this study only measured biomarkers at a single time point, which may not capture potential fluctuations in these markers during the follow-up period. However, it is worth mentioning that similar findings were observed in repeated assessments conducted over a period of 1-2 years, indicating that the levels of most metabolites remained relatively stable over time. Fourthly, the true incidence of MASLD might be potentially underestimated, with only 1,138 cases identified over an 11-year period based on hospital inpatient records and death registration. Nonetheless, it is improbable that the underdiagnosed cases of MASLD are related to specific dietary patterns. Assuming that the specificity of outcome detection is perfect and sensitivity is lower than 100% in detecting outcomes across all exposure groups, outcome misclassification is expected to minimally bias HR estimates.^45^ We also carried out a sensitivity analysis by broadening the definition of MASLD to include a more comprehensive set of criteria to address this potential limitation. However, the relatively low number of MASLD cases may lead to increased volatility in HR estimates, with potentially wider confidence intervals, thereby diminishing the precision and credibility of the study's conclusions (for example: the subgroup analysis by genetic risk and repeated metabolic biomarker assessments in the present study). Fifthly, given its observational nature, causality cannot be inferred from the associations observed in this study. Lastly, the predominantly white ethnicity of participants from the UK Biobank may restrict the applicability of our findings to a broader population.

Our findings indicate that higher adherence to the EAT-Lancet diet was associated with a reduced risk of MASLD, irrespective of genetic predispositions. Additionally, we have identified a distinct metabolite signature composed of 81 metabolites associated with the EAT-Lancet diet. These findings suggested that metabolites might mediate the diet's protective effects against MASLD. Future research is needed to confirm these results and explore the potential mechanisms connecting the EAT-Lancet diet to MASLD risk.

## Abbreviations

HR, hazard ratio; MASLD, metabolic dysfunction-associated steatotic liver disease; PDI, plant-based diet index; PRS, polygenic risk score; PUFAs, polyunsaturated fatty acids; SFAs, saturated fatty acids; SNPs, single nucleotide polymorphisms.

## Financial support

This research was supported by ZJU 100 Young Professor Project (X.T.), Rut and Arvid Wolff Memorial Foundation (X.T., 2023-02467). The funders had no role in the conduct of the study; collection, management, analysis, or interpretation of the data; preparation, review, or approval of the manuscript; or decision to submit the manuscript for publication.

## Conflict of interest

None of the authors has any potential conflict of interest.

Please refer to the accompanying ICMJE disclosure forms for further details.

## Authors’ contributions

Hanzhang Wu: Conceptualization, Methodology, Software, Formal analysis, Investigation, Writing - Original Draft, Writing - Review & Editing, Visualization; Jiahe Wei: Methodology, Formal analysis, Investigation, Writing - Review & Editing; Shuai Wang: Investigation, Writing - Review & Editing; Liangkai Chen: Investigation, Writing - Review & Editing; Jihui Zhang: Investigation, Writing - Review & Editing; Ningjian Wang: Conceptualization, Investigation, Writing - Original Draft, Writing - Review & Editing; Xiao Tan: Conceptualization, Software, Formal analysis, Data Curation, Investigation, Writing - Original Draft, Writing - Review & Editing, Supervision, Project administration, Funding acquisition.

## Data availability statement

UK Biobank resource data under application number 77740 was utilized for this research. The UK Biobank data is available on application (www.ukbiobank.ac.uk/).
