# Supplementary material for: Dietary pattern modifies the risk of MASLD through metabolomic signature
Source: JHEP Rep. 2024 Jun 10;6(8):101133. doi: 10.1016/j.jhepr.2024.101133 (PMC11286987; doi:10.1016/j.jhepr.2024.101133)
Supplement: Multimedia component 1 [file mmc1.pdf]

Dietary pattern modifies the risk of metabolic dysfunction-associated steatotic liver disease through metabolomic signature

Hanzhang Wu, Jiahe Wei, Shuai Wang, Liangkai Chen, Jihui Zhang, Ningjian Wang, Xiao Tan

Table of contents

Supplementary Methods……………………………………………………………….…..3

Fig. S1…………..……………………………..……….…………………….…..…..6

Fig. S2…………..……………………………..……….…………………….…..…..7

Fig. S3…………..……………………………..……….…………………….…..…..8

Fig. S4…………..……………………………..……….…………………….…..…..9

Fig. S5…………..……………………………..……….…………………….…..…..10

Fig. S6…………..……………………………..……….……………………….......11

Table S1…………..……………………………..……….…………………….......12

Table S2…………..……………………………..……….…………………….......15

Table S3…………..……………………………..……….…………………….......23

Table S4…………..……………………………..……….………………..……......24

Table S5…………..……………………………..……….…………..…..…..…..... 25

Table S6…………..……………………………..……….………………..…….....26

Table S7…………..……………………………..……….………………..…….....28

Table S8……….………………………………..…………….…..……..…..... 29

Table S9……….…………………………………..……….………………......30

Supplementary Methods

Study population

The dataset collected comprehensive information on participant demographics, lifestyle factors, physical activity levels, medical history, hospital records, and mortality data. This study received ethical approval from the National Information Governance Board for Health and Social Care and the National Health Service North West Multicenter Research Ethics Committee. Prior to the baseline assessment, all participants provided informed consent through electronic signature.

Dietary assessment and EAT-Lancet diet index

We calculated the EAT-Lancet diet index using the established method conceptualized by Stubbendorff et al. [1], which was used to estimate the adherence to the EAT-Lancet Commission recommendations. The index includes 14 food groups, categorized into 7 emphasized foods (vegetables, fruits, unsaturated oils, legumes, nuts, whole grains, and fish) and 7 limited foods (beef and lamb, pork, poultry, eggs, dairy, potatoes, and added sugar) (Table S1). Scoring for emphasized foods ranges from 0 (indicating the lowest adherence) to 3 (the highest adherence). Conversely, for the limited foods, this scoring pattern was reversed, with 3 representing the lowest adherence and 0 the highest (Table S1). The scores of the 14 food groups for an individual were aggregated to calculate the EAT-Lancet diet index, which has a theoretical range from 0 to 42. A higher index signifies greater adherence to the EAT-Lancet dietary patterns. Additionally, there was a significant correlation between the initial dietary assessment EAT-Lancet diet index and the averaged dietary assessment

EAT-Lancet diet index (Pearson correlation coefficient = 0.84, P <0.0001). Therefore, if participants completed dietary assessments multiple times, the study employed both the earliest dietary consumption date and the averaged dietary assessment EAT-Lancet diet index to maximize follow-up time.

Metabolomics measurement

A high-throughput nuclear magnetic resonance (NMR) metabolomics platform was used to analyze baseline plasma samples from approximately 280,000 randomly selected UK Biobank participants. Detailed protocols on sample collection and metabolomic quantification are presented elsewhere [2-4]. This simultaneously quantified 251 metabolic biomarkers (170 directly measured and 81 ratios of these). The biomarkers investigated in this study encompass multiple metabolic pathways. These include lipoprotein lipids in 14 subclasses, fatty acids and their compositions, as well as various low-molecular weight metabolites, such as amino acids, ketone bodies, and glycolysis metabolites quantified in molar concentration units. In the present study, we included a subset of 170 metabolic biomarkers (Table S2) that were directly measured for subsequent analyses. Metabolites with less than 10% missing were included, and missing data for each metabolite were imputed using half of the minimum measured value. Metabolites were highly correlated within each category (Fig. S2).

Polygenic risk score for MASLD

Based on the selected SNPs, the polygenic risk score (PRS) for MASLD was calculated as follows: $PRS = \beta 1 \times SNP1 + \beta 2 \times SNP2 + \ldots + \beta n \times SNPn$, where SNPn is the risk allele number of each SNP. A higher PRS indicates a higher genetic

predisposition to the disease. Participants were further divided into low (quintile 1), medium (quintiles 2–4), and high (quintile 5) PRS groups based on the quintiles of the PRS.

Statistical analysis

Models were adjusted for age, sex, BMI, smoking status, alcohol intake, education levels, Townsend deprivation index, total energy consumption, physical activity, individual history of the disease (cancer, cardiovascular disease, hypertension and diabetes), fasting duration, spectrometer, MASLD-PRS, first 10 principal components of ancestry, and genotype measurement batch. We also estimated the joint association of EAT-Lancet diet index and PRS with MASLD risk by creating a combined variable based on categories of PRS and EAT-Lancet diet index (12 categories). The reference group consisted of individuals with the highest risk combination, which involved having the lowest EAT-Lancet diet index and the highest PRS. In addition, we performed mediation analyses using the method proposed by Lange et al. [5] to evaluate the mediation of the association between EAT-Lancet diet and risk of MASLD by metabolite signature score or selected metabolites.

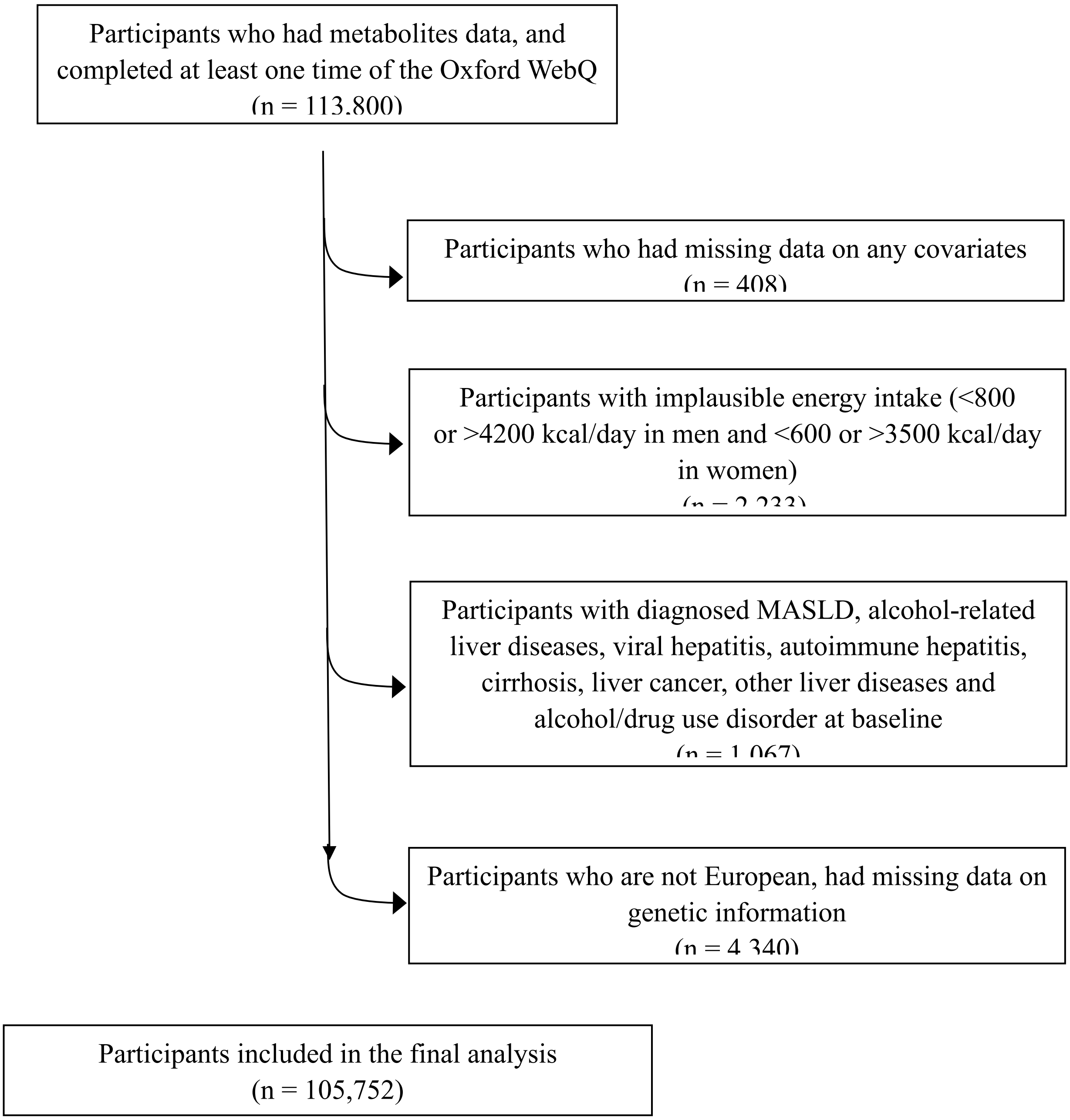

Fig. S1. Selection of study participants in the UK Biobank Cohort.

Fig. S2. Marginal correlation matrix for all the 170 metabolites included in the analysis.

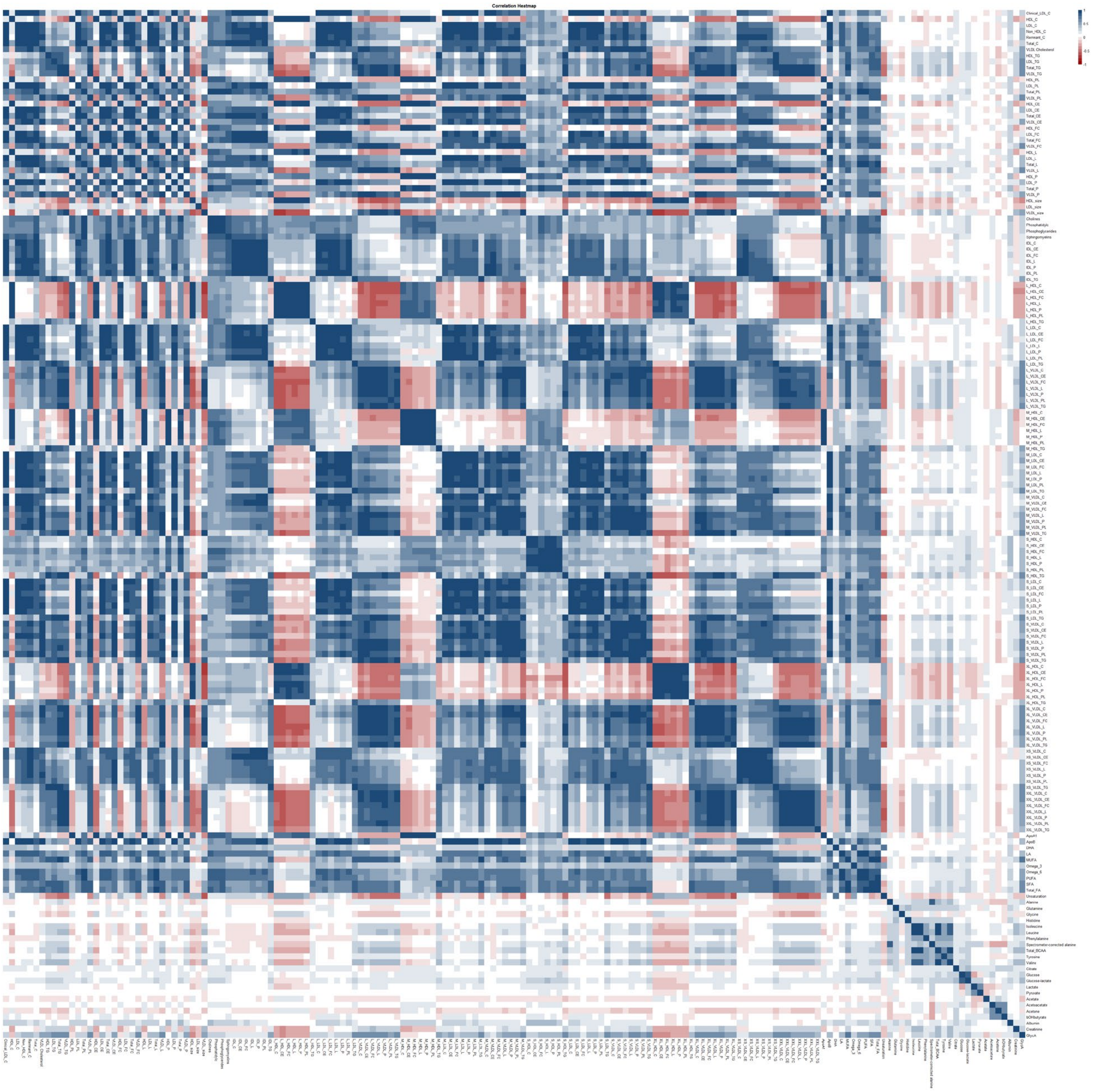

Fig. S3. Restricted cubic spline models for the association between (A) the EAT-Lancet diet index, (B) metabolic signature and risk of MASLD. The 95% CIs of the adjusted HRs are represented by the shaded are. Restricted cubic spline model is adjusted the same as the Model 2 in the Table 2. A two-tailed p <0.05 was considered statistically significant.

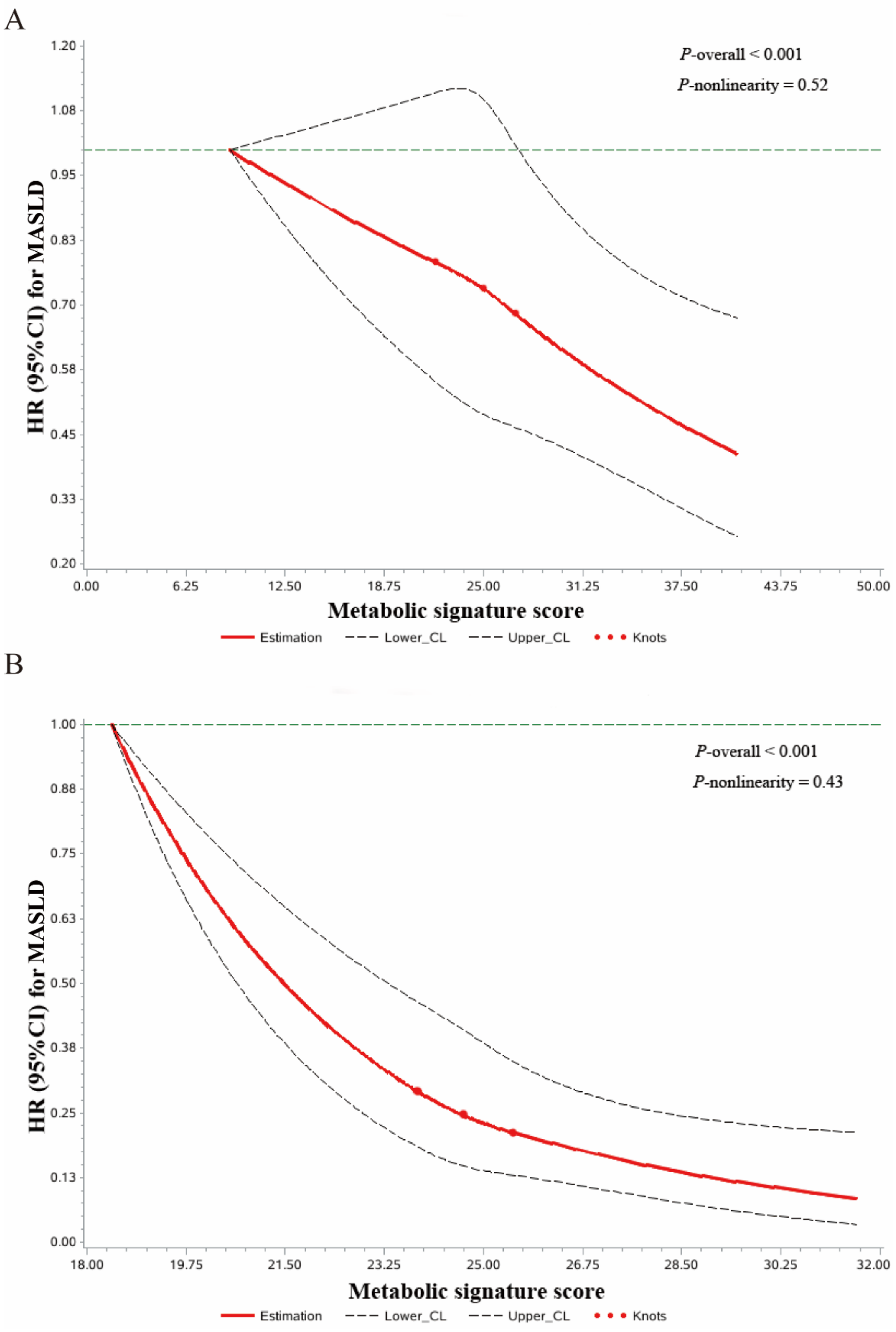

Fig. S4. Joint association of the EAT-Lancet diet index and genetic susceptibility with risk of MASLD. Adjusted for age, sex, BMI, total energy intake, smoking status, alcohol intake, educational level, Townsend deprivation index, physical activity, hypertension, diabetes, cancer, cardiovascular disease, fasting duration, spectrometer, first 10 principal components of ancestry, and genotype measurement batch.

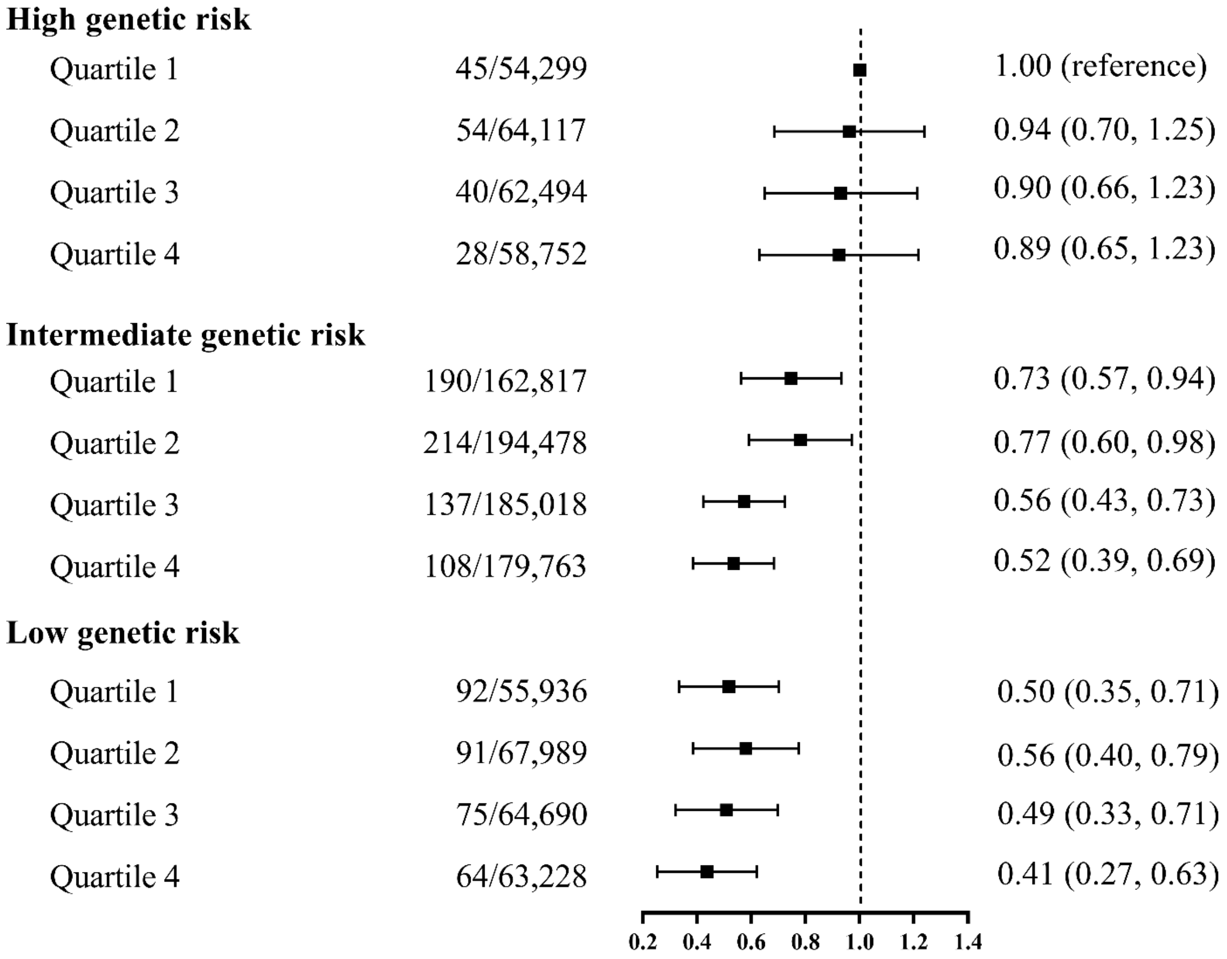

Fig. S5. Correlation between the EAT-Lancet diet and the corresponding metabolite signature score in repeated assessments.

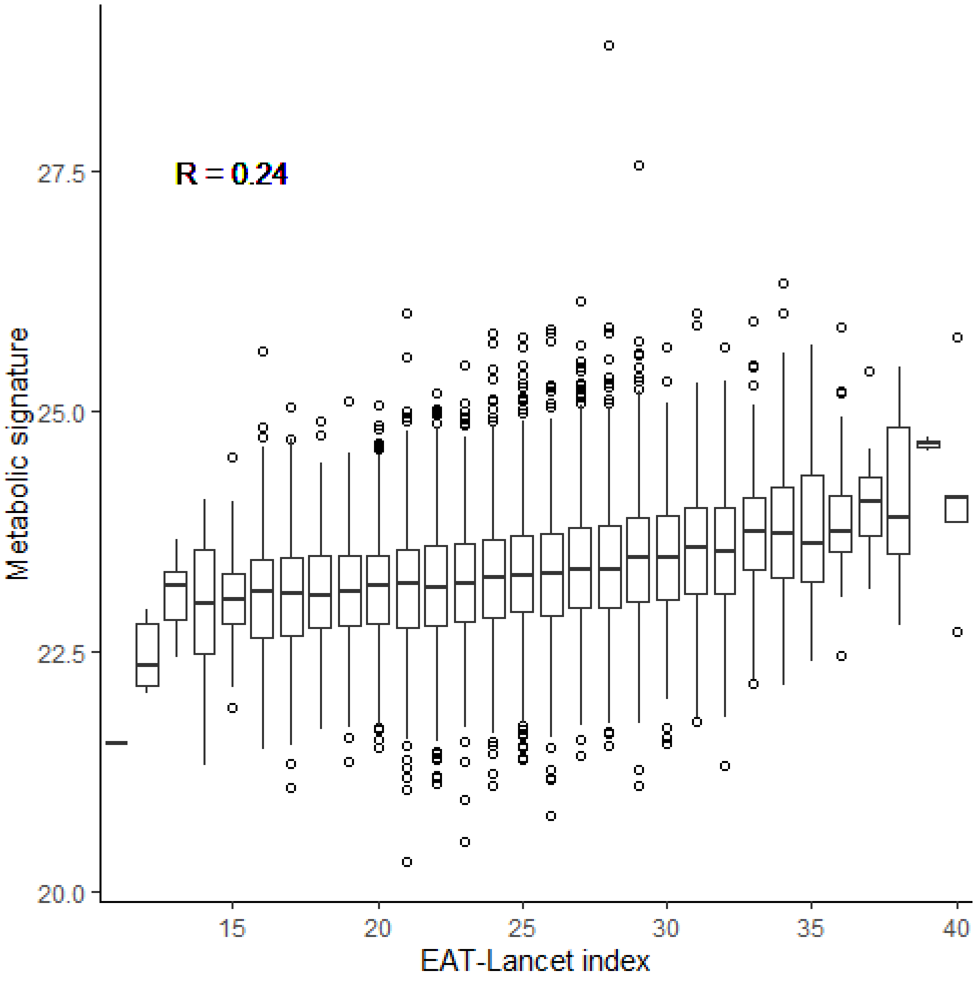

Fig. S6. Adjusted HR (95% CI) for per 1-score EAT-Lancet diet index increment and risk of MASLD stratified by potential risk factors. All models were adjusted the same as the Model 2 in the table 2. A two-tailed p <0.05 was considered statistically significant.

Risk factor	Pre 1-score increment of EAT-Lancet diet index	P for interaction
Age		0.22
< 60 years	0.95 (0.93,0.98)	
≥ 60 years	0.97 (0.94,1.00)	
Sex		0.96
Male	0.96 (0.93,0.99)	
Female	0.96 (0.93,0.99)	
BMI		0.12
< 30 kg/m²	0.96 (0.93,0.98)	
≥ 30 kg/m²	0.97 (0.94,1.00)	
Education		0.10
< College	0.97 (0.95,0.99)	
≥ College	0.94 (0.92,0.97)	
Physical activity		0.89
< 25 MET·h/wk	0.96 (0.93,0.98)	
≥ 25 MET·h/wk	0.97 (0.94,1.00)	
Alconol intake		0.10
No	0.97 (0.94,1.00)	
Yes	0.95 (0.92,0.98)	
Smokeing		0.08
Never	0.97 (0.95,1.00)	
Past	0.96 (0.93,0.99)	
Current	0.91 (0.85,0.97)	
Hypertension		0.51
No	0.96 (0.94,0.99)	
Yes	0.96 (0.93,1.00)	
Diabetes		0.83
No	0.97 (0.94,0.99)	
Yes	0.92 (0.86,0.98)	

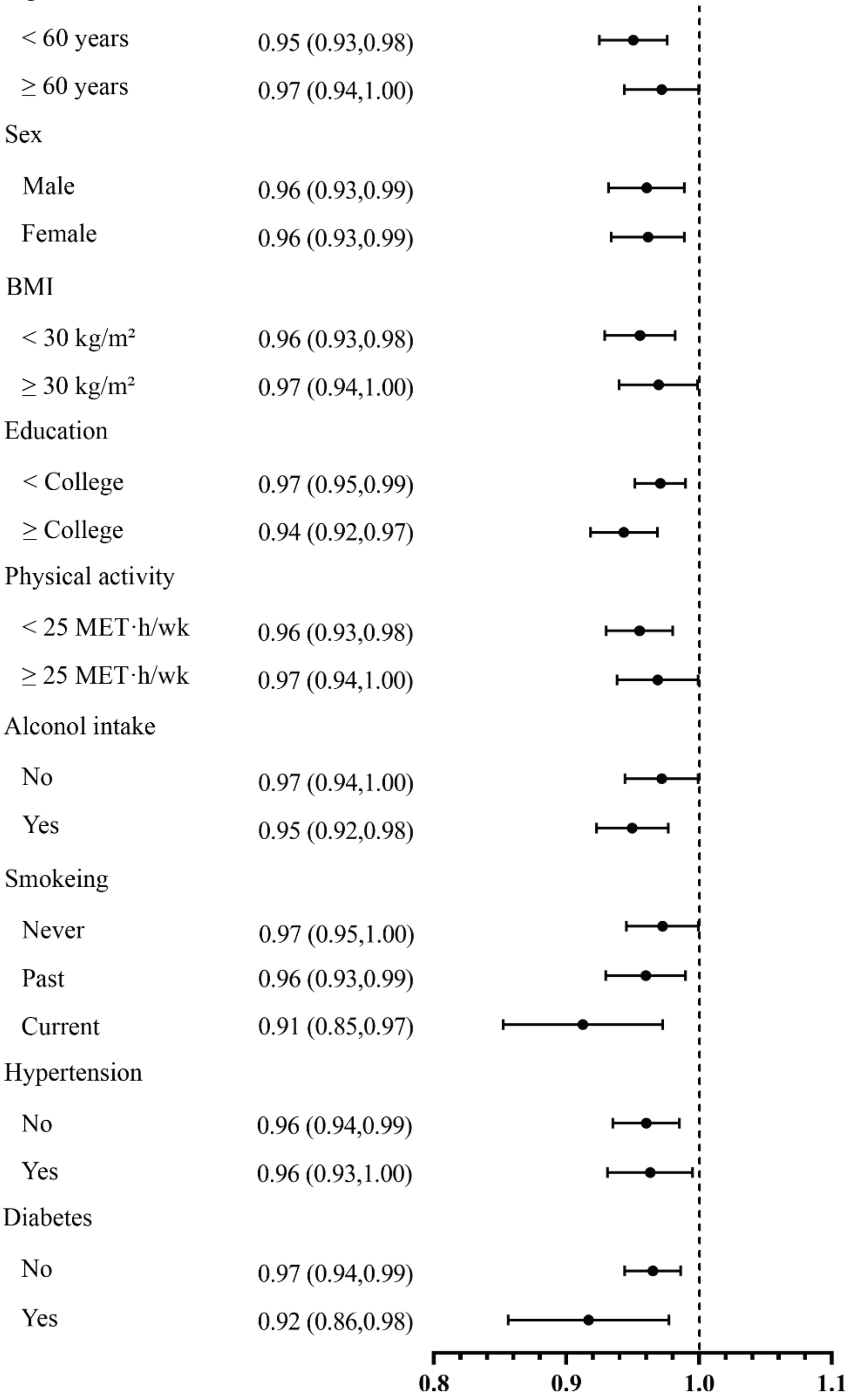

Table S1. Criteria for the EAT-Lancet diet index constructed to evaluate the EAT-Lancet diet and example of food items in the UK Biobank [a]

Food components in the EAT-Lancet diet index [b]		Target intake (reference interval)	3 points	2 points	1 point	0 points	Food items
Emphasized intake	Vegetables	300 (200–600)	>300	200–300	100–200	<100	Mixed vegetables, vegetable pieces, coleslaw, side salad, avocado, beetroot, broccoli, butternut squash, cabbage/kale, carrots, cauliflower, celery, courgette, cucumber, garlic, leeks, lettuce, mushrooms, onion, parsnip, sweet peppers, spinach, sprouts, sweetcorn, fresh tomatoes, cooked or tinned tomatoes, turnip/swede, watercress, other vegetable intake
	Fruits	200 (100–300)	>200	100–200	50–100	<50	Stewed fruit, prune, dried fruit, mixed fruit, apple, banana, berries, cherries, grapefruit, grapes, mango, melon, orange, orange-like small fruits, peach/nectarine, pear, pineapple, plum, other fruits, olives
	Unsaturated oils [c]	40 (20–80)	>40	20–40	10–20	<10	Monounsaturated fatty acids, n-3 fatty acids, n-6 fatty acids
	Legumes	75 (0–150)	>75	37.5–75	18.75–37.5	<18.75	Vegetarian sausages/burgers, tofu, quorn, other vegetarian alternative, baked beans, pulse, broad beans, green beans, peas
	Nuts	50 (0–100)	>50	25–50	12.5–25	<12.5	Salted peanuts, unsalted peanuts, salted nuts, unsalted nuts, seeds

	Whole grains	232	>232	116–232	58–116	<58	Porridge, muesli, plain cereal, bran cereal, whole-wheat cereal, oatcakes, wholemeal bread (flour type: wholemeal), whole meal pasta, brown rice, couscous, other cooked grains (such as bulgur).
	Fish	28 (0–100)	>28	14–28	7–14	<7	Tinned tuna, oily fish, breaded fish, battered fish, white fish, prawns, lobster/crab, shellfish, other fish intake
Limited intake	Beef and lamb	7 (0–14)	<7	7–14	14–28	>28	beef, lamb
	Pork	7 (0–14)	<7	7–14	14–28	>28	Sausage, pork, bacon, ham, liver, other meat intake
	Poultry	29 (0–58)	<29	29–58	58–116	>116	crumbed or deep-fried poultry, poultry
	Eggs	13 (0–25)	<13	13–25	25–50	>50	Whole eggs, omelettes or scrambled egg, eggs in sandwiches, scotch egg, other egg dishes
	Dairy	250 (0–500)	<250	250–500	500–1000	>1000	Milk, dairy smoothie, flavored milk, yogurt, ice-cream, cheesecake, milk-based pudding, other milk-based pudding, low fat hard cheese, hard cheese, soft cheese, blue cheese, low fat cheese spread, cheese spread, cottage cheese, feta cheese, mozzarella cheese, goat's cheese, other cheese
	Potatoes	50 (0–100)	<50	50–100	100–200	>200	Fried potatoes, boiled/baked potatoes, mashed potatoes, crisps (e.g., potato chips)
	Added sugar [d]	31 (0–31)	<31	31–62	62–124	>124	Added sugars and preserves

[a] Established method conceptualized by Stubbendorff et al

[b] Food components in the index are based on the EAT-Lancet diet as grams per day, with some modifications.

[c] Fat intake and quality are reflected as the consumption of unsaturated fatty acids from overall diet, since too little information about the types of oils were recorded in the 24-hour diet recall.

[d] Derived from sweet foods and beverages, and sugar added to food or beverages recorded in the 24-hour diet recall.

Table S2. Metabolic biomarkers and abbreviations included the UK Biobank

Metabolite	Type	Abbreviations	Field ID
Glucose-lactate	Glycolysis related metabolites	Glucose-lactate	20280
Spectrometer-corrected alanine	Amino acids	Spectrometer-corrected alanine	20281
Total Cholesterol	Cholesterol	Total_C	23400
Total Cholesterol Minus HDL-C	Cholesterol	Non_HDL_C	23401
Remnant Cholesterol (Non-HDL, Non-LDL -Cholesterol)	Cholesterol	Remnant_C	23402
VLDL Cholesterol	Cholesterol	VLDL Cholesterol	23403
Clinical LDL Cholesterol	Cholesterol	Clinical_LDL_C	23404
LDL Cholesterol	Cholesterol	LDL_C	23405
HDL Cholesterol	Cholesterol	HDL_C	23406
Total Triglycerides	Triglycerides	Total_TG	23407
Triglycerides in VLDL	Triglycerides	VLDL_TG	23408
Triglycerides in LDL	Triglycerides	LDL_TG	23409
Triglycerides in HDL	Triglycerides	HDL_TG	23410
Total Phospholipids in Lipoprotein Particles	Phospholipids	Total_PL	23411
Phospholipids in VLDL	Phospholipids	VLDL_PL	23412
Phospholipids in LDL	Phospholipids	LDL_PL	23413
Phospholipids in HDL	Phospholipids	HDL_PL	23414
Total Esterified Cholesterol	Cholesteryl Esters	Total_CE	23415
Cholesteryl Esters in VLDL	Cholesteryl Esters	VLDL_CE	23416
Cholesteryl Esters in LDL	Cholesteryl Esters	LDL_CE	23417
Cholesteryl Esters in HDL	Cholesteryl Esters	HDL_CE	23418

Total Free Cholesterol	Free Cholesterol	Total_FC	23419
Free Cholesterol in VLDL	Free Cholesterol	VLDL_FC	23420
Free Cholesterol in LDL	Free Cholesterol	LDL_FC	23421
Free Cholesterol in HDL	Free Cholesterol	HDL_FC	23422
Total Lipids in Lipoprotein Particles	Total Lipids	Total_L	23423
Total Lipids in VLDL	Total Lipids	VLDL_L	23424
Total Lipids in LDL	Total Lipids	LDL_L	23425
Total Lipids in HDL	Total Lipids	HDL_L	23426
Total Concentration of Lipoprotein Particles	Lipoprotein Particle Concentrations	Total_P	23427
Concentration of VLDL Particles	Lipoprotein Particle Concentrations	VLDL_P	23428
Concentration of LDL Particles	Lipoprotein Particle Concentrations	LDL_P	23429
Concentration of HDL Particles	Lipoprotein Particle Concentrations	HDL_P	23430
Average Diameter for VLDL Particles	Lipoprotein Particle Size	VLDL_size	23431
Average Diameter for LDL Particles	Lipoprotein Particle Size	LDL_size	23432
Average Diameter for HDL Particles	Lipoprotein Particle Size	HDL_size	23433
Phosphoglycerides	Other lipids	Phosphoglycerides	23434
Total Cholines	Other lipids	Cholines	23436
Phosphatidylcholines	Other lipids	Phosphatidylc	23437
Sphingomyelins	Other lipids	Sphingomyelins	23438
Apolipoprotein B	Apolipoproteins	ApoB	23439
Apolipoprotein A1	Apolipoproteins	ApoA1	23440
Total Fatty Acids	Fatty Acids	Total_FA	23442

Degree of Unsaturation	Fatty Acids	Unsaturation	23443
Omega-3 Fatty Acids	Fatty Acids	Omega_3	23444
Omega-6 Fatty Acids	Fatty Acids	Omega_6	23445
Polyunsaturated Fatty Acids	Fatty Acids	PUFA	23446
Monounsaturated Fatty Acids	Fatty Acids	MUFA	23447
Saturated Fatty Acids	Fatty Acids	SFA	23448
Linoleic Acid	Fatty Acids	LA	23449
Docosahexaenoic Acid	Fatty Acids	DHA	23450
Alanine	Amino acids	Alanine	23460
Glutamine	Amino acids	Glutamine	23461
Glycine	Amino acids	Glycine	23462
Histidine	Amino acids	Histidine	23463
Total Concentration of Branched-Chain Amino Acids (Leucine + Isoleucine + Valine)	Amino acids	Total_BCAA	23464
Isoleucine	Amino acids	Isoleucine	23465
Leucine	Amino acids	Leucine	23466
Valine	Amino acids	Valine	23467
Phenylalanine	Amino acids	Phenylalanine	23468
Tyrosine	Amino acids	Tyrosine	23469
Glucose	Glycolysis related metabolites	Glucose	23470
Lactate	Glycolysis related metabolites	Lactate	23471
Pyruvate	Glycolysis related metabolites	Pyruvate	23472

Citrate	Glycolysis related metabolites	Citrate	23473
3-Hydroxybutyrate	Ketone bodies	bOHbutyrate	23474
Acetate	Ketone bodies	Acetate	23475
Acetoacetate	Ketone bodies	Acetoacetate	23476
Acetone	Ketone bodies	Acetone	23477
Creatinine	Fluid balance	Creatinine	23478
Albumin	Fluid balance	Albumin	23479
Glycoprotein Acetyls	Fluid balance	GlycA	23480
Concentration of Chylomicrons and Extremely Large VLDL Particles	Lipoprotein subclasses	XXL_VLDL_P	23481
Total Lipids in Chylomicrons and Extremely Large VLDL	Lipoprotein subclasses	XXL_VLDL_L	23482
Phospholipids in Chylomicrons and Extremely Large VLDL	Lipoprotein subclasses	XXL_VLDL_PL	23483
Cholesterol in Chylomicrons and Extremely Large VLDL	Lipoprotein subclasses	XXL_VLDL_C	23484
Cholesteryl Esters in Chylomicrons and Extremely Large VLDL	Lipoprotein subclasses	XXL_VLDL_CE	23485
Free Cholesterol in Chylomicrons and Extremely Large VLDL	Lipoprotein subclasses	XXL_VLDL_FC	23486
Triglycerides in Chylomicrons and Extremely Large VLDL	Lipoprotein subclasses	XXL_VLDL_TG	23487
Concentration of Very Large VLDL Particles	Lipoprotein subclasses	XL_VLDL_P	23488
Total Lipids in Very Large VLDL	Lipoprotein subclasses	XL_VLDL_L	23489
Phospholipids in Very Large VLDL	Lipoprotein subclasses	XL_VLDL_PL	23490
Cholesterol in Very Large VLDL	Lipoprotein subclasses	XL_VLDL_C	23491
Cholesteryl Esters in Very Large VLDL	Lipoprotein subclasses	XL_VLDL_CE	23492
Free Cholesterol in Very Large VLDL	Lipoprotein subclasses	XL_VLDL_FC	23493
Triglycerides in Very Large VLDL	Lipoprotein subclasses	XL_VLDL_TG	23494
Concentration of Large VLDL Particles	Lipoprotein subclasses	L_VLDL_P	23495
Total Lipids in Large VLDL	Lipoprotein subclasses	L_VLDL_L	23496
Phospholipids in Large VLDL	Lipoprotein subclasses	L_VLDL_PL	23497

Cholesterol in Large VLDL	Lipoprotein subclasses	L_VLDL_C	23498
Cholesteryl Esters in Large VLDL	Lipoprotein subclasses	L_VLDL_CE	23499
Free Cholesterol in Large VLDL	Lipoprotein subclasses	L_VLDL_FC	23500
Triglycerides in Large VLDL	Lipoprotein subclasses	L_VLDL_TG	23501
Concentration of Medium VLDL Particles	Lipoprotein subclasses	M_VLDL_P	23502
Total Lipids in Medium VLDL	Lipoprotein subclasses	M_VLDL_L	23503
Phospholipids in Medium VLDL	Lipoprotein subclasses	M_VLDL_PL	23504
Cholesterol in Medium VLDL	Lipoprotein subclasses	M_VLDL_C	23505
Cholesteryl Esters in Medium VLDL	Lipoprotein subclasses	M_VLDL_CE	23506
Free Cholesterol in Medium VLDL	Lipoprotein subclasses	M_VLDL_FC	23507
Triglycerides in Medium VLDL	Lipoprotein subclasses	M_VLDL_TG	23508
Concentration of Small VLDL Particles	Lipoprotein subclasses	S_VLDL_P	23509
Total Lipids in Small VLDL	Lipoprotein subclasses	S_VLDL_L	23510
Phospholipids in Small VLDL	Lipoprotein subclasses	S_VLDL_PL	23511
Cholesterol in Small VLDL	Lipoprotein subclasses	S_VLDL_C	23512
Cholesteryl Esters in Small VLDL	Lipoprotein subclasses	S_VLDL_CE	23513
Free Cholesterol in Small VLDL	Lipoprotein subclasses	S_VLDL_FC	23514
Triglycerides in Small VLDL	Lipoprotein subclasses	S_VLDL_TG	23515
Concentration of Very Small VLDL Particles	Lipoprotein subclasses	XS_VLDL_P	23516
Total Lipids in Very Small VLDL	Lipoprotein subclasses	XS_VLDL_L	23517
Phospholipids in Very Small VLDL	Lipoprotein subclasses	XS_VLDL_PL	23518
Cholesterol in Very Small VLDL	Lipoprotein subclasses	XS_VLDL_C	23519
Cholesteryl Esters in Very Small VLDL	Lipoprotein subclasses	XS_VLDL_CE	23520
Free Cholesterol in Very Small VLDL	Lipoprotein subclasses	XS_VLDL_FC	23521
Triglycerides in Very Small VLDL	Lipoprotein subclasses	XS_VLDL_TG	23522
Concentration of IDL Particles	Lipoprotein subclasses	IDL_P	23523

Total Lipids in IDL	Lipoprotein subclasses	IDL_L	23524
Phospholipids in IDL	Lipoprotein subclasses	IDL_PL	23525
Cholesterol in IDL	Lipoprotein subclasses	IDL_C	23526
Cholesteryl Esters in IDL	Lipoprotein subclasses	IDL_CE	23527
Free Cholesterol in IDL	Lipoprotein subclasses	IDL_FC	23528
Triglycerides in IDL	Lipoprotein subclasses	IDL_TG	23529
Concentration of Large LDL Particles	Lipoprotein subclasses	L_LDL_P	23530
Total Lipids in Large LDL	Lipoprotein subclasses	L_LDL_L	23531
Phospholipids in Large LDL	Lipoprotein subclasses	L_LDL_PL	23532
Cholesterol in Large LDL	Lipoprotein subclasses	L_LDL_C	23533
Cholesteryl Esters in Large LDL	Lipoprotein subclasses	L_LDL_CE	23534
Free Cholesterol in Large LDL	Lipoprotein subclasses	L_LDL_FC	23535
Triglycerides in Large LDL	Lipoprotein subclasses	L_LDL_TG	23536
Concentration of Medium LDL Particles	Lipoprotein subclasses	M_LDL_P	23537
Total Lipids in Medium LDL	Lipoprotein subclasses	M_LDL_L	23538
Phospholipids in Medium LDL	Lipoprotein subclasses	M_LDL_PL	23539
Cholesterol in Medium LDL	Lipoprotein subclasses	M_LDL_C	23540
Cholesteryl Esters in Medium LDL	Lipoprotein subclasses	M_LDL_CE	23541
Free Cholesterol in Medium LDL	Lipoprotein subclasses	M_LDL_FC	23542
Triglycerides in Medium LDL	Lipoprotein subclasses	M_LDL_TG	23543
Concentration of Small LDL Particles	Lipoprotein subclasses	S_LDL_P	23544
Total Lipids in Small LDL	Lipoprotein subclasses	S_LDL_L	23545
Phospholipids in Small LDL	Lipoprotein subclasses	S_LDL_PL	23546
Cholesterol in Small LDL	Lipoprotein subclasses	S_LDL_C	23547
Cholesteryl Esters in Small LDL	Lipoprotein subclasses	S_LDL_CE	23548
Free Cholesterol in Small LDL	Lipoprotein subclasses	S_LDL_FC	23549

Triglycerides in Small LDL	Lipoprotein subclasses	S_LDL_TG	23550
Concentration of Very Large HDL Particles	Lipoprotein subclasses	XL_HDL_P	23551
Total Lipids in Very Large HDL	Lipoprotein subclasses	XL_HDL_L	23552
Phospholipids in Very Large HDL	Lipoprotein subclasses	XL_HDL_PL	23553
Cholesterol in Very Large HDL	Lipoprotein subclasses	XL_HDL_C	23554
Cholesteryl Esters in Very Large HDL	Lipoprotein subclasses	XL_HDL_CE	23555
Free Cholesterol in Very Large HDL	Lipoprotein subclasses	XL_HDL_FC	23556
Triglycerides in Very Large HDL	Lipoprotein subclasses	XL_HDL_TG	23557
Concentration of Large HDL Particles	Lipoprotein subclasses	L_HDL_P	23558
Total Lipids in Large HDL	Lipoprotein subclasses	L_HDL_L	23559
Phospholipids in Large HDL	Lipoprotein subclasses	L_HDL_PL	23560
Cholesterol in Large HDL	Lipoprotein subclasses	L_HDL_C	23561
Cholesteryl Esters in Large HDL	Lipoprotein subclasses	L_HDL_CE	23562
Free Cholesterol in Large HDL	Lipoprotein subclasses	L_HDL_FC	23563
Triglycerides in Large HDL	Lipoprotein subclasses	L_HDL_TG	23564
Concentration of Medium HDL Particles	Lipoprotein subclasses	M_HDL_P	23565
Total Lipids in Medium HDL	Lipoprotein subclasses	M_HDL_L	23566
Phospholipids in Medium HDL	Lipoprotein subclasses	M_HDL_PL	23567
Cholesterol in Medium HDL	Lipoprotein subclasses	M_HDL_C	23568
Cholesteryl Esters in Medium HDL	Lipoprotein subclasses	M_HDL_CE	23569
Free Cholesterol in Medium HDL	Lipoprotein subclasses	M_HDL_FC	23570
Triglycerides in Medium HDL	Lipoprotein subclasses	M_HDL_TG	23571
Concentration of Small HDL Particles	Lipoprotein subclasses	S_HDL_P	23572
Total Lipids in Small HDL	Lipoprotein subclasses	S_HDL_L	23573
Phospholipids in Small HDL	Lipoprotein subclasses	S_HDL_PL	23574
Cholesterol in Small HDL	Lipoprotein subclasses	S_HDL_C	23575

Cholesteryl Esters in Small HDL	Lipoprotein subclasses	S_HDL_CE	23576
Free Cholesterol in Small HDL	Lipoprotein subclasses	S_HDL_FC	23577
Triglycerides in Small HDL	Lipoprotein subclasses	S_HDL_TG	23578

Table S3. Definitions of MASLD and other liver diseases

	ICD-9	ICD-10
Nonalcoholic fatty liver disease	5718	K760
Nonalcoholic steatohepatitis		K758
Alcohol related liver disease	571, 5710, 5711, 5712, 5713	K70
Viral hepatitis	70	B16, B17, B18, B19
Autoimmune liver disease	5716, 5761	K830, K743, K754
Cirrhosis	27103, 4562, 5712, 5715, 57150, 57151, 57152, 57158, 57159, 5716	I850, I859, K703, K704, K717, K721, K744, K745, K746, K766, K767
Other liver diseases [a]	2750, 2751, 2776, 4530, 5714, 5716, 5715, 4561, 54621, 4560, 45620, 7895, 5722, 5724, 5723, 1550, 152,	E831, E830, E880, I820, K765, K739, K732, K744, K745, C220, C229
Alcohol/drug use disorder	303, 3050, 291, 3575, 4255, 5353, 9801, 9809, 3051, 3052, 3053, 3054, 3055, 3056, 3057, 3058, 3059	F10, E244, G621, I426, K292, G312, G721, K852, K860, T510, T519,F11, F12, F13, F14, F16, F18, F19

[a] Other liver diseases including liver diseases as well as liver cancer mentioned in ICD-9 and ICD-10 coding.

Table S4. SNPs used for creating the MASLD-PRS

Gene Locus	Chr	SNP	Risk allele	Reference allele	Beta
PNPLA3	22	rs738409	G	C	0.594
TM6SF2	19	rs58542926	T	C	0.166
MBOAT7	19	rs641738	T	C	0.073
GCKR	2	rs1260326	T	C	0.271
HSD17B13	4	rs72613567	T	TA	0.216

Abbreviations: Chr, chromosome; MASLD-PRS, metabolic dysfunction-associated steatotic liver disease-polygenic risk score; SNP, single nucleotide polymorphism.

Table S5. Association between MASLD-PRS and risk of MASLD [a]

	low MASLD-PRS	medium MASLD-PRS	high MASLD-PRS	per SD increment of MASLD-PRS
Median (min, max)	0.34 (0.00, 0.50)	0.78 (0.51, 1.23)	1.44 (1.23, 2.64)	
No. of MASLD	167	649	322	
Person years	239,662	722,076	251,844	
Incidence per 1000 PYs	0.70	0.90	1.28	
Model 1	1.00 (reference)	1.31 (1.11, 1.56)	1.88 (1.56, 2.27)	1.25 (1.18, 1.32)
Model 2	1.00 (reference)	1.33 (1.12, 1.58)	1.90 (1.57, 2.30)	1.25 (1.18, 1.32)

Abbreviations: BMI; body mass index; MASLD-PRS, metabolic dysfunction-associated steatotic liver disease-polygenic risk score; PYs, person-years.

a Values are hazard ratios (95% confidence interval) (all such values).

[b] *P* for trend was calculated across quartiles using multivariable Cox regression models.

Model 1 was adjusted for age, sex, and BMI.

Model 2 was additionally adjusted for total energy intake, smoking status, alcohol intake, educational level, Townsend deprivation index, physical activity, hypertension, diabetes, cancer, cardiovascular disease, fasting duration, spectrometer, first 10 principal components of ancestry, and genotype measurement batch.

Table S6. Subgroup analysis of the association between EAT-Lancet diet index and the risk of MASLD by genetic risk [a]

	Categories of the EAT-Lancet diet index				*P* for trend [c]
	Quartile 1	Quartile 2	Quartile 3	Quartile 4	
**Low MASLD-PRS**					
No. of MASLD	45	54	40	28	
Person years	54,299	64,117	62,494	58,752	
Incidence per 1000 PYs	0.83	0.84	0.64	0.48	
Model 1	1.00 (reference)	1.09 (0.73, 1.62) [b]	0.93 (0.60, 1.42)	0.76 (0.47, 1.22)	0.21
Model 2	1.00 (reference)	1.16 (0.77, 1.74)	0.99 (0.63, 1.54)	0.81 (0.49, 1.33)	0.46
**Medium MASLD-PRS**					
No. of MASLD	190	214	137	108	
Person years	162,817	194,478	185,018	179,763	
Incidence per 1000 PYs	1.17	1.10	0.74	0.60	
Model 1	1.00 (reference)	1.02 (0.84, 1.24)	0.73 (0.59, 0.92)	0.69 (0.54, 0.88)	<0.001
Model 2	1.00 (reference)	1.05 (0.86, 1.28)	0.77 (0.61, 0.96)	0.72 (0.56, 0.92)	<0.001
**High MASLD-PRS**					
No. of MASLD	92	91	75	64	
Person years	55,936	67,989	64,690	63,228	
Incidence per 1000 PYs	1.64	1.34	1.16	1.01	
Model 1	1.00 (reference)	0.89 (0.67, 1.19)	0.84 (0.61, 1.14)	0.81 (0.59, 1.12)	0.17
Model 2	1.00 (reference)	0.92 (0.69, 1.23)	0.88 (0.65, 1.20)	0.88 (0.63, 1.23)	0.38

Abbreviations: BMI, body mass index; MASLD-PRS, metabolic dysfunction-associated steatotic liver disease-polygenic risk score; PYs, person-years.

[a] Obtained by using multivariable Cox regression model.

[b] Hazard ratios (95% confidence interval) (all such values).

[c] P for trend was calculated across quartiles using multivariable Cox regression models. A two-tailed $p < 0.05$ was considered statistically significant.
Model 1 was adjusted for age, sex, and BMI.
Model 2 was additionally adjusted for total energy intake, smoking status, alcohol intake, educational level, Townsend deprivation index, physical activity, hypertension, diabetes, cancer, cardiovascular disease, fasting duration, spectrometer, MASLD-PRS, first 10 principal components of ancestry, and genotype measurement batch.

Table S7. Association between repeated assessments of metabolic signature with MASLD risk [a]

	Categories of the EAT-Lancet diet index				*P* for trend [c]
	Quartile 1	Quartile 2	Quartile 3	Quartile 4	
**Metabolic signature**					
No. of MASLD	49	21	13	16	
Person years	28,274	28,583	28,585	28,448	
Incidence per 1000 PYs	1.73	0.73	0.45	0.56	
Model 1	1.00 (reference)	0.48 (0.29, 0.81)	0.34 (0.18, 0.63)	0.49 (0.27, 0.87)	<0.001
Model 2	1.00 (reference)	0.51 (0.30, 0.85)	0.36 (0.19, 0.66)	0.52 (0.28, 0.94)	<0.01
Model 2 + mutual adjustment	1.00 (reference)	0.51 (0.30, 0.86)	0.36 (0.19, 0.67)	0.53 (0.29, 0.98)	<0.01

Abbreviations: BMI, body mass index; MASLD-PRS, metabolic dysfunction-associated steatotic liver disease-polygenic risk score; PYs, person-years.

[a] Obtained by using multivariable Cox regression model.

[b] Hazard ratios (95% confidence interval) (all such values).

[c] P for trend was calculated across quartiles using multivariable Cox regression models. A two-tailed p <0.05 was considered statistically significant.

Model 1 was adjusted for age, sex, and BMI.

Model 2 was additionally adjusted for total energy intake, smoking status, alcohol intake, educational level, Townsend deprivation index, physical activity, hypertension, diabetes, cancer, cardiovascular disease, fasting duration, spectrometer, MASLD-PRS, first 10 principal components of ancestry, and genotype measurement batch.

Model 2 + mutual adjustment was additionally included both EAT-Lancet diet index and the metabolic signature of EAT-Lancet diet simultaneously in the model2 to examine association independence.

Table S8. Associations between the recomputed EAT-Lancet diet score and risk of MASLD in the UK Biobank

Excluded component	HR (95 % CI) [a]	P **value**
Vegetables	0.912 (0.856, 0.971)	<0.01
Fruits	0.939 (0.890, 0.991)	0.02
Unsaturated oils	0.952 (0.912, 0.994)	0.03
Legumes	0.962 (0.928, 0.996)	0.03
Nuts	0.971 (0.943, 0.999)	0.04
Whole grains	0.977 (0.955, 0.999)	0.04
Fish	0.980 (0.960, 0.999)	0.04
Beef and lamb	0.982 (0.933, 0.998)	0.03
Pork	0.984 (0.971, 0.998)	0.02
Poultry	0.987 (0.976, 0.998)	0.02
Eggs	0.990 (0.981, 0.999)	0.03
Dairy	0.992 (0.985, 0.999)	0.03
Potatoes	0.993 (0.988, 0.999)	0.03
Added sugar	0.995 (0.990, 0.999)	0.03

Multivariable Cox models adjusted for age, sex, BMI, total energy intake, smoking status, alcohol intake, educational level, Townsend deprivation index, physical activity, hypertension, diabetes, cancer, cardiovascular disease, fasting duration, spectrometer, MASLD-PRS, first 10 principal components of ancestry, and genotype measurement batch.

[a] Hazard ratios (95 % confidence intervals) for per 4-point increase (about one standard deviation). A two-tailed p <0.05 was considered statistically significant.

Table S9. Sensitivity analyses for associations between EAT-Lancet diet index and risk of MASLD [a]

	Categories of the EAT-Lancet diet index				*P* for trend [c]
	Quartile 1	Quartile 2	Quartile 3	Quartile 4	
**EAT-Lancet diet index**					
Sensitivity analysis 1	1.00 (reference)	1.01 (0.86, 1.18) [b]	0.84 (0.70, 1.01)	0.82 (0.67, 0.99)	0.01
Sensitivity analysis 2	1.00 (reference)	1.04 (0.89, 1.21)	0.84 (0.71, 1.00)	0.79 (0.66, 0.95)	<0.01
Sensitivity analysis 3	1.00 (reference)	0.91 (0.74, 1.10)	0.71 (0.57, 0.89)	0.68 (0.53, 0.87)	<0.001
Sensitivity analysis 4	1.00 (reference)	1.01 (0.86, 1.18)	0.82 (0.69, 0.97)	0.76 (0.62, 0.91)	<0.001
Sensitivity analysis 5	1.00 (reference)	1.00 (0.88, 1.13)	0.85 (0.74, 0.97)	0.83 (0.72, 0.95)	<0.01

Abbreviations: MASLD, metabolic dysfunction-associated steatotic liver disease.

[a] The initial model was same as the model 2 in Table2.

[b] Hazard ratios (95% confidence interval) (all such values).

[c] P for trend was calculated across quartiles using multivariable Cox regression models. A two-tailed p <0.05 was considered statistically significant.

Sensitivity analysis 1: further adjusted for baseline albumin, alanine aminotransferase, and gamma-glutamyltransferase.

Sensitivity analysis 2: further adjusted for waist circumference.

Sensitivity analysis 3: excluded participants with less than twice dietary assessment, n = 64,373.

Sensitivity analysis 4: excluded participants with less than 2 years of follow-up, n = 105,660.

Sensitivity analysis 5: expanded the definition of MASLD, n = 105,670, case = 1,724.

Reference

[1] Stubbendorff A, Sonestedt E, Ramne S, Drake I, Hallstrom E, Ericson U. Development of an EAT-Lancet index and its relation to mortality in a Swedish population. Am J Clin Nutr 2022;115:705-716.
[2] Wurtz P, Kangas AJ, Soininen P, Lawlor DA, Davey Smith G, Ala-Korpela M. Quantitative Serum Nuclear Magnetic Resonance Metabolomics in Large-Scale Epidemiology: A Primer on -Omic Technologies. Am J Epidemiol 2017;186:1084-1096.
[3] Soininen P, Kangas AJ, Wurtz P, Suna T, Ala-Korpela M. Quantitative serum nuclear magnetic resonance metabolomics in cardiovascular epidemiology and genetics. Circ Cardiovasc Genet 2015;8:192-206.
[4] Soininen P, Kangas AJ, Wurtz P, Tukiainen T, Tynkkynen T, Laatikainen R, et al. High-throughput serum NMR metabonomics for cost-effective holistic studies on systemic metabolism. Analyst 2009;134:1781-1785.
[5] Lange T, Vansteelandt S, Bekaert M. A simple unified approach for estimating natural direct and indirect effects. Am J Epidemiol 2012;176:190-195.
