# Supplementary material for: Dietary pattern modifies the risk of MASLD through metabolomic signature
Source: JHEP Rep. 2024 Jun 10;6(8):101133. doi: 10.1016/j.jhepr.2024.101133 (PMC11286987; doi:10.1016/j.jhepr.2024.101133)
Supplement: Multimedia component 4 [file mmc4.pdf]

Dietary pattern modifies the risk of MASLD through metabolomic signature

Hanzhang Wu[1,2,†], **Jiahe Wei**[1,2,†], Shuai Wang[1,2], Liangkai Chen[3], Jihui Zhang[4], Ningjian Wang[5,*], Xiao Tan[1,2,6,*]

JHEP Reports **2024**. vol. 6 | 1–10

Check for updates

Background & Aims: The EAT-Lancet Commission in 2019 advocated a plant-centric diet for health and environmental benefits, but its relation to metabolic dysfunction-associated steatotic liver disease (MASLD) is unclear. We aimed to discover the metabolite profile linked to the EAT-Lancet diet and its association with MASLD risk, considering genetic predisposition.

Methods: We analyzed data from 105,752 UK Biobank participants with detailed dietary and metabolomic information. We constructed an EAT-Lancet diet index and derived a corresponding metabolomic signature through elastic net regression. A weighted polygenic risk score for MASLD was computed from associated risk variants. The Cox proportional hazards model was employed to estimate hazard ratios (HRs) and 95% CIs for the risk of MASLD (defined as hospital admission or death).

Results: During a median follow-up period of 11.6 years, 1,138 cases of MASLD were documented. Participants in the highest group for the EAT-Lancet diet index had a multivariable HR of 0.79 (95% CI 0.66–0.95) for MASLD compared to the lowest group. The diet's impact was unaffected by genetic predisposition to MASLD (p = 0.42). Moreover, a robust correlation was found between the metabolomic signature and the EAT-Lancet diet index (Pearson r = 0.29; p <0.0001). Participants in the highest group for the metabolomic signature had a multivariable HR of 0.46 (95% CI 0.37–0.58) for MASLD, in comparison to those in the lowest group.

Conclusions: Higher intake of the EAT-Lancet diet and its associated metabolite signature are both linked to a reduced risk of MASLD, independently of traditional risk factors.

© 2024 The Author(s). Published by Elsevier B.V. on behalf of European Association for the Study of the Liver (EASL). This is an open access article under the CC BY license (http://creativecommons.org/licenses/by/4.0/).

Introduction

Over the past 40 years, metabolic dysfunction-associated steatotic liver disease (MASLD) has become the most widespread chronic liver disorder worldwide,[1] not only standing as the predominant liver disease globally but also as the principal contributor to liver-related morbidity and mortality.[2,3] The global prevalence of MASLD is estimated to be 32.4%.[4] Despite the lack of approved therapies or specific pharmacological treatments for MASLD, adopting healthy lifestyles, such as dietary modifications and regular physical activity, is highly recommended as a preventive strategy against its onset.[5–7]

Several studies have explored how different diets affect MASLD.[8–11] The EAT-Lancet diet was proposed in 2019 by the EAT-Lancet Commission, which is designed as a universal dietary guideline aimed at benefiting human health and environmental sustainability.[12] It emphasizes whole grains, vegetables, fruits, legumes, and nuts, while limiting meat, sugar, and saturated fats. Unlike previous plant-based diets that completely exclude animal source foods,[10] the EAT-Lancet diet includes healthy animal foods like dairy and fish, offering a balanced approach. This flexibility may make it a more feasible and appealing option for individuals who prefer to include animal products in their diet. However, there is currently insufficient evidence regarding the specific impact of the EAT-Lancet diet on MASLD.

Moreover, diet can influence disease risk by modulating metabolic pathways and homeostasis.[13] In recent years, high-throughput metabolomic profiling has allowed for the quantitative analysis of biomarkers across multiple biological pathways, assessing genetic, environmental, and pathological changes during disease development in targeted tissues or biological fluids. Several prior studies have conducted metabolomic analyses of plasma to gain a comprehensive understanding of the alterations across various metabolic pathways in patients with MASLD.[14–16] These studies have uncovered metabolic signatures spanning the spectrum of MASLD and reveal that elevated levels of circulating triglycerides and an increase in large HDL particles, as well as a deficiency in polyunsaturated fatty acid content, may be an important risk factor for incident MASLD. This advance in metabolomic

* Corresponding authors. Addresses: Department of Big Data in Health Science, Zhejiang University School of Public Health and Sir Run Run Shaw Hospital, Zhejiang University School of Medicine, Hangzhou, China. (X. Tan), or Institute and Department of Endocrinology and Metabolism, Shanghai Ninth People's Hospital, Shanghai Jiao Tong University School of Medicine, Shanghai, China. (N. Wang).
E-mail addresses: xiao.tan@zju.edu.cn (X. Tan), wnj486@126.com (N. Wang).
† These authors contributed equally to this work.
https://doi.org/10.1016/j.jhepr.2024.101133

profiling has also enabled the objective assessment of overall adherence and metabolic responses to complex dietary patterns. However, it remains unclear whether the EAT-Lancet diet offers a protective effect against MASLD and the detailed mechanisms by which it may confer such protection through the modulation of metabolic pathways are still not fully understood.

Furthermore, the influence of genetic predisposition on the development of MASLD is widely acknowledged.[17] In recent years, numerous studies have indicated that genetic susceptibility interacts with lifestyle factors in the development of MASLD.[18,19] However, it remains unclear whether there is an interaction between the EAT-Lancet diet and genetic susceptibility regarding the risks of MASLD.

In the present study, we aimed to identify metabolic signatures indicative of adherence to the EAT-Lancet diet, and assessed whether the EAT-Lancet diet and its metabolic signatures were associated with subsequent MASLD. Additionally, we explored whether genetic predisposition could influence these associations.

Patients and methods

Study population

The UK Biobank is an ongoing population-based cohort study established between 2006 and 2010.[20] It recruited over 500,000 general participants, aged 39 to 72 years, from 22 assessment centers across England, Scotland, and Wales. Detailed information on the study population of the UK Biobank study is presented in the Supplementary Methods.

In this particular study, we included 113,800 participants who had metabolite data, and completed the web-based 24-h dietary assessments (the Oxford WebQ) at least once. For the analysis of metabolite signature with incident MASLD, we excluded participants who had missing data on any covariates (n = 408) or those with implausible energy intake (<800 or >4,200 kcal/day in men and <600 or >3,500 kcal/day in women, n = 2,233). Additionally, participants with MASLD, alcohol-related liver diseases, viral hepatitis, autoimmune hepatitis, cirrhosis, liver cancer, other liver diseases, and alcohol/drug use disorder at baseline (n = 1,067) were also excluded. Considering that single-nucleotide polymorphisms (SNPs) are significantly associated with MASLD in participants of European descent, we ultimately excluded those who were not of European descent or had missing data on genetic information (n = 4,340). Consequently, our final study comprised 105,752 participants (Fig. S1).

Dietary assessment and EAT-Lancet diet index

In the UK Biobank, dietary information was gathered using the Oxford WebQ, a web-based tool for 24-hour recalls, designed specifically for large-scale population studies. Participants were instructed to provide the consumption of over 200 food types and more than 30 drink varieties for one specific day at a time.[21] To ensure a comprehensive dietary assessment covering various seasons, the same questionnaire was sent to all participants on four distinct occasions between February 2011 and April 2012. The Oxford WebQ has been validated through comparison with an interviewer-administered 24-hour recall and biomarkers.[22,23] Our previous study demonstrated the method for calculating the average daily intake of various food items (g/day).[24] Details on how the EAT-Lancet diet index was calculated are provided in the Supplementary Methods.

Metabolomics measurement

All metabolite values underwent a rank-based inverse normal transformation, which consists of a two-step process. First, the observations were transformed to the probability scale using the empirical cumulative distribution function. Then, the transformed observations were converted to Z-scores on the real number line using the probit function. Participants were randomly assigned to either the training set or the testing set (5 to 5 ratio) to identify metabolite signatures that are correlated with the EAT-Lancet diet index. In the training set, the elastic net model within a tenfold cross-validation framework was used to regress the EAT-Lancet diet index on the 170 named metabolites.[25] The trained model was applied to the testing sets to calculate the metabolic signature. This signature was obtained by taking the weighted sum of the selected metabolites, where the weights were determined by the coefficients from the elastic net regression. The score in the training set was derived using a leave-one-out cross-validation approach to avoid overfitting. In order to evaluate the robustness of the chosen metabolites in the metabolite signature score, we computed correlation coefficients between the overall diet index and the corresponding metabolite signature scores in the training set, test set and the combined data. Multivariable linear regression was employed to examine the associations between selected metabolites and diet index, as well as dietary components. Prior to the regression analyses, EAT-Lancet diet index and intake of each dietary component were standardized to ensure comparability. Moreover, we also used the repeated measurements data from Phase 2 NMR of metabolic biomarkers to verify our result. Details on the information of metabolomics were provided in the Supplementary Methods.

Assessment of MASLD

The assessment of MASLD was based on hospital inpatient records and death registry data. The diagnoses were recorded according to ICD-9 and ICD-10[26] (Table S3). For this study, the updates for hospital inpatient admission linkages were as follows: 31 October 2022 in England, 31 August 2022 in Scotland, and 31 May 2022 in Wales. The person-years of follow-up for each participant in the UK Biobank were calculated starting from the date of completing the Oxford WebQ dietary assessment until the date of incident MASLD, death, loss to follow-up, or the end of the follow-up period, whichever occurred first.

Polygenic risk score for MASLD

The genetic data of the UK Biobank study underwent a detailed genotyping process, imputation, and quality control, as described in previous publications.[27] For this study, a subset of five SNPs significantly associated with MASLD in participants of European descent (rs738409, rs58542926, rs641738, rs1260326, and rs72613567)[28] (Table S4). Based on the selected SNPs, the polygenic risk score (PRS) for MASLD was calculated.

Ascertainment of covariates

Information on age, sex, educational level, and smoking status was assessed through a self-reported questionnaire at the baseline investigation. The Townsend deprivation index was derived from the postcode of residence using aggregated data on unemployment, car and homeownership, and household overcrowding.[29] Physical activity was estimated in metabolic equivalent minutes per week. BMI was calculated by dividing weight in kilograms by height in meters squared. Alcohol consumption and total energy intake were estimated using data from 24-hour dietary recalls.

Statistical analysis

Baseline characteristics of the study participants were expressed as mean (SD) and categorical variables are expressed as percentages. Cox proportional hazards regression models were employed to examine the hazard ratios (HRs) and their corresponding 95% CIs in relation to the associations between the EAT-Lancet diet index, metabolite signature score, selected metabolites, and the risk of MASLD. The proportional hazards assumption was tested by Schoenfeld residuals, and no violation of this assumption was found in our analyses. We also employed restricted cubic spline analyses with three knots (25th, 50th, and 75th) to examine the dose-response association. The *p* value for interactions were calculated by including multiplicative interaction terms of the EAT-Lancet diet index and PRS categories in the fully adjusted model.

We conducted several sensitivity analyses to ensure the robustness of our primary findings. First, to assess the impact of individual components on the association between the overall EAT-Lancet diet score and MASLD, we recalculated the EAT-Lancet diet scores by excluding each component and examined the association between the recalculated score and the risk of developing MASLD. Second, we further adjusted for liver function to account for the confounding effect of baseline liver function. Third, we further adjusted for waist circumference to minimize the confounding effect of central obesity. Fourth, we excluded participants with less than two dietary assessments to ensure the reliability of the dietary data used in our analysis. Fifth, we performed subgroup analyses to assess the potential effect modifications by age, sex, BMI, education level, physical activity, alcohol intake, smoking status, hypertension, and diabetes. Sixth, we expanded the definition of MASLD to encompass a more inclusive set of criteria, including K76.8 (other specified diseases of the liver), K76.9 (unspecified liver disease), and other unspecified liver conditions such as K74.0 (hepatic fibrosis), K74.1 (hepatic sclerosis), K74.2 (hepatic fibrosis with hepatic sclerosis), and K74.6 (other and unspecified cirrhosis of the liver). Finally, we excluded MASLD cases that occurred within the first 3 years of follow-up to assess whether the results might have been influenced by reverse causation bias.

A two-sided $p < 0.05$ was considered statistically significant and multiple testing was corrected using the Bonferroni method. All statistical analyses were conducted using SAS version 9.4 (SAS Institute Inc., Gary, NC, USA) and R software version 4.3.1.

Results

A total of 105,752 participants were enrolled in this study, and they were observed for a median duration of 11.6 years, totaling 1,213,582 person-years of follow-up. During this period, 1,138 cases of MASLD were recorded. Table 1 presents the characteristics of the participants based on categories of the EAT-Lancet diet index.

Out of the 170 metabolic biomarkers initially included in the analysis, the elastic net regression identified a metabolic signature consisting of 81 metabolites (47.6%). These metabolites

Table 1. Baseline characteristics of the study participants according to quartiles of the EAT-Lancet diet index.[a]

	Categories of the EAT-Lancet diet index				
Characteristics	Quartile 1	Quartile 2	Quartile 3	Quartile 4	*p* value[b]
EAT-Lancet diet index (min, max)	9-21	22-24	25-27	28-41	
No. of participants	23,887	28,483	27,171	26,211	
Age (years)	55.5 (8.00)	56.3 (7.88)	56.6 (7.84)	56.8 (7.70)	<0.0001
Sex (male, %)	53.6	47.2	42.7	38.3	<0.0001
Body mass index (kg/m^2)	27.8 (4.77)	27.3 (4.65)	26.8 (4.52)	26.0 (4.34)	<0.0001
Townson depretive index	-1.64 (2.85)	-1.79 (2.75)	-1.86 (2.73)	-1.76 (2.77)	<0.0001
Education level (college or higher, %)	35.0	39.1	43.5	48.8	<0.0001
PA (MET × hour/week)	38.8 (40.9)	40.2 (39.9)	42.6 (40.4)	44.8 (40.7)	<0.0001
Total energy intake (kCal/day)	2,015.7 (625.0)	2,020.9 (602.9)	2,049 (593.1)	2,108.8 (591.5)	<0.0001
Alcohol intake (g/day)	20.6 (28.2)	17.4 (24.7)	16.1 (23.1)	14.4 (21.2)	<0.0001
Smoking status (%)					<0.0001
Current smoker	11.1	7.70	6.26	5.03	
Ex-smoker	35.9	35.9	36.0	36.4	
Non-smoker	53.1	56.4	57.8	58.6	
Individual history of disease (%)					
Hypertension	25.2	24.1	22.7	21.4	<0.0001
Diabetes	3.47	3.28	3.17	2.63	<0.0001
Cardiovascular disease	1.50	1.60	1.51	1.57	0.75
Cancer	10.2	10.6	10.5	11.2	<0.01
Glucose	5.12 (1.16)	5.12 (1.16)	5.10 (1.08)	5.06 (1.02)	<0.0001
HbA1c	35.7 (6.17)	35.7 (6.17)	35.5 (5.99)	35.3 (5.69)	<0.0001
Cholesterol	5.70 (1.13)	5.70 (1.13)	5.73 (1.13)	5.72 (1.11)	0.01
ALT	24.4 (14.2)	24.4 (14.2)	22.6 (13.2)	21.7 (12.1)	<0.0001

ALT, alanine aminotransferase; MET, metabolic equivalent; MASLD, metabolic dysfunction-associated steatotic liver disease; PA, physical activity.
[a]Continuous variables are expressed as mean (SD) and categorical variables are expressed as percentages.
[b]Chi-squared was used for categorical variables and one-way analysis of variance for continuous variables. A two-tailed $p < 0.05$ was considered statistically significant.

exhibited a significant association with the EAT-Lancet diet at baseline and were robust against the effects of collinearity between metabolites. Among the metabolites in the signature, the majority were lipoprotein subclasses (n = 38), while amino acids (n = 9) and fatty acids (n = 7) were also included (Fig. 1A). The metabolite signature score, based on the selected metabolites, showed a significant correlation with the EAT-Lancet diet at baseline (Fig. 1B-D; all p <0.0001).

Metabolites that positively correlated with the EAT-Lancet diet index, such as omega-3 fatty acids, docosahexaenoic acid, cholesteryl esters in very large HDL, and cholesterol in very large HDL, were also positively associated with the intake of emphasized foods such as fish, vegetables, and fruits (Fig. 2). Conversely, metabolites that inversely correlated with the EAT-Lancet diet index, such as saturated fatty acids (SFAs), triglycerides in HDL, and phospholipids in small HDL, were found to be positively associated with the consumption of beef, pork, and added sugar. Among the 81 metabolite markers, the degree of unsaturation in fatty acids emerged as the factor most significantly associated with the EAT-Lancet diet index (Fig. 3).

Table 2 demonstrates the prospective analyses of the EAT-Lancet diet index, metabolic signature, and the risk of MASLD. In the multivariable adjusted models, the HRs (95% CIs) for MASLD incidence across quartiles 2-4 of the EAT-Lancet diet index were 1.04 (0.89–1.20), 0.84 (0.71–0.99), and 0.79 (0.66–0.95), respectively. Likewise, the metabolite signature score showed an inverse association with MASLD incidence even after adjustment for multiple variables. The HRs (95% CI) for MASLD across quartiles 2-4 of the metabolite signature score were 0.74 (0.64–0.86), 0.61 (0.52–0.73), and 0.46 (0.37–0.58) respectively. The dose-response analysis shows that the EAT-Lancet diet index and metabolite signature score had a linear association with MASLD risk (p for non-linear >0.05 for EAT-Lancet diet index and metabolite signature score) (Fig. S3). Further adjustment for the EAT-Lancet diet index and metabolic signature simultaneously only slightly attenuated the association of the metabolite signature score with MASLD risk. However, the inverse associations of the EAT-Lancet diet index with MASLD risk became noticeably weaker and no longer statistically significant after adjusting for the metabolite signature score.

Furthermore, a mediation analysis was conducted to estimate the proportion of MASLD association that could be explained by the metabolite signature score and six significant metabolic biomarkers in the volcano plot. The association between the EAT-Lancet diet index and MASLD was 34.3% (95% CI 16.6%-57.9%) mediated by the metabolic signature. Moreover, the proportion of this association between the EAT-Lancet diet index and MASLD that could be explained by these individual metabolic biomarkers ranged between 5.3% and 26.2% (Fig. 4).

Participants with high PRS had a significantly increased risk of incident MASLD compared to those with low PRS (multivariate-adjusted HR of 1.90, 95% CI 1.57–2.30) (Table S5). Stratified analyses by PRS showed that the associations between a higher EAT-Lancet diet index and lower MASLD risk were not modified by genetic susceptibility to MASLD (p interaction = 0.42). A significant inverse association was found in medium PRS subgroup (Table S6). When assessing the joint association of the EAT-Lancet diet index and PRS with MASLD risk, groups with higher EAT-Lancet diet index had lower MASLD risk, and participants with low PRS and the highest EAT-Lancet diet index had the lowest MASLD risk (Fig. S4).

The analysis of repeated metabolic biomarker assessments over 1–2 years (Phase 2; n = 10,275) revealed that the metabolite signature score, derived from selected metabolites, was significantly correlated with the EAT-Lancet diet at baseline (Pearson r = 0.24; p <0.0001) (Fig. S5). Moreover, the metabolic signatures at Phase 2 were inversely associated with incident MASLD at a similar magnitude. The HRs (95% CI) for MASLD across quartiles 2-4 of the metabolite signature score were 0.51 (0.30–0.85), 0.36 (0.19–0.66), and 0.52 (0.28–0.94), respectively (Table S7).

In the sensitivity analysis, we found that the association between the overall EAT-Lancet diet score and the risk of developing MASLD was only minimally influenced by the individual components (Table S8). Furthermore, when we conducted additional adjustments for liver function (Table S9, sensitivity analysis 1) and waist circumference (Table S9, sensitivity analysis 2), the associations between the EAT-Lancet diet and MASLD remained largely unchanged. Additionally, when we excluded participants with less than two dietary assessments (Table S9, sensitivity analysis 3), those with less than 2 years of follow-up (Table S9, sensitivity analysis 4), and when we expanded the definition of MASLD (Table S9, sensitivity analysis 5), the associations between the EAT-Lancet diet and MASLD were not significantly altered. In subgroup analyses, there was no evidence of any effect modification by age, sex, BMI, education level, physical activity, alcohol intake, smoking status, hypertension, nor diabetes (Fig. S6).

Discussion

In this large prospective study, we found that a higher adherence to the EAT-Lancet diet was associated with a lower risk of MASLD, regardless of genetic susceptibility. Individuals in the uppermost quartile of the EAT-Lancet diet adherence showed a 21% reduced risk of developing MASLD compared to those in the lowest quartile. Additionally, we identified a metabolic signature tightly linked to the EAT-Lancet diet index that inversely affected MASLD risk. This association was independent of the corresponding diet index and covariates that associated with MASLD risk. Notably, six specific metabolites were identified to be strongly associated with the EAT-Lancet diet index, with five of these serving as potential intermediary biomarkers in the association between the diet index and MASLD risk.

Prior research has highlighted the EAT-Lancet diet's positive role in mitigating the risk of type 2 diabetes, atrial fibrillation, stroke, and all-cause mortality.[30–33] Building on these insights, our study explores the EAT-Lancet diet's extension of protective benefits towards MASLD. This aligns with earlier findings on the advantages of plant-based diets in lowering MASLD risk. A notable investigation involving 159,222 UK Biobank participants demonstrated that individuals in the highest tertile of the overall plant-based diet index (PDI) and healthful PDI experienced a 22% and 26% decreased risk of MASLD, respectively.[10] Similarly, a study with 963 elderly participants showed that a diet rich in plant-based foods, fiber, and low in fats was associated with a regression of MASLD over time.[34] Supporting our outcomes, a cohort study utilizing principal component

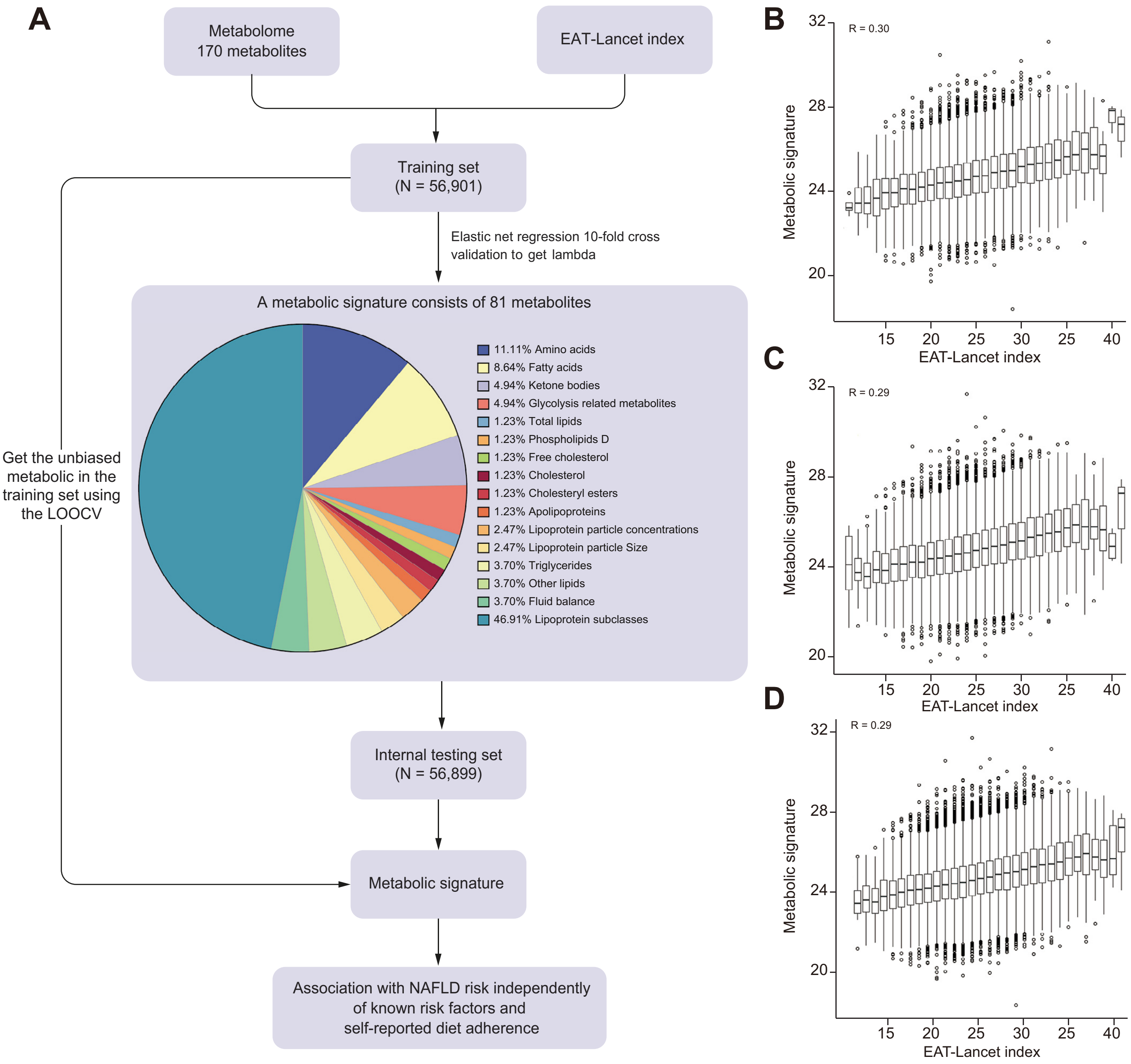

Fig. 1. The metabolic signature for adherence to the EAT-Lancet diet: flow chart for analytic approach and validation. (A) The training and testing procedures of a metabolic signature for the EAT-Lancet diet. Correlation between the EAT-Lancet diet and (B) the corresponding metabolite signature score in the training set (C) the corresponding metabolite signature score in the testing set (D) the corresponding metabolite signature score in the combined data. Pearson correlation is employed in correlation analysis. A two-tailed p <0.05 was considered statistically significant. LOOCV, leave-one-out cross-validation; MASLD, metabolic dysfunction-associated steatotic liver disease.

analysis to identify prudent dietary patterns (emphasizing raw and cooked vegetables, fruits, and fish) demonstrated that high adherence was associated with a 15% (95% CI 4%-25%) lower risk of developing cirrhosis.[35] Moreover, we noted that individuals with the highest adherence to the EAT-Lancet index (Quartile 4) were healthier at baseline, potentially biasing our results as these health traits could lower disease risk independently. However, through stratified analysis and interaction tests between these health variables and the EAT-Lancet index, no significant interactions were found, confirming our findings' consistency and robustness across varied baseline health conditions.

In our study, we additionally identified a distinctive metabolic signature that serves as a reflection of adherence to the EAT-Lancet diet index. This discovery provides valuable insights into the connection between dietary patterns, potential intermediary metabolic biomarkers, and the subsequent risk of MASLD. Notably, we found that triglycerides in HDL, which

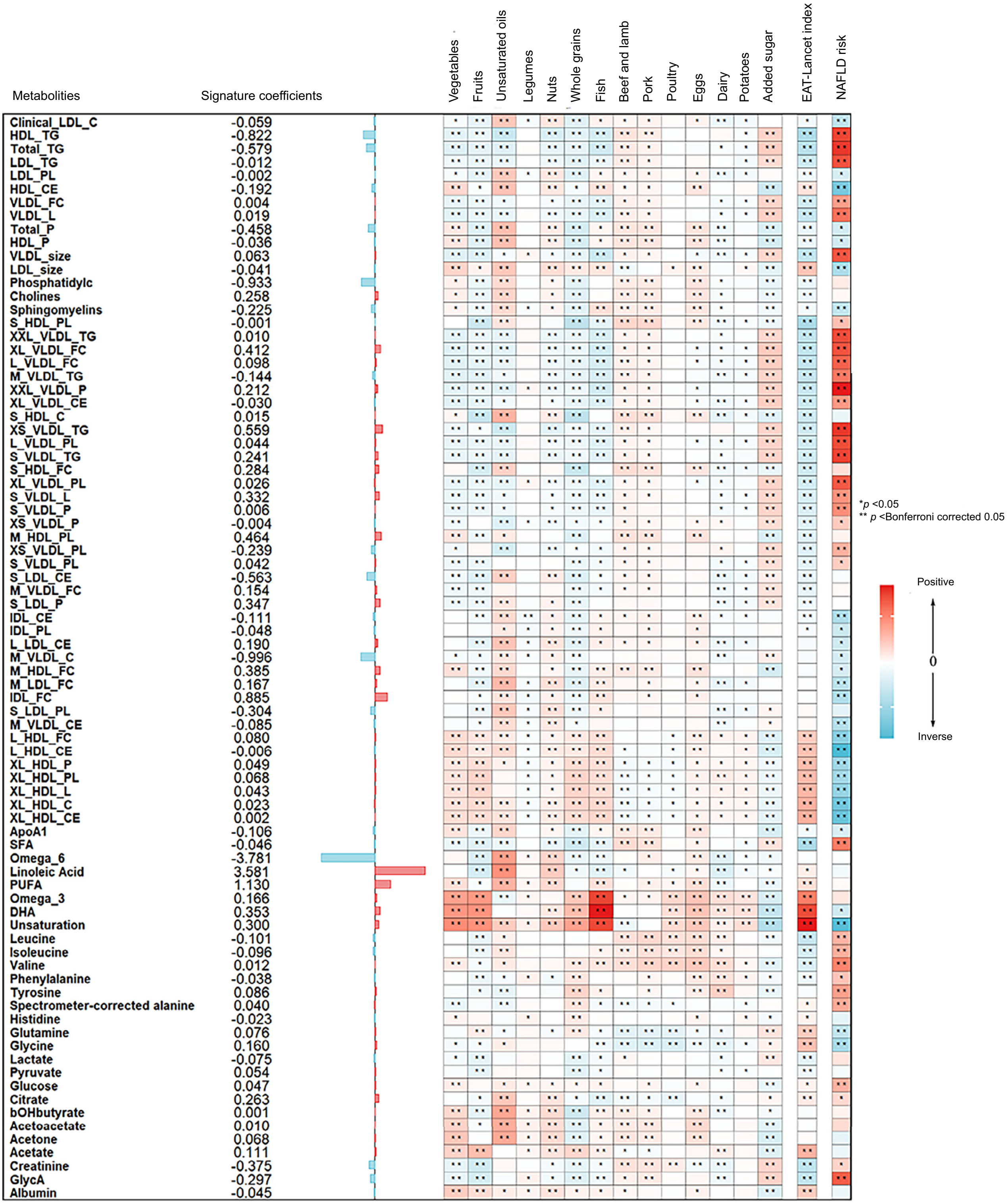

Fig. 2. Associations of the metabolic signature with the EAT-Lancet diet components, total EAT-Lancet diet index, and subsequent MASLD disease risk. Presented from left to right are the metabolites' coefficients (weights) in the signature, associations with each food component, total EAT-Lancet diet index, and subsequent MASLD risk. Coefficients for associations with food items and total EAT-Lancet diet index indicate the SD changes in metabolites per SD increment in dietary intake. Coefficients for MASLD risk indicate hazard ratio of MASLD disease risk per SD increment in metabolites. The weights in the signature were evaluated using elastic net regression. Linear regressions were used to analyze the connection between metabolites, food components, and the total EAT-Lancet diet index. Cox regressions were utilized to analyze the association between metabolites and the risk of MASLD. All models were adjusted similarly to Model 2 in Table 2. Colors indicate the direction (red for positive and blue for inverse) and strength (darker color for stronger magnitude) of the associations; asterisks denote significance (*p <0.05 and **Bonferroni corrected p <0.05; for associations with total MEDAS score and MASLD risk, we Bonferroni corrected for 81 metabolites; for associations with each food item, we Bonferroni corrected for 81 metabolites × 14 food items). MASLD, metabolic dysfunction-associated steatotic liver disease.

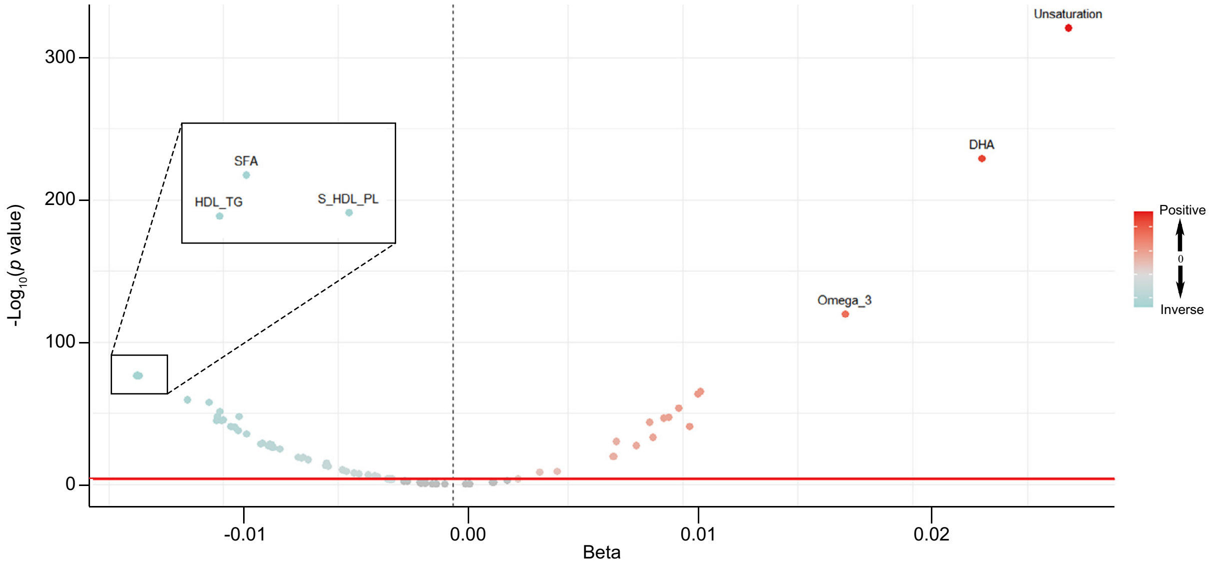

Fig. 3. Volcano plot of association between the EAT-Lancet diet index and 81 metabolites in full sample analysis. Linear regressions were adjusted in the same way as for Model 2 in Table 2. Green and red dots represent significant relationship (*p* value <0.05/81), grey dots represent non-significant relationship. MASLD, metabolic dysfunction-associated steatotic liver disease.

serve as a biomarker for metabolic and cardiovascular risk that may signify HDL dysfunction,[36] exhibited an inverse relationship with the EAT-Lancet diet index and a positive association with meat and added sugar intake. These triglycerides showed a positive association with MASLD incidence after adjusting for various factors. A previous study indicated a significant association between elevated HDL triglyceride levels and increased liver fat content, especially in men with type 2 diabetes and metabolic syndrome.[37] Additionally, adhering to the EAT-Lancet diet was associated with lower levels of SFAs and higher levels of polyunsaturated fatty acids (PUFAs) and unsaturation of fatty acids. In a randomized-controlled trial involving 39 young, normal-weight participants, individuals were given a diet excessively high in SFAs from palm oil or n-6 PUFAs from sunflower oil for 7 weeks. Despite both groups experiencing similar weight gains, only those consuming saturated fats encountered a notable 40% increase in liver fat, while the polyunsaturated fat intake did not affect liver fat levels.[38] A parallel randomized trial in people with overweight also demonstrated that overfeeding with SFAs led to a significant accumulation of liver fat, while an excess intake of PUFAs did not alter liver fat levels, despite similar weight gain.[39] Unlike

Table 2. Association of the EAT-Lancet diet index and metabolic signature with MASLD risk.[a]

	Categories of the EAT-Lancet diet index				
	**Quartile 1**	**Quartile 2**	**Quartile 3**	**Quartile 4**	***p* for trend[c]**
**EAT-Lancet diet index**					
No. of MASLD	327	359	252	200	
Person-years	273,052	326,585	312,203	301,743	
Incidence per 1,000 PYs	1.20	1.10	0.81	0.66	
Model 1	1.00 (reference)	0.99 (0.86–1.16)[b]	0.79 (0.67–0.93)	0.74 (0.62–0.88)	<0.0001
Model 2	1.00 (reference)	1.04 (0.89–1.20)	0.84 (0.71–0.99)	0.79 (0.66–0.95)	<0.001
Model 2 + mutual adjustment	1.00 (reference)	1.06 (0.91–1.23)	0.88 (0.75–1.05)	0.87 (0.73–1.05)	0.05
**Metabolic signature**					
No. of MASLD	524	306	198	110	
Person-years	306,218	304,873	303,047	299,444	
Incidence per 1,000 PYs	1.71	1.00	0.65	0.37	
Model 1	1.00 (reference)	0.73 (0.63–0.84)	0.58 (0.49–0.69)	0.43 (0.35–0.54)	<0.0001
Model 2	1.00 (reference)	0.74 (0.64–0.86)	0.61 (0.52–0.73)	0.46 (0.37–0.58)	<0.0001
Model 2 + mutual adjustment	1.00 (reference)	0.75 (0.65–0.87)	0.63 (0.52–0.75)	0.48 (0.38–0.60)	<0.0001

Model 1 was adjusted for age, sex, and BMI.
Model 2 was additionally adjusted for total energy intake, smoking status, alcohol intake, educational level, Townsend deprivation index, physical activity, hypertension, diabetes, cancer, cardiovascular disease, fasting duration, spectrometer, MASLD-PRS, first 10 principal components of ancestry, and genotype measurement batch.
Model 2 + mutual adjustment additionally included both EAT-Lancet diet index and the metabolic signature of EAT-Lancet diet simultaneously in model 2 to examine association independence.
MASLD-PRS, metabolic dysfunction-associated steatotic liver disease-polygenic risk score; PYs, person-years.
[a]Obtained by using multivariable Cox regression model.
[b]Hazard ratios (95% confidence interval) (all such values).
[c]*p* for trend was calculated across quartiles using multivariable Cox regression models. A two-tailed *p* <0.05 was considered statistically significant.

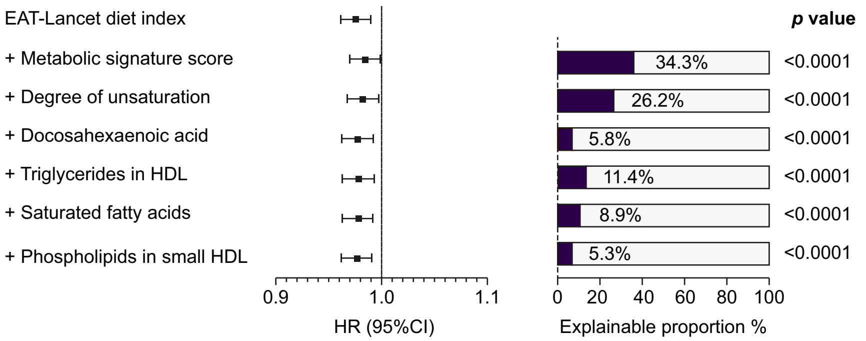

Fig. 4. Associations of the EAT-Lancet diet index with the risk of MASLD, adjusting for intermediate metabolites. All mediation analysis models were adjusted in the same way as for Model 2 in Table 2. A two-tailed *p* <0.05 was considered statistically significant. HR, hazard ratio; MASLD, metabolic dysfunction-associated steatotic liver disease.

strict vegetarian diets, the EAT-Lancet diet advocates for a predominantly plant-based food intake while permitting moderate consumption of certain animal products, such as fish, which was significantly associated with high levels of omega-3 fatty acids and docosahexaenoic acid. Earlier research indicated that higher intake of freshwater fish may benefit MASLD by modulating gut microbiota and their metabolites.[40] Moreover, our analysis suggests a connection between several amino acids and the EAT-Lancet diet index, with implications for MASLD risk that align with findings from a Mendelian randomization study.[15] Specifically, branched-chain amino acids, such as isoleucine, leucine, and valine, were positively correlated with MASLD risk, while glycine and glutamine were associated with a reduced risk. Short-term dietary reduction of branched-chain amino acids has been shown to acutely decrease meal-induced insulin secretion and enhance postprandial insulin sensitivity in individuals with diabetes.[41] Conversely, animal studies indicate that glutamine supplements might decelerate the progression of MASLD or non-alcoholic steatohepatitis.[42,43]

The metabolite signature we discovered, linked to adherence to the EAT-Lancet diet, provides a powerful tool for clinicians to non-invasively assess diet quality and MASLD risk.[44] Furthermore, the association of this metabolic signature with specific dietary components, such as meat and added sugar intake, provides a basis for targeted dietary interventions. By measuring specific biomarkers, such as triglycerides in HDL and fatty acid profiles, healthcare professionals can offer personalized dietary advice aimed at preventing MASLD.

We also conducted analyses to assess whether the association between the EAT-Lancet diet and risk of MASLD could be modified by genetic predisposition to MASLD. In the subgroup analysis, we found this association to be present exclusively in participants with a medium genetic risk for MASLD. The lack of a significant link in other genetic risk groups might be due to the relatively limited number of MASLD incidences within these subsets, which was reflected by the broader 95% CIs in comparison to the medium genetic risk group. Additionally, the lack of a significant interaction may stem from the minimal percentage of genetic risk explained by the SNPs included. Given the lack of a significant interaction, we speculate that the EAT-Lancet diet's impact on MASLD incidence may be consistent across different levels of genetic risk. However, future studies with larger sample sizes and a more comprehensive genetic risk assessment are necessary to validate these findings and fully understand the interplay between diet, genetic predisposition, and risk of MASLD.

Additionally, our study employs elastic net regression modeling for the first time to construct a metabolic signature that reflects adherence to the EAT-Lancet diet. However, this study should be considered within the context of its inherent limitations. Firstly, some participants completed only one 24-hour dietary recall, which carried a risk of non-differential misclassification due to its limited capacity to capture the full spectrum of dietary variations. However, associations remained largely unchanged when we excluded individuals with only one dietary assessment. For those with two or more dietary assessments, we averaged their dietary intakes, reducing the risk of measurement error and better reflecting actual dietary habits. Secondly, although our analysis incorporated 170 metabolic biomarkers, the metabolomics platform utilized was not capable of detecting all established biomarkers related to food intake. Thirdly, it is important to note that the main analysis of this study only measured biomarkers at a single time point, which may not capture potential fluctuations in these markers during the follow-up period. However, it is worth mentioning that similar findings were observed in repeated assessments conducted over a period of 1-2 years, indicating that the levels of most metabolites remained relatively stable over time. Fourthly, the true incidence of MASLD might be potentially underestimated, with only 1,138 cases identified over an 11-year period based on hospital inpatient records and death registration. Nonetheless, it is improbable that the underdiagnosed cases of MASLD are related to specific dietary patterns. Assuming that the specificity of outcome detection is perfect and sensitivity is lower than 100% in detecting outcomes across all exposure groups, outcome misclassification is expected to minimally bias HR estimates.[45] We also carried out a sensitivity analysis by broadening the definition of MASLD to include a more comprehensive set of criteria to address this potential limitation. However, the relatively low number of MASLD cases may lead to increased volatility in HR estimates, with potentially wider confidence intervals, thereby diminishing the precision and credibility of the study's conclusions (for example: the subgroup analysis by genetic risk and repeated metabolic biomarker assessments in

the present study). Fifthly, given its observational nature, causality cannot be inferred from the associations observed in this study. Lastly, the predominantly white ethnicity of participants from the UK Biobank may restrict the applicability of our findings to a broader population.

Our findings indicate that higher adherence to the EAT-Lancet diet was associated with a reduced risk of MASLD, irrespective of genetic predispositions. Additionally, we have identified a distinct metabolite signature composed of 81 metabolites associated with the EAT-Lancet diet. These findings suggested that metabolites might mediate the diet's protective effects against MASLD. Future research is needed to confirm these results and explore the potential mechanisms connecting the EAT-Lancet diet to MASLD risk.

Affiliations

[1]*Department of Big Data in Health Science, Zhejiang University School of Public Health, Hangzhou, China. Sir Run Run Shaw Hospital, Zhejiang University School of Medicine, Hangzhou, China;* [2]*The Key Laboratory of Intelligent Preventive Medicine of Zhejiang Province, Hangzhou, China;* [3]*Department of Nutrition and Food Hygiene, Hubei Key Laboratory of Food Nutrition and Safety, School of Public Health, Tongji Medical College, Huazhong University of Science and Technology, Wuhan, China;* [4]*Center for Sleep and Circadian Medicine, The Affiliated Brain Hospital of Guangzhou Medical University, Guangzhou, China; Department of Psychiatry, Faculty of Medicine, The Chinese University of Hong Kong, Hong Kong Special Administrative Region, China;* [5]*Institute and Department of Endocrinology and Metabolism, Shanghai Ninth People's Hospital, Shanghai Jiao Tong University School of Medicine, Shanghai, China;* [6]*Department of Medical Sciences, Uppsala University, Uppsala, Sweden*

Abbreviations

HR, hazard ratio; MASLD, metabolic dysfunction-associated steatotic liver disease; PDI, plant-based diet index; PRS, polygenic risk score; PUFAs, polyunsaturated fatty acids; SFAs, saturated fatty acids; SNPs, single nucleotide polymorphisms.

Financial support

This research was supported by ZJU 100 Young Professor Project (X.T.), Rut and Arvid Wolff Memorial Foundation (X.T., 2023-02467). The funders had no role in the conduct of the study; collection, management, analysis, or interpretation of the data; preparation, review, or approval of the manuscript; or decision to submit the manuscript for publication.

Conflict of interest

None of the authors has any potential conflict of interest.

Please refer to the accompanying ICMJE disclosure forms for further details.

Authors' contributions

Hanzhang Wu: Conceptualization, Methodology, Software, Formal analysis, Investigation, Writing - Original Draft, Writing - Review & Editing, Visualization; Jiahe Wei: Methodology, Formal analysis, Investigation, Writing - Review & Editing; Shuai Wang: Investigation, Writing - Review & Editing; Liangkai Chen: Investigation, Writing - Review & Editing; Jihui Zhang: Investigation, Writing - Review & Editing; Ningjian Wang: Conceptualization, Investigation, Writing - Original Draft, Writing - Review & Editing; Xiao Tan: Conceptualization, Software, Formal analysis, Data Curation, Investigation, Writing - Original Draft, Writing - Review & Editing, Supervision, Project administration, Funding acquisition.

Data availability statement

UK Biobank resource data under application number 77740 was utilized for this research. The UK Biobank data is available on application (www.ukbiobank.ac.uk/).

Supplementary data

Supplementary data to this article can be found online at https://doi.org/10.1016/j.jhepr.2024.101133.

References

Author names in bold designate shared co-first authorship

[1] Powell EE, Wong VW, Rinella M. Non-alcoholic fatty liver disease. Lancet 2021;397:2212–2224.

[2] Younossi ZM. Non-alcoholic fatty liver disease - a global public health perspective. J Hepatol 2019;70:531–544.

[3] **Li J**, **Zou B**, Yeo YH, et al. Prevalence, incidence, and outcome of non-alcoholic fatty liver disease in Asia, 1999-2019: a systematic review and meta-analysis. Lancet Gastroenterol Hepatol 2019;4:389–398.

[4] Riazi K, Azhari H, Charette JH, et al. The prevalence and incidence of NAFLD worldwide: a systematic review and meta-analysis. Lancet Gastroenterol Hepatol 2022;7:851–861.

[5] Ratziu V, Goodman Z, Sanyal A. Current efforts and trends in the treatment of NASH. J Hepatol 2015;62:S65–S75.

[6] Romero-Gomez M, Zelber-Sagi S, Trenell M. Treatment of NAFLD with diet, physical activity and exercise. J Hepatol 2017;67:829–846.

[7] Cheng S, Ge J, Zhao C, et al. Effect of aerobic exercise and diet on liver fat in pre-diabetic patients with non-alcoholic-fatty-liver-disease: a randomized controlled trial. Sci Rep 2017;7:15952.

[8] **Liu Z**, **Huang H**, Ruan J, et al. The sulfur microbial diet and risk of nonalcoholic fatty liver disease: a prospective gene-diet study from the UK Biobank. Am J Clin Nutr 2024;119:417–424.

[9] **Zhang S**, **Gu Y**, Bian S, et al. Dietary patterns and risk of non-alcoholic fatty liver disease in adults: a prospective cohort study. Clin Nutr 2021;40:5373–5382.

[10] Lv Y, Rong S, Deng Y, et al. Plant-based diets, genetic predisposition and risk of non-alcoholic fatty liver disease. BMC Med 2023;21:351.

[11] Watanabe M, Tozzi R, Risi R, et al. Beneficial effects of the ketogenic diet on nonalcoholic fatty liver disease: a comprehensive review of the literature. Obes Rev 2020;21:e13024.

[12] Willett W, Rockstrom J, Loken B, et al. Food in the Anthropocene: the EAT-Lancet Commission on healthy diets from sustainable food systems. Lancet 2019;393:447–492.

[13] Shoaie S, Ghaffari P, Kovatcheva-Datchary P, et al. Quantifying diet-induced metabolic changes of the human gut microbiome. Cell Metab 2015;22:320–331.

[14] McGlinchey AJ, Govaere O, Geng D, et al. Metabolic signatures across the full spectrum of non-alcoholic fatty liver disease. JHEP Rep 2022;4:100477.

[15] Gagnon E, Manikpurage HD, Mitchell PL, et al. Large-scale metabolomic profiling and incident non-alcoholic fatty liver disease. iScience 2023;26:107127.

[16] Kalhan SC, Guo L, Edmison J, et al. Plasma metabolomic profile in nonalcoholic fatty liver disease. Metabolism 2011;60:404–413.

[17] Jonas W, Schurmann A. Genetic and epigenetic factors determining NAFLD risk. Mol Metab 2021;50:101111.

[18] Lv Y, Ding D, Luo M, et al. Physical activity intensity, genetic predisposition, and risk of nonalcoholic fatty liver disease: a prospective cohort study. Clin Gastroenterol Hepatol 2023;21:3444–3447. e3442.

[19] Ge X, Wang X, Yan Y, et al. Behavioural activity pattern, genetic factors, and the risk of nonalcoholic fatty liver disease: a prospective study in the UK Biobank. Liver Int 2023;43:1287–1297.

[20] Sudlow C, Gallacher J, Allen N, et al. UK biobank: an open access resource for identifying the causes of a wide range of complex diseases of middle and old age. Plos Med 2015;12:e1001779.

[21] Bradbury KE, Young HJ, Guo W, et al. Dietary assessment in UK Biobank: an evaluation of the performance of the touchscreen dietary questionnaire. J Nutr Sci 2018;7:e6.

[22] Liu B, Young H, Crowe FL, et al. Development and evaluation of the Oxford WebQ, a low-cost, web-based method for assessment of previous 24 h dietary intakes in large-scale prospective studies. Public Health Nutr 2011;14:1998–2005.

[23] Greenwood DC, Hardie LJ, Frost GS, et al. Validation of the Oxford WebQ online 24-hour dietary questionnaire using biomarkers. Am J Epidemiol 2019;188:1858–1867.

[24] Wu H, Gu Y, Meng G, et al. Relationship between dietary pattern and depressive symptoms: an international multicohort study. Int J Behav Nutr Phys Act 2023;20:74.

[25] Li J, Guasch-Ferre M, Chung W, et al. The Mediterranean diet, plasma metabolome, and cardiovascular disease risk. Eur Heart J 2020;41: 2645–2656.
[26] Hagstrom H, Adams LA, Allen AM, et al. Administrative coding in electronic health care record-based research of NAFLD: an expert panel consensus statement. Hepatology 2021;74:474–482.
[27] **Bycroft C**, **Freeman C**, **Petkova D**, et al. The UK Biobank resource with deep phenotyping and genomic data. Nature 2018;562:203–209.
[28] Trepo E, Valenti L. Update on NAFLD genetics: from new variants to the clinic. J Hepatol 2020;72:1196–1209.
[29] **Foster HME**, **Celis-Morales CA**, Nicholl BI, et al. The effect of socioeconomic deprivation on the association between an extended measurement of unhealthy lifestyle factors and health outcomes: a prospective analysis of the UK Biobank cohort. Lancet Public Health 2018;3:e576–e585.
[30] Zhang S, Stubbendorff A, Ericson U, et al. The EAT-Lancet diet, genetic susceptibility and risk of atrial fibrillation in a population-based cohort. BMC Med 2023;21:280.
[31] Zhang S, Stubbendorff A, Olsson K, et al. Adherence to the EAT-Lancet diet, genetic susceptibility, and risk of type 2 diabetes in Swedish adults. Metabolism 2023;141:155401.
[32] Ibsen DB, Christiansen AH, Olsen A, et al. Adherence to the EAT-lancet diet and risk of stroke and stroke subtypes: a cohort study. Stroke 2022;53: 154–163.
[33] Stubbendorff A, Sonestedt E, Ramne S, et al. Development of an EAT-Lancet index and its relation to mortality in a Swedish population. Am J Clin Nutr 2022;115:705–716.
[34] Alferink LJM, Erler NS, de Knegt RJ, et al. Adherence to a plant-based, high-fibre dietary pattern is related to regression of non-alcoholic fatty liver disease in an elderly population. Eur J Epidemiol 2020;35:1069–1085.
[35] **Guo W**, **Ge X**, Lu J, et al. Diet and risk of non-alcoholic fatty liver disease, cirrhosis, and liver cancer: a large prospective cohort study in UK biobank. Nutrients 2022;14:5335.
[36] Girona J, Amigo N, Ibarretxe D, et al. HDL triglycerides: a new marker of metabolic and cardiovascular risk. Int J Mol Sci 2019;20:3151.
[37] Verwer BJ, Scheffer PG, Vermue RP, et al. NAFLD is related to post-prandial triglyceride-enrichment of HDL particles in association with endothelial and HDL dysfunction. Liver Int 2020;40:2439–2444.
[38] Rosqvist F, Iggman D, Kullberg J, et al. Overfeeding polyunsaturated and saturated fat causes distinct effects on liver and visceral fat accumulation in humans. Diabetes 2014;63:2356–2368.
[39] Rosqvist F, Kullberg J, Stahlman M, et al. Overeating saturated fat promotes fatty liver and ceramides compared with polyunsaturated fat: a randomized trial. J Clin Endocrinol Metab 2019;104:6207–6219.
[40] **He K**, **Guo LL**, **Tang H**, et al. A freshwater fish-based diet alleviates liver steatosis by modulating gut microbiota and metabolites: a clinical randomized controlled trial in Chinese participants with nonalcoholic fatty liver disease. Am J Gastroenterol 2022;117:1621–1631.
[41] Karusheva Y, Koessler T, Strassburger K, et al. Short-term dietary reduction of branched-chain amino acids reduces meal-induced insulin secretion and modifies microbiome composition in type 2 diabetes: a randomized controlled crossover trial. Am J Clin Nutr 2019;110:1098–1107.
[42] Lin Z, Cai F, Lin N, et al. Effects of glutamine on oxidative stress and nuclear factor-kappaB expression in the livers of rats with nonalcoholic fatty liver disease. Exp Ther Med 2014;7:365–370.
[43] Sellmann C, Baumann A, Brandt A, et al. Oral supplementation of glutamine attenuates the progression of nonalcoholic steatohepatitis in C57BL/6J mice. J Nutr 2017;147:2041–2049.
[44] Guasch-Ferre M, Bhupathiraju SN, Hu FB. Use of metabolomics in improving assessment of dietary intake. Clin Chem 2018;64:82–98.
[45] Kenneth J, Rothman SG, Lash Timothy L. Modern epidemiology. Third Edition. Lippincott Williams & Wilkins; 2008.

Keywords: MASLD; EAT-lancet diet; Metabolite signature; Dietary metabolic response; Polygenic risk score.
Received 26 February 2024; received in revised form 24 May 2024; accepted 30 May 2024; Available online 10 June 2024

Journal of Hepatology, Volume *6*

Supplemental information

Dietary pattern modifies the risk of MASLD through metabolomic signature

Hanzhang Wu, Jiahe Wei, Shuai Wang, Liangkai Chen, Jihui Zhang, Ningjian Wang, and Xiao Tan

Dietary pattern modifies the risk of metabolic dysfunction-associated steatotic liver disease through metabolomic signature

Hanzhang Wu, Jiahe Wei, Shuai Wang, Liangkai Chen, Jihui Zhang, Ningjian Wang, Xiao Tan

Table of contents

Supplementary Methods…………………………………………………………….3

Fig. S1…………………………………………………………………………….6

Fig. S2…………………………………………………………………………….7

Fig. S3…………………………………………………………………………….8

Fig. S4…………………………………………………………………………….9

Fig. S5…………………………………………………………………………….10

Fig. S6…………………………………………………………………………….11

Table S1…………………………………………………………………………..12

Table S2…………………………………………………………………………..15

Table S3…………………………………………………………………………..23

Table S4…………………………………………………………………………..24

Table S5………………………………………………………………………….. 25

Table S6…………………………………………………………………………..26

Table S7…………………………………………………………………………..28

Table S8…………………………………………………..………………..………..…..... 29

Table S9…………………………………………………..……………………………......30

Supplementary Methods

Study population

The dataset collected comprehensive information on participant demographics, lifestyle factors, physical activity levels, medical history, hospital records, and mortality data. This study received ethical approval from the National Information Governance Board for Health and Social Care and the National Health Service North West Multicenter Research Ethics Committee. Prior to the baseline assessment, all participants provided informed consent through electronic signature.

Dietary assessment and EAT-Lancet diet index

We calculated the EAT-Lancet diet index using the established method conceptualized by Stubbendorff et al. [1], which was used to estimate the adherence to the EAT-Lancet Commission recommendations. The index includes 14 food groups, categorized into 7 emphasized foods (vegetables, fruits, unsaturated oils, legumes, nuts, whole grains, and fish) and 7 limited foods (beef and lamb, pork, poultry, eggs, dairy, potatoes, and added sugar) (Table S1). Scoring for emphasized foods ranges from 0 (indicating the lowest adherence) to 3 (the highest adherence). Conversely, for the limited foods, this scoring pattern was reversed, with 3 representing the lowest adherence and 0 the highest (Table S1). The scores of the 14 food groups for an individual were aggregated to calculate the EAT-Lancet diet index, which has a theoretical range from 0 to 42. A higher index signifies greater adherence to the EAT-Lancet dietary patterns. Additionally, there was a significant correlation between the initial dietary assessment EAT-Lancet diet index and the averaged dietary assessment

EAT-Lancet diet index (Pearson correlation coefficient = 0.84, $P < 0.0001$). Therefore, if participants completed dietary assessments multiple times, the study employed both the earliest dietary consumption date and the averaged dietary assessment EAT-Lancet diet index to maximize follow-up time.

Metabolomics measurement

A high-throughput nuclear magnetic resonance (NMR) metabolomics platform was used to analyze baseline plasma samples from approximately 280,000 randomly selected UK Biobank participants. Detailed protocols on sample collection and metabolomic quantification are presented elsewhere [2-4]. This simultaneously quantified 251 metabolic biomarkers (170 directly measured and 81 ratios of these). The biomarkers investigated in this study encompass multiple metabolic pathways. These include lipoprotein lipids in 14 subclasses, fatty acids and their compositions, as well as various low-molecular weight metabolites, such as amino acids, ketone bodies, and glycolysis metabolites quantified in molar concentration units. In the present study, we included a subset of 170 metabolic biomarkers (Table S2) that were directly measured for subsequent analyses. Metabolites with less than 10% missing were included, and missing data for each metabolite were imputed using half of the minimum measured value. Metabolites were highly correlated within each category (Fig. S2).

Polygenic risk score for MASLD

Based on the selected SNPs, the polygenic risk score (PRS) for MASLD was calculated as follows: $PRS = \beta1 \times SNP1 + \beta2 \times SNP2 + \ldots + \beta n \times SNPn$, where SNPn is the risk allele number of each SNP. A higher PRS indicates a higher genetic

predisposition to the disease. Participants were further divided into low (quintile 1), medium (quintiles 2–4), and high (quintile 5) PRS groups based on the quintiles of the PRS.

Statistical analysis

Models were adjusted for age, sex, BMI, smoking status, alcohol intake, education levels, Townsend deprivation index, total energy consumption, physical activity, individual history of the disease (cancer, cardiovascular disease, hypertension and diabetes), fasting duration, spectrometer, MASLD-PRS, first 10 principal components of ancestry, and genotype measurement batch. We also estimated the joint association of EAT-Lancet diet index and PRS with MASLD risk by creating a combined variable based on categories of PRS and EAT-Lancet diet index (12 categories). The reference group consisted of individuals with the highest risk combination, which involved having the lowest EAT-Lancet diet index and the highest PRS. In addition, we performed mediation analyses using the method proposed by Lange et al. [5] to evaluate the mediation of the association between EAT-Lancet diet and risk of MASLD by metabolite signature score or selected metabolites.

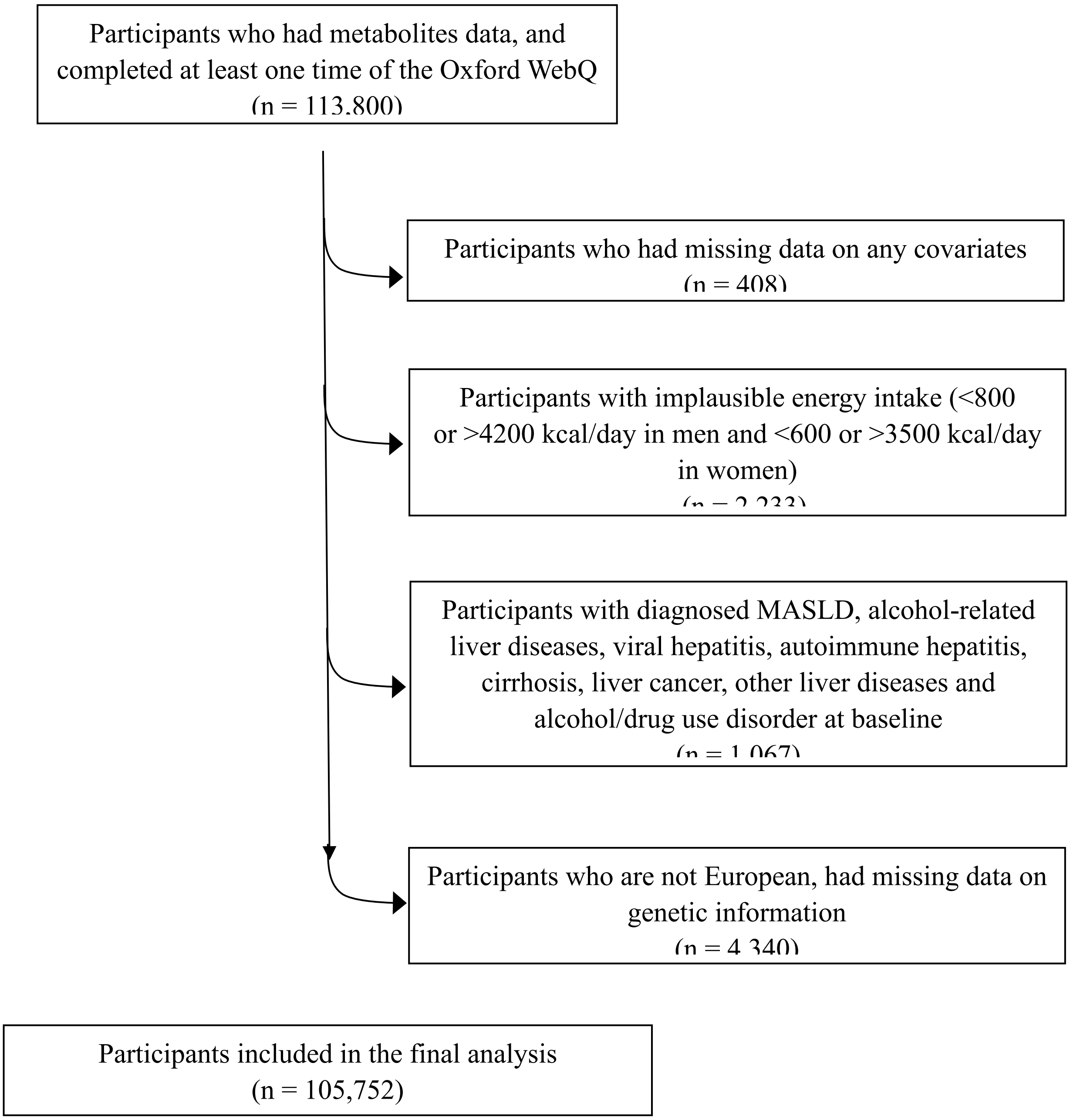

Fig. S1. Selection of study participants in the UK Biobank Cohort.

Fig. S2. Marginal correlation matrix for all the 170 metabolites included in the analysis.

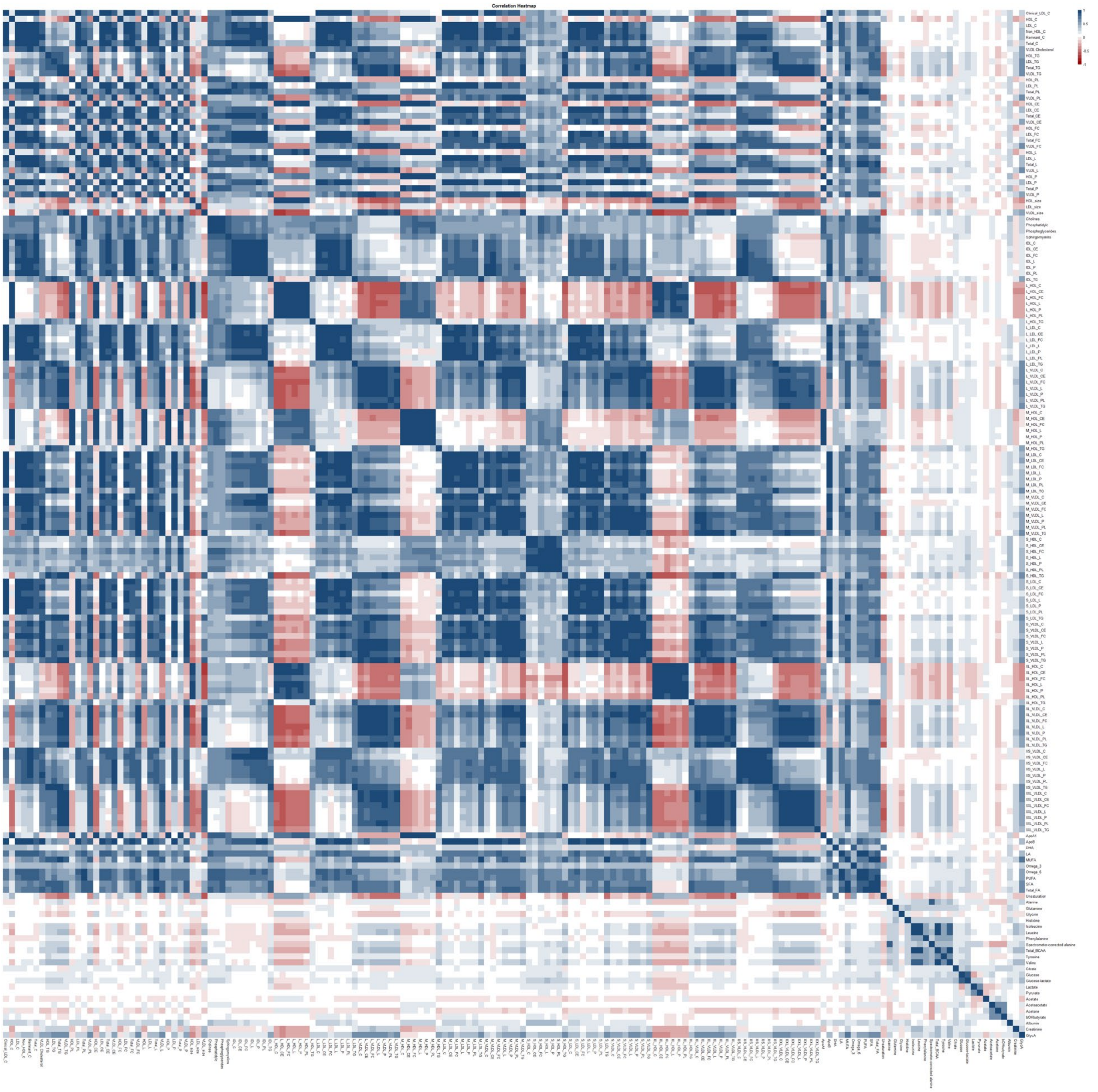

Fig. S3. Restricted cubic spline models for the association between (A) the EAT-Lancet diet index, (B) metabolic signature and risk of MASLD. The 95% CIs of the adjusted HRs are represented by the shaded are. Restricted cubic spline model is adjusted the same as the Model 2 in the Table 2. A two-tailed p <0.05 was considered statistically significant.

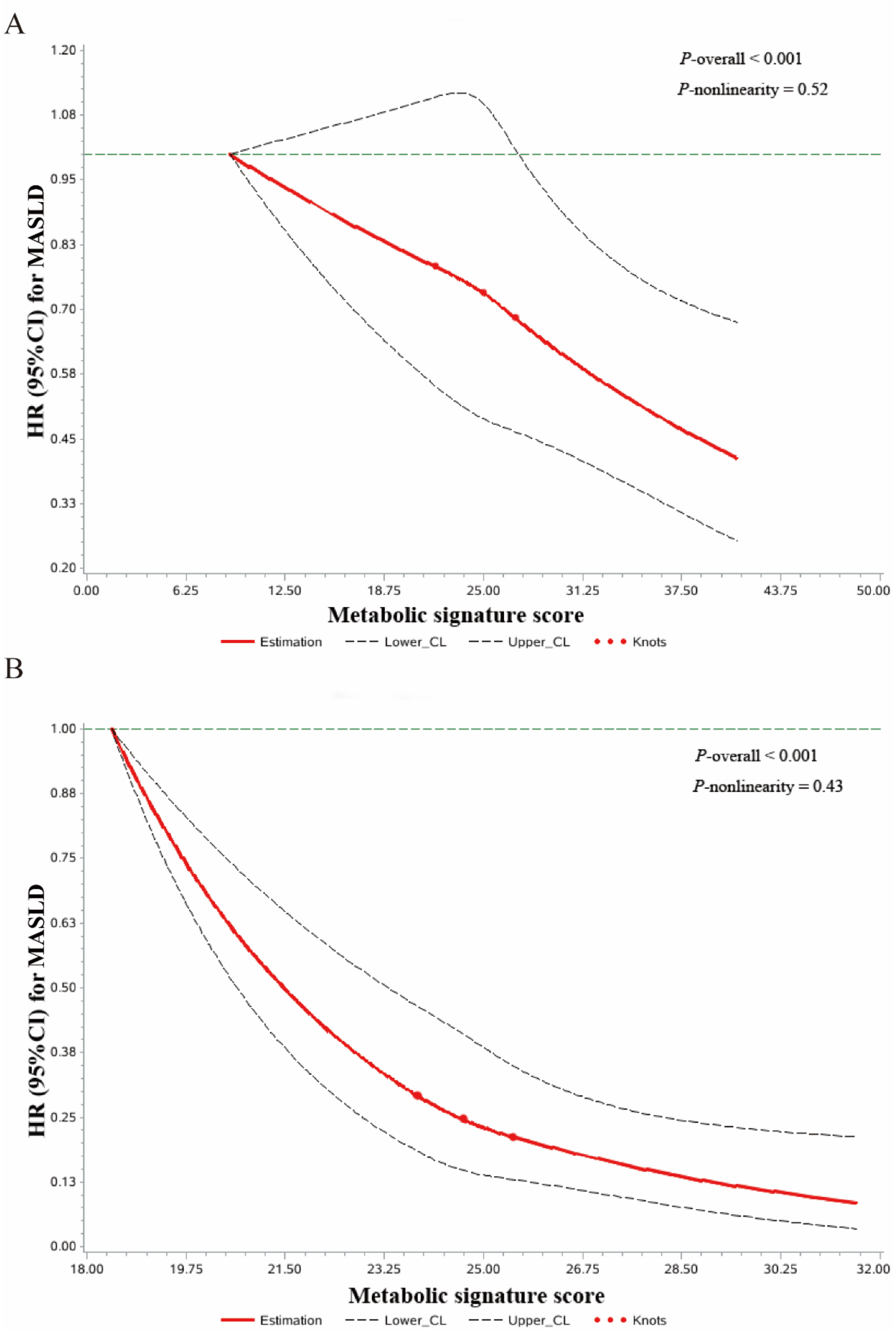

Fig. S4. Joint association of the EAT-Lancet diet index and genetic susceptibility with risk of MASLD. Adjusted for age, sex, BMI, total energy intake, smoking status, alcohol intake, educational level, Townsend deprivation index, physical activity, hypertension, diabetes, cancer, cardiovascular disease, fasting duration, spectrometer, first 10 principal components of ancestry, and genotype measurement batch.

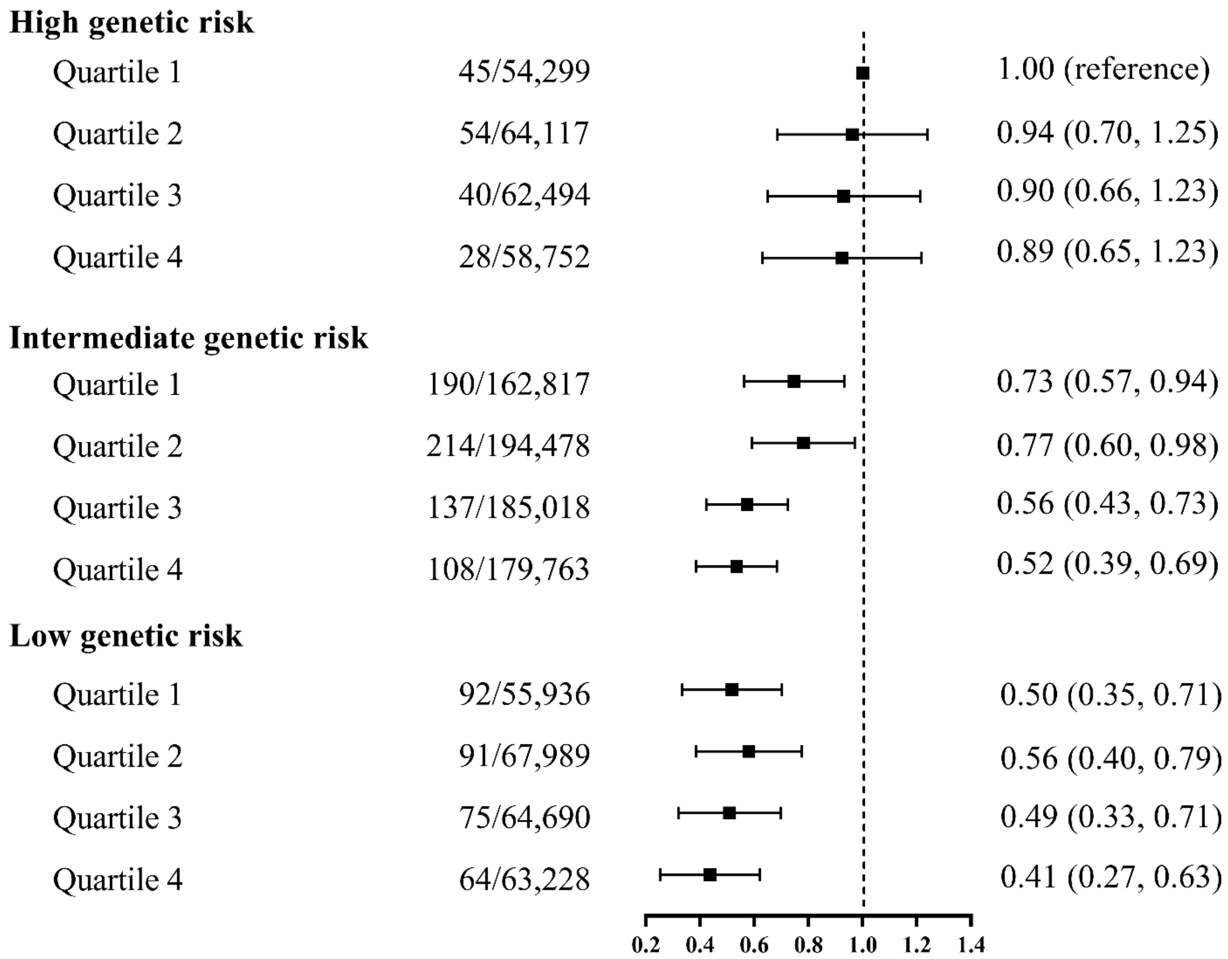

Fig. S5. Correlation between the EAT-Lancet diet and the corresponding metabolite signature score in repeated assessments.

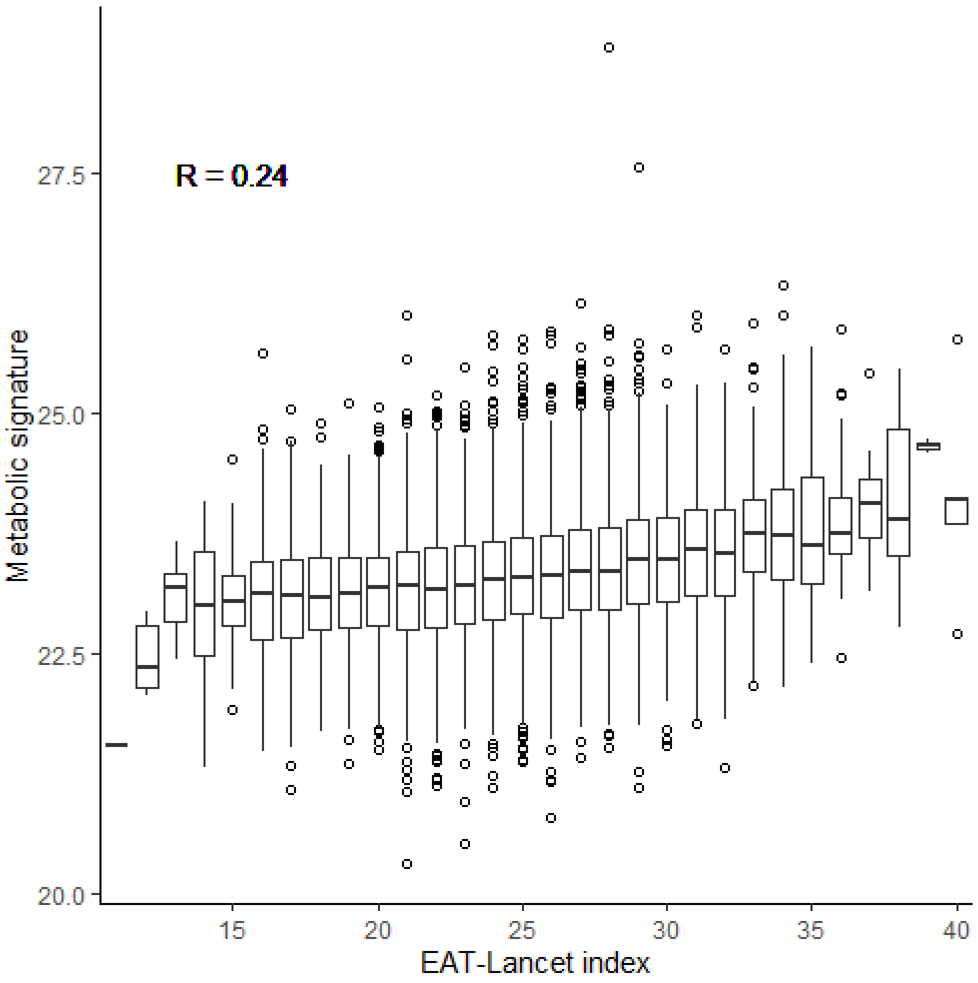

Fig. S6. Adjusted HR (95% CI) for per 1-score EAT-Lancet diet index increment and risk of MASLD stratified by potential risk factors. All models were adjusted the same as the Model 2 in the table 2. A two-tailed p <0.05 was considered statistically significant.

Risk factor	Pre 1-score increment of EAT-Lancet diet index	P for interaction
Age		0.22
< 60 years	0.95 (0.93,0.98)	
≥ 60 years	0.97 (0.94,1.00)	
Sex		0.96
Male	0.96 (0.93,0.99)	
Female	0.96 (0.93,0.99)	
BMI		0.12
< 30 kg/m²	0.96 (0.93,0.98)	
≥ 30 kg/m²	0.97 (0.94,1.00)	
Education		0.10
< College	0.97 (0.95,0.99)	
≥ College	0.94 (0.92,0.97)	
Physical activity		0.89
< 25 MET·h/wk	0.96 (0.93,0.98)	
≥ 25 MET·h/wk	0.97 (0.94,1.00)	
Alconol intake		0.10
No	0.97 (0.94,1.00)	
Yes	0.95 (0.92,0.98)	
Smokeing		0.08
Never	0.97 (0.95,1.00)	
Past	0.96 (0.93,0.99)	
Current	0.91 (0.85,0.97)	
Hypertension		0.51
No	0.96 (0.94,0.99)	
Yes	0.96 (0.93,1.00)	
Diabetes		0.83
No	0.97 (0.94,0.99)	
Yes	0.92 (0.86,0.98)	

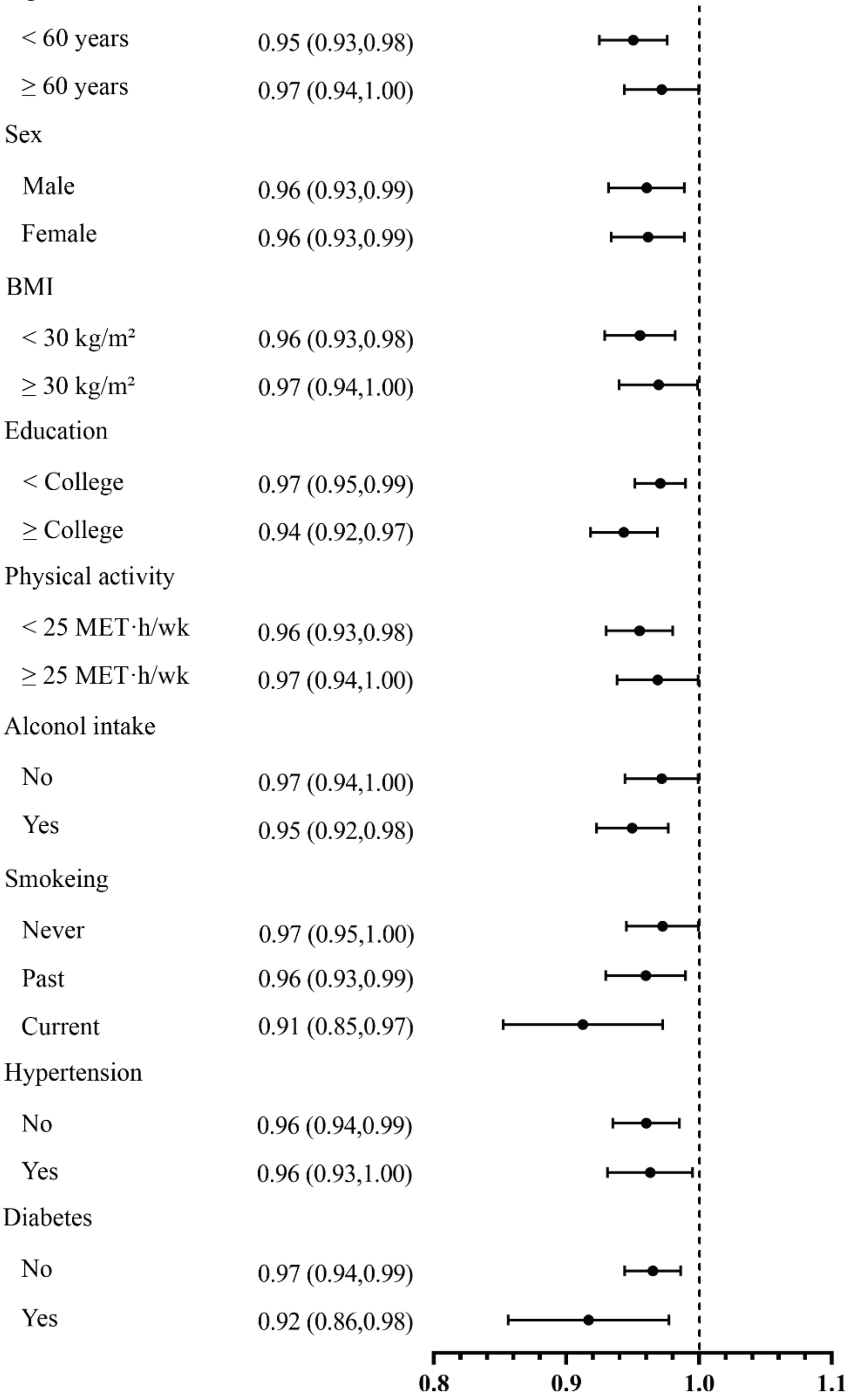

Table S1. Criteria for the EAT-Lancet diet index constructed to evaluate the EAT-Lancet diet and example of food items in the UK Biobank [a]

Food components in the EAT-Lancet diet index [b]		Target intake (reference interval)	3 points	2 points	1 point	0 points	Food items
Emphasized intake	Vegetables	300 (200–600)	>300	200–300	100–200	<100	Mixed vegetables, vegetable pieces, coleslaw, side salad, avocado, beetroot, broccoli, butternut squash, cabbage/kale, carrots, cauliflower, celery, courgette, cucumber, garlic, leeks, lettuce, mushrooms, onion, parsnip, sweet peppers, spinach, sprouts, sweetcorn, fresh tomatoes, cooked or tinned tomatoes, turnip/swede, watercress, other vegetable intake
	Fruits	200 (100–300)	>200	100–200	50–100	<50	Stewed fruit, prune, dried fruit, mixed fruit, apple, banana, berries, cherries, grapefruit, grapes, mango, melon, orange, orange-like small fruits, peach/nectarine, pear, pineapple, plum, other fruits, olives
	Unsaturated oils [c]	40 (20–80)	>40	20–40	10–20	<10	Monounsaturated fatty acids, n-3 fatty acids, n-6 fatty acids
	Legumes	75 (0–150)	>75	37.5–75	18.75–37.5	<18.75	Vegetarian sausages/burgers, tofu, quorn, other vegetarian alternative, baked beans, pulse, broad beans, green beans, peas
	Nuts	50 (0–100)	>50	25–50	12.5–25	<12.5	Salted peanuts, unsalted peanuts, salted nuts, unsalted nuts, seeds

	Whole grains	232	>232	116–232	58–116	<58	Porridge, muesli, plain cereal, bran cereal, whole-wheat cereal, oatcakes, wholemeal bread (flour type: wholemeal), whole meal pasta, brown rice, couscous, other cooked grains (such as bulgur).
	Fish	28 (0–100)	>28	14–28	7–14	<7	Tinned tuna, oily fish, breaded fish, battered fish, white fish, prawns, lobster/crab, shellfish, other fish intake
Limited intake	Beef and lamb	7 (0–14)	<7	7–14	14–28	>28	beef, lamb
	Pork	7 (0–14)	<7	7–14	14–28	>28	Sausage, pork, bacon, ham, liver, other meat intake
	Poultry	29 (0–58)	<29	29–58	58–116	>116	crumbed or deep-fried poultry, poultry
	Eggs	13 (0–25)	<13	13–25	25–50	>50	Whole eggs, omelettes or scrambled egg, eggs in sandwiches, scotch egg, other egg dishes
	Dairy	250 (0–500)	<250	250–500	500–1000	>1000	Milk, dairy smoothie, flavored milk, yogurt, ice-cream, cheesecake, milk-based pudding, other milk-based pudding, low fat hard cheese, hard cheese, soft cheese, blue cheese, low fat cheese spread, cheese spread, cottage cheese, feta cheese, mozzarella cheese, goat's cheese, other cheese
	Potatoes	50 (0–100)	<50	50–100	100–200	>200	Fried potatoes, boiled/baked potatoes, mashed potatoes, crisps (e.g., potato chips)
	Added sugar [d]	31 (0–31)	<31	31–62	62–124	>124	Added sugars and preserves

[a] Established method conceptualized by Stubbendorff et al

[b] Food components in the index are based on the EAT-Lancet diet as grams per day, with some modifications.

[c] Fat intake and quality are reflected as the consumption of unsaturated fatty acids from overall diet, since too little information about the types of oils were recorded in the 24-hour diet recall.

[d] Derived from sweet foods and beverages, and sugar added to food or beverages recorded in the 24-hour diet recall.

Table S2. Metabolic biomarkers and abbreviations included the UK Biobank

**Metabolite**	**Type**	**Abbreviations**	**Field ID**
Glucose-lactate	Glycolysis related metabolites	Glucose-lactate	20280
Spectrometer-corrected alanine	Amino acids	Spectrometer-corrected alanine	20281
Total Cholesterol	Cholesterol	Total_C	23400
Total Cholesterol Minus HDL-C	Cholesterol	Non_HDL_C	23401
Remnant Cholesterol (Non-HDL, Non-LDL -Cholesterol)	Cholesterol	Remnant_C	23402
VLDL Cholesterol	Cholesterol	VLDL Cholesterol	23403
Clinical LDL Cholesterol	Cholesterol	Clinical_LDL_C	23404
LDL Cholesterol	Cholesterol	LDL_C	23405
HDL Cholesterol	Cholesterol	HDL_C	23406
Total Triglycerides	Triglycerides	Total_TG	23407
Triglycerides in VLDL	Triglycerides	VLDL_TG	23408
Triglycerides in LDL	Triglycerides	LDL_TG	23409
Triglycerides in HDL	Triglycerides	HDL_TG	23410
Total Phospholipids in Lipoprotein Particles	Phospholipids	Total_PL	23411
Phospholipids in VLDL	Phospholipids	VLDL_PL	23412
Phospholipids in LDL	Phospholipids	LDL_PL	23413
Phospholipids in HDL	Phospholipids	HDL_PL	23414
Total Esterified Cholesterol	Cholesteryl Esters	Total_CE	23415
Cholesteryl Esters in VLDL	Cholesteryl Esters	VLDL_CE	23416
Cholesteryl Esters in LDL	Cholesteryl Esters	LDL_CE	23417
Cholesteryl Esters in HDL	Cholesteryl Esters	HDL_CE	23418

Total Free Cholesterol	Free Cholesterol	Total_FC	23419
Free Cholesterol in VLDL	Free Cholesterol	VLDL_FC	23420
Free Cholesterol in LDL	Free Cholesterol	LDL_FC	23421
Free Cholesterol in HDL	Free Cholesterol	HDL_FC	23422
Total Lipids in Lipoprotein Particles	Total Lipids	Total_L	23423
Total Lipids in VLDL	Total Lipids	VLDL_L	23424
Total Lipids in LDL	Total Lipids	LDL_L	23425
Total Lipids in HDL	Total Lipids	HDL_L	23426
Total Concentration of Lipoprotein Particles	Lipoprotein Particle Concentrations	Total_P	23427
Concentration of VLDL Particles	Lipoprotein Particle Concentrations	VLDL_P	23428
Concentration of LDL Particles	Lipoprotein Particle Concentrations	LDL_P	23429
Concentration of HDL Particles	Lipoprotein Particle Concentrations	HDL_P	23430
Average Diameter for VLDL Particles	Lipoprotein Particle Size	VLDL_size	23431
Average Diameter for LDL Particles	Lipoprotein Particle Size	LDL_size	23432
Average Diameter for HDL Particles	Lipoprotein Particle Size	HDL_size	23433
Phosphoglycerides	Other lipids	Phosphoglycerides	23434
Total Cholines	Other lipids	Cholines	23436
Phosphatidylcholines	Other lipids	Phosphatidylc	23437
Sphingomyelins	Other lipids	Sphingomyelins	23438
Apolipoprotein B	Apolipoproteins	ApoB	23439
Apolipoprotein A1	Apolipoproteins	ApoA1	23440
Total Fatty Acids	Fatty Acids	Total_FA	23442

Degree of Unsaturation	Fatty Acids	Unsaturation	23443
Omega-3 Fatty Acids	Fatty Acids	Omega_3	23444
Omega-6 Fatty Acids	Fatty Acids	Omega_6	23445
Polyunsaturated Fatty Acids	Fatty Acids	PUFA	23446
Monounsaturated Fatty Acids	Fatty Acids	MUFA	23447
Saturated Fatty Acids	Fatty Acids	SFA	23448
Linoleic Acid	Fatty Acids	LA	23449
Docosahexaenoic Acid	Fatty Acids	DHA	23450
Alanine	Amino acids	Alanine	23460
Glutamine	Amino acids	Glutamine	23461
Glycine	Amino acids	Glycine	23462
Histidine	Amino acids	Histidine	23463
Total Concentration of Branched-Chain Amino Acids (Leucine + Isoleucine + Valine)	Amino acids	Total_BCAA	23464
Isoleucine	Amino acids	Isoleucine	23465
Leucine	Amino acids	Leucine	23466
Valine	Amino acids	Valine	23467
Phenylalanine	Amino acids	Phenylalanine	23468
Tyrosine	Amino acids	Tyrosine	23469
Glucose	Glycolysis related metabolites	Glucose	23470
Lactate	Glycolysis related metabolites	Lactate	23471
Pyruvate	Glycolysis related metabolites	Pyruvate	23472

Citrate	Glycolysis related metabolites	Citrate	23473
3-Hydroxybutyrate	Ketone bodies	bOHbutyrate	23474
Acetate	Ketone bodies	Acetate	23475
Acetoacetate	Ketone bodies	Acetoacetate	23476
Acetone	Ketone bodies	Acetone	23477
Creatinine	Fluid balance	Creatinine	23478
Albumin	Fluid balance	Albumin	23479
Glycoprotein Acetyls	Fluid balance	GlycA	23480
Concentration of Chylomicrons and Extremely Large VLDL Particles	Lipoprotein subclasses	XXL_VLDL_P	23481
Total Lipids in Chylomicrons and Extremely Large VLDL	Lipoprotein subclasses	XXL_VLDL_L	23482
Phospholipids in Chylomicrons and Extremely Large VLDL	Lipoprotein subclasses	XXL_VLDL_PL	23483
Cholesterol in Chylomicrons and Extremely Large VLDL	Lipoprotein subclasses	XXL_VLDL_C	23484
Cholesteryl Esters in Chylomicrons and Extremely Large VLDL	Lipoprotein subclasses	XXL_VLDL_CE	23485
Free Cholesterol in Chylomicrons and Extremely Large VLDL	Lipoprotein subclasses	XXL_VLDL_FC	23486
Triglycerides in Chylomicrons and Extremely Large VLDL	Lipoprotein subclasses	XXL_VLDL_TG	23487
Concentration of Very Large VLDL Particles	Lipoprotein subclasses	XL_VLDL_P	23488
Total Lipids in Very Large VLDL	Lipoprotein subclasses	XL_VLDL_L	23489
Phospholipids in Very Large VLDL	Lipoprotein subclasses	XL_VLDL_PL	23490
Cholesterol in Very Large VLDL	Lipoprotein subclasses	XL_VLDL_C	23491
Cholesteryl Esters in Very Large VLDL	Lipoprotein subclasses	XL_VLDL_CE	23492
Free Cholesterol in Very Large VLDL	Lipoprotein subclasses	XL_VLDL_FC	23493
Triglycerides in Very Large VLDL	Lipoprotein subclasses	XL_VLDL_TG	23494
Concentration of Large VLDL Particles	Lipoprotein subclasses	L_VLDL_P	23495
Total Lipids in Large VLDL	Lipoprotein subclasses	L_VLDL_L	23496
Phospholipids in Large VLDL	Lipoprotein subclasses	L_VLDL_PL	23497

Cholesterol in Large VLDL	Lipoprotein subclasses	L_VLDL_C	23498
Cholesteryl Esters in Large VLDL	Lipoprotein subclasses	L_VLDL_CE	23499
Free Cholesterol in Large VLDL	Lipoprotein subclasses	L_VLDL_FC	23500
Triglycerides in Large VLDL	Lipoprotein subclasses	L_VLDL_TG	23501
Concentration of Medium VLDL Particles	Lipoprotein subclasses	M_VLDL_P	23502
Total Lipids in Medium VLDL	Lipoprotein subclasses	M_VLDL_L	23503
Phospholipids in Medium VLDL	Lipoprotein subclasses	M_VLDL_PL	23504
Cholesterol in Medium VLDL	Lipoprotein subclasses	M_VLDL_C	23505
Cholesteryl Esters in Medium VLDL	Lipoprotein subclasses	M_VLDL_CE	23506
Free Cholesterol in Medium VLDL	Lipoprotein subclasses	M_VLDL_FC	23507
Triglycerides in Medium VLDL	Lipoprotein subclasses	M_VLDL_TG	23508
Concentration of Small VLDL Particles	Lipoprotein subclasses	S_VLDL_P	23509
Total Lipids in Small VLDL	Lipoprotein subclasses	S_VLDL_L	23510
Phospholipids in Small VLDL	Lipoprotein subclasses	S_VLDL_PL	23511
Cholesterol in Small VLDL	Lipoprotein subclasses	S_VLDL_C	23512
Cholesteryl Esters in Small VLDL	Lipoprotein subclasses	S_VLDL_CE	23513
Free Cholesterol in Small VLDL	Lipoprotein subclasses	S_VLDL_FC	23514
Triglycerides in Small VLDL	Lipoprotein subclasses	S_VLDL_TG	23515
Concentration of Very Small VLDL Particles	Lipoprotein subclasses	XS_VLDL_P	23516
Total Lipids in Very Small VLDL	Lipoprotein subclasses	XS_VLDL_L	23517
Phospholipids in Very Small VLDL	Lipoprotein subclasses	XS_VLDL_PL	23518
Cholesterol in Very Small VLDL	Lipoprotein subclasses	XS_VLDL_C	23519
Cholesteryl Esters in Very Small VLDL	Lipoprotein subclasses	XS_VLDL_CE	23520
Free Cholesterol in Very Small VLDL	Lipoprotein subclasses	XS_VLDL_FC	23521
Triglycerides in Very Small VLDL	Lipoprotein subclasses	XS_VLDL_TG	23522
Concentration of IDL Particles	Lipoprotein subclasses	IDL_P	23523

Total Lipids in IDL	Lipoprotein subclasses	IDL_L	23524
Phospholipids in IDL	Lipoprotein subclasses	IDL_PL	23525
Cholesterol in IDL	Lipoprotein subclasses	IDL_C	23526
Cholesteryl Esters in IDL	Lipoprotein subclasses	IDL_CE	23527
Free Cholesterol in IDL	Lipoprotein subclasses	IDL_FC	23528
Triglycerides in IDL	Lipoprotein subclasses	IDL_TG	23529
Concentration of Large LDL Particles	Lipoprotein subclasses	L_LDL_P	23530
Total Lipids in Large LDL	Lipoprotein subclasses	L_LDL_L	23531
Phospholipids in Large LDL	Lipoprotein subclasses	L_LDL_PL	23532
Cholesterol in Large LDL	Lipoprotein subclasses	L_LDL_C	23533
Cholesteryl Esters in Large LDL	Lipoprotein subclasses	L_LDL_CE	23534
Free Cholesterol in Large LDL	Lipoprotein subclasses	L_LDL_FC	23535
Triglycerides in Large LDL	Lipoprotein subclasses	L_LDL_TG	23536
Concentration of Medium LDL Particles	Lipoprotein subclasses	M_LDL_P	23537
Total Lipids in Medium LDL	Lipoprotein subclasses	M_LDL_L	23538
Phospholipids in Medium LDL	Lipoprotein subclasses	M_LDL_PL	23539
Cholesterol in Medium LDL	Lipoprotein subclasses	M_LDL_C	23540
Cholesteryl Esters in Medium LDL	Lipoprotein subclasses	M_LDL_CE	23541
Free Cholesterol in Medium LDL	Lipoprotein subclasses	M_LDL_FC	23542
Triglycerides in Medium LDL	Lipoprotein subclasses	M_LDL_TG	23543
Concentration of Small LDL Particles	Lipoprotein subclasses	S_LDL_P	23544
Total Lipids in Small LDL	Lipoprotein subclasses	S_LDL_L	23545
Phospholipids in Small LDL	Lipoprotein subclasses	S_LDL_PL	23546
Cholesterol in Small LDL	Lipoprotein subclasses	S_LDL_C	23547
Cholesteryl Esters in Small LDL	Lipoprotein subclasses	S_LDL_CE	23548
Free Cholesterol in Small LDL	Lipoprotein subclasses	S_LDL_FC	23549

Triglycerides in Small LDL	Lipoprotein subclasses	S_LDL_TG	23550
Concentration of Very Large HDL Particles	Lipoprotein subclasses	XL_HDL_P	23551
Total Lipids in Very Large HDL	Lipoprotein subclasses	XL_HDL_L	23552
Phospholipids in Very Large HDL	Lipoprotein subclasses	XL_HDL_PL	23553
Cholesterol in Very Large HDL	Lipoprotein subclasses	XL_HDL_C	23554
Cholesteryl Esters in Very Large HDL	Lipoprotein subclasses	XL_HDL_CE	23555
Free Cholesterol in Very Large HDL	Lipoprotein subclasses	XL_HDL_FC	23556
Triglycerides in Very Large HDL	Lipoprotein subclasses	XL_HDL_TG	23557
Concentration of Large HDL Particles	Lipoprotein subclasses	L_HDL_P	23558
Total Lipids in Large HDL	Lipoprotein subclasses	L_HDL_L	23559
Phospholipids in Large HDL	Lipoprotein subclasses	L_HDL_PL	23560
Cholesterol in Large HDL	Lipoprotein subclasses	L_HDL_C	23561
Cholesteryl Esters in Large HDL	Lipoprotein subclasses	L_HDL_CE	23562
Free Cholesterol in Large HDL	Lipoprotein subclasses	L_HDL_FC	23563
Triglycerides in Large HDL	Lipoprotein subclasses	L_HDL_TG	23564
Concentration of Medium HDL Particles	Lipoprotein subclasses	M_HDL_P	23565
Total Lipids in Medium HDL	Lipoprotein subclasses	M_HDL_L	23566
Phospholipids in Medium HDL	Lipoprotein subclasses	M_HDL_PL	23567
Cholesterol in Medium HDL	Lipoprotein subclasses	M_HDL_C	23568
Cholesteryl Esters in Medium HDL	Lipoprotein subclasses	M_HDL_CE	23569
Free Cholesterol in Medium HDL	Lipoprotein subclasses	M_HDL_FC	23570
Triglycerides in Medium HDL	Lipoprotein subclasses	M_HDL_TG	23571
Concentration of Small HDL Particles	Lipoprotein subclasses	S_HDL_P	23572
Total Lipids in Small HDL	Lipoprotein subclasses	S_HDL_L	23573
Phospholipids in Small HDL	Lipoprotein subclasses	S_HDL_PL	23574
Cholesterol in Small HDL	Lipoprotein subclasses	S_HDL_C	23575

Cholesteryl Esters in Small HDL	Lipoprotein subclasses	S_HDL_CE	23576
Free Cholesterol in Small HDL	Lipoprotein subclasses	S_HDL_FC	23577
Triglycerides in Small HDL	Lipoprotein subclasses	S_HDL_TG	23578

Table S3. Definitions of MASLD and other liver diseases

	ICD-9	ICD-10
Nonalcoholic fatty liver disease	5718	K760
Nonalcoholic steatohepatitis		K758
Alcohol related liver disease	571, 5710, 5711, 5712, 5713	K70
Viral hepatitis	70	B16, B17, B18, B19
Autoimmune liver disease	5716, 5761	K830, K743, K754
Cirrhosis	27103, 4562, 5712, 5715, 57150, 57151, 57152, 57158, 57159, 5716	I850, I859, K703, K704, K717, K721, K744, K745, K746, K766, K767
Other liver diseases [a]	2750, 2751, 2776, 4530, 5714, 5716, 5715, 4561, 54621, 4560, 45620, 7895, 5722, 5724, 5723, 1550, 152,	E831, E830, E880, I820, K765, K739, K732, K744, K745, C220, C229
Alcohol/drug use disorder	303, 3050, 291, 3575, 4255, 5353, 9801, 9809, 3051, 3052, 3053, 3054, 3055, 3056, 3057, 3058, 3059	F10, E244, G621, I426, K292, G312, G721, K852, K860, T510, T519,F11, F12, F13, F14, F16, F18, F19

[a] Other liver diseases including liver diseases as well as liver cancer mentioned in ICD-9 and ICD-10 coding.

Table S4. SNPs used for creating the MASLD-PRS

Gene Locus	Chr	SNP	Risk allele	Reference allele	Beta
PNPLA3	22	rs738409	G	C	0.594
TM6SF2	19	rs58542926	T	C	0.166
MBOAT7	19	rs641738	T	C	0.073
GCKR	2	rs1260326	T	C	0.271
HSD17B13	4	rs72613567	T	TA	0.216

Abbreviations: Chr, chromosome; MASLD-PRS, metabolic dysfunction-associated steatotic liver disease-polygenic risk score; SNP, single nucleotide polymorphism.

Table S5. Association between MASLD-PRS and risk of MASLD [a]

	low MASLD-PRS	medium MASLD-PRS	high MASLD-PRS	per SD increment of MASLD-PRS
Median (min, max)	0.34 (0.00, 0.50)	0.78 (0.51, 1.23)	1.44 (1.23, 2.64)	
No. of MASLD	167	649	322	
Person years	239,662	722,076	251,844	
Incidence per 1000 PYs	0.70	0.90	1.28	
Model 1	1.00 (reference)	1.31 (1.11, 1.56)	1.88 (1.56, 2.27)	1.25 (1.18, 1.32)
Model 2	1.00 (reference)	1.33 (1.12, 1.58)	1.90 (1.57, 2.30)	1.25 (1.18, 1.32)

Abbreviations: BMI; body mass index; MASLD-PRS, metabolic dysfunction-associated steatotic liver disease-polygenic risk score; PYs, person-years.

a Values are hazard ratios (95% confidence interval) (all such values).

[b] *P* for trend was calculated across quartiles using multivariable Cox regression models.

Model 1 was adjusted for age, sex, and BMI.

Model 2 was additionally adjusted for total energy intake, smoking status, alcohol intake, educational level, Townsend deprivation index, physical activity, hypertension, diabetes, cancer, cardiovascular disease, fasting duration, spectrometer, first 10 principal components of ancestry, and genotype measurement batch.

Table S6. Subgroup analysis of the association between EAT-Lancet diet index and the risk of MASLD by genetic risk [a]

	Categories of the EAT-Lancet diet index				*P* for trend [c]
	Quartile 1	Quartile 2	Quartile 3	Quartile 4	
**Low MASLD-PRS**					
No. of MASLD	45	54	40	28	
Person years	54,299	64,117	62,494	58,752	
Incidence per 1000 PYs	0.83	0.84	0.64	0.48	
Model 1	1.00 (reference)	1.09 (0.73, 1.62) [b]	0.93 (0.60, 1.42)	0.76 (0.47, 1.22)	0.21
Model 2	1.00 (reference)	1.16 (0.77, 1.74)	0.99 (0.63, 1.54)	0.81 (0.49, 1.33)	0.46
**Medium MASLD-PRS**					
No. of MASLD	190	214	137	108	
Person years	162,817	194,478	185,018	179,763	
Incidence per 1000 PYs	1.17	1.10	0.74	0.60	
Model 1	1.00 (reference)	1.02 (0.84, 1.24)	0.73 (0.59, 0.92)	0.69 (0.54, 0.88)	<0.001
Model 2	1.00 (reference)	1.05 (0.86, 1.28)	0.77 (0.61, 0.96)	0.72 (0.56, 0.92)	<0.001
**High MASLD-PRS**					
No. of MASLD	92	91	75	64	
Person years	55,936	67,989	64,690	63,228	
Incidence per 1000 PYs	1.64	1.34	1.16	1.01	
Model 1	1.00 (reference)	0.89 (0.67, 1.19)	0.84 (0.61, 1.14)	0.81 (0.59, 1.12)	0.17
Model 2	1.00 (reference)	0.92 (0.69, 1.23)	0.88 (0.65, 1.20)	0.88 (0.63, 1.23)	0.38

Abbreviations: BMI, body mass index; MASLD-PRS, metabolic dysfunction-associated steatotic liver disease-polygenic risk score; PYs, person-years.

[a] Obtained by using multivariable Cox regression model.

[b] Hazard ratios (95% confidence interval) (all such values).

[c] P for trend was calculated across quartiles using multivariable Cox regression models. A two-tailed $p < 0.05$ was considered statistically significant.

Model 1 was adjusted for age, sex, and BMI.

Model 2 was additionally adjusted for total energy intake, smoking status, alcohol intake, educational level, Townsend deprivation index, physical activity, hypertension, diabetes, cancer, cardiovascular disease, fasting duration, spectrometer, MASLD-PRS, first 10 principal components of ancestry, and genotype measurement batch.

Table S7. Association between repeated assessments of metabolic signature with MASLD risk [a]

	Categories of the EAT-Lancet diet index				*P* for trend [c]
	Quartile 1	Quartile 2	Quartile 3	Quartile 4	
**Metabolic signature**					
No. of MASLD	49	21	13	16	
Person years	28,274	28,583	28,585	28,448	
Incidence per 1000 PYs	1.73	0.73	0.45	0.56	
Model 1	1.00 (reference)	0.48 (0.29, 0.81)	0.34 (0.18, 0.63)	0.49 (0.27, 0.87)	<0.001
Model 2	1.00 (reference)	0.51 (0.30, 0.85)	0.36 (0.19, 0.66)	0.52 (0.28, 0.94)	<0.01
Model 2 + mutual adjustment	1.00 (reference)	0.51 (0.30, 0.86)	0.36 (0.19, 0.67)	0.53 (0.29, 0.98)	<0.01

Abbreviations: BMI, body mass index; MASLD-PRS, metabolic dysfunction-associated steatotic liver disease-polygenic risk score; PYs, person-years.

[a] Obtained by using multivariable Cox regression model.

[b] Hazard ratios (95% confidence interval) (all such values).

[c] P for trend was calculated across quartiles using multivariable Cox regression models. A two-tailed p <0.05 was considered statistically significant.

Model 1 was adjusted for age, sex, and BMI.

Model 2 was additionally adjusted for total energy intake, smoking status, alcohol intake, educational level, Townsend deprivation index, physical activity, hypertension, diabetes, cancer, cardiovascular disease, fasting duration, spectrometer, MASLD-PRS, first 10 principal components of ancestry, and genotype measurement batch.

Model 2 + mutual adjustment was additionally included both EAT-Lancet diet index and the metabolic signature of EAT-Lancet diet simultaneously in the model2 to examine association independence.

Table S8. Associations between the recomputed EAT-Lancet diet score and risk of MASLD in the UK Biobank

Excluded component	HR (95 % CI) [a]	P **value**
Vegetables	0.912 (0.856, 0.971)	<0.01
Fruits	0.939 (0.890, 0.991)	0.02
Unsaturated oils	0.952 (0.912, 0.994)	0.03
Legumes	0.962 (0.928, 0.996)	0.03
Nuts	0.971 (0.943, 0.999)	0.04
Whole grains	0.977 (0.955, 0.999)	0.04
Fish	0.980 (0.960, 0.999)	0.04
Beef and lamb	0.982 (0.933, 0.998)	0.03
Pork	0.984 (0.971, 0.998)	0.02
Poultry	0.987 (0.976, 0.998)	0.02
Eggs	0.990 (0.981, 0.999)	0.03
Dairy	0.992 (0.985, 0.999)	0.03
Potatoes	0.993 (0.988, 0.999)	0.03
Added sugar	0.995 (0.990, 0.999)	0.03

Multivariable Cox models adjusted for age, sex, BMI, total energy intake, smoking status, alcohol intake, educational level, Townsend deprivation index, physical activity, hypertension, diabetes, cancer, cardiovascular disease, fasting duration, spectrometer, MASLD-PRS, first 10 principal components of ancestry, and genotype measurement batch.

[a] Hazard ratios (95 % confidence intervals) for per 4-point increase (about one standard deviation). A two-tailed $p < 0.05$ was considered statistically significant.

Table S9. Sensitivity analyses for associations between EAT-Lancet diet index and risk of MASLD [a]

	Categories of the EAT-Lancet diet index				*P* for trend [c]
	Quartile 1	Quartile 2	Quartile 3	Quartile 4	
**EAT-Lancet diet index**					
Sensitivity analysis 1	1.00 (reference)	1.01 (0.86, 1.18) [b]	0.84 (0.70, 1.01)	0.82 (0.67, 0.99)	0.01
Sensitivity analysis 2	1.00 (reference)	1.04 (0.89, 1.21)	0.84 (0.71, 1.00)	0.79 (0.66, 0.95)	<0.01
Sensitivity analysis 3	1.00 (reference)	0.91 (0.74, 1.10)	0.71 (0.57, 0.89)	0.68 (0.53, 0.87)	<0.001
Sensitivity analysis 4	1.00 (reference)	1.01 (0.86, 1.18)	0.82 (0.69, 0.97)	0.76 (0.62, 0.91)	<0.001
Sensitivity analysis 5	1.00 (reference)	1.00 (0.88, 1.13)	0.85 (0.74, 0.97)	0.83 (0.72, 0.95)	<0.01

Abbreviations: MASLD, metabolic dysfunction-associated steatotic liver disease.

[a] The initial model was same as the model 2 in Table2.

[b] Hazard ratios (95% confidence interval) (all such values).

[c] P for trend was calculated across quartiles using multivariable Cox regression models. A two-tailed p <0.05 was considered statistically significant.

Sensitivity analysis 1: further adjusted for baseline albumin, alanine aminotransferase, and gamma-glutamyltransferase.

Sensitivity analysis 2: further adjusted for waist circumference.

Sensitivity analysis 3: excluded participants with less than twice dietary assessment, n = 64,373.

Sensitivity analysis 4: excluded participants with less than 2 years of follow-up, n = 105,660.

Sensitivity analysis 5: expanded the definition of MASLD, n = 105,670, case = 1,724.

Reference

[1] Stubbendorff A, Sonestedt E, Ramne S, Drake I, Hallstrom E, Ericson U. Development of an EAT-Lancet index and its relation to mortality in a Swedish population. Am J Clin Nutr 2022;115:705-716.
[2] Wurtz P, Kangas AJ, Soininen P, Lawlor DA, Davey Smith G, Ala-Korpela M. Quantitative Serum Nuclear Magnetic Resonance Metabolomics in Large-Scale Epidemiology: A Primer on -Omic Technologies. Am J Epidemiol 2017;186:1084-1096.
[3] Soininen P, Kangas AJ, Wurtz P, Suna T, Ala-Korpela M. Quantitative serum nuclear magnetic resonance metabolomics in cardiovascular epidemiology and genetics. Circ Cardiovasc Genet 2015;8:192-206.
[4] Soininen P, Kangas AJ, Wurtz P, Tukiainen T, Tynkkynen T, Laatikainen R, et al. High-throughput serum NMR metabonomics for cost-effective holistic studies on systemic metabolism. Analyst 2009;134:1781-1785.
[5] Lange T, Vansteelandt S, Bekaert M. A simple unified approach for estimating natural direct and indirect effects. Am J Epidemiol 2012;176:190-195.
